# A physiology‐based Earth observation model indicates stagnation in the global gross primary production during recent decades

**DOI:** 10.1111/gcb.15424

**Published:** 2020-12-06

**Authors:** Torbern Tagesson, Feng Tian, Guy Schurgers, Stephanie Horion, Robert Scholes, Anders Ahlström, Jonas Ardö, Alvaro Moreno, Nima Madani, Stefan Olin, Rasmus Fensholt

**Affiliations:** ^1^ Department of Physical Geography and Ecosystem Science Lund University Lund Sweden; ^2^ Department of Geosciences and Natural Resource Management University of Copenhagen Copenhagen Denmark; ^3^ School of Remote Sensing and Information Engineering Wuhan University Wuhan China; ^4^ Global Change Institute University of the Witwatersrand Johannesburg South Africa; ^5^ Center for Middle Eastern Studies Lund University Lund Sweden; ^6^ Image Processing Laboratory (IPL) Universitat de València Paterna, València Spain; ^7^ Numerical Terradynamic Simulation Group, W.A. Franke College of Forestry & Conservation University of Montana Missoula MT USA; ^8^ Jet Propulsion Laboratory Pasadena CA USA

**Keywords:** climate change, Earth system, GIMMS, land‐atmosphere interactions, light use efficiency, photosynthesis, vegetation productivity

## Abstract

Earth observation‐based estimates of global gross primary production (GPP) are essential for understanding the response of the terrestrial biosphere to climatic change and other anthropogenic forcing. In this study, we attempt an ecosystem‐level physiological approach of estimating GPP using an asymptotic light response function (LRF) between GPP and incoming photosynthetically active radiation (PAR) that better represents the response observed at high spatiotemporal resolutions than the conventional light use efficiency approach. Modelled GPP is thereafter constrained with meteorological and hydrological variables. The variability in field‐observed GPP, net primary productivity and solar‐induced fluorescence was better or equally well captured by our LRF‐based GPP when compared with six state‐of‐the‐art Earth observation‐based GPP products. Over the period 1982–2015, the LRF‐based average annual global terrestrial GPP budget was 121.8 ± 3.5 Pg C, with a detrended inter‐annual variability of 0.74 ± 0.13 Pg C. The strongest inter‐annual variability was observed in semi‐arid regions, but croplands in China and India also showed strong inter‐annual variations. The trend in global terrestrial GPP during 1982–2015 was 0.27 ± 0.02 Pg C year^−1^, and was generally larger in the northern than the southern hemisphere. Most positive GPP trends were seen in areas with croplands whereas negative trends were observed for large non‐cropped parts of the tropics. Trends were strong during the eighties and nineties but levelled off around year 2000. Other GPP products either showed no trends or continuous increase throughout the study period. This study benchmarks a first global Earth observation‐based model using an asymptotic light response function, improving simulations of GPP, and reveals a stagnation in the global GPP after the year 2000.

## Graphical abstract

A novel modelling approach for Earth observation of global gross primary production (GPP) of vegetation was introduced. The model improved simulations of GPP, and revealed a stagnation in the global GPP after the year 2000.

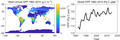

## 1 INTRODUCTION

Carbon (C) fixation via photosynthesis is known as gross primary production (GPP) when assessed at the ecosystem level, and together with ecosystem respiration it dominates the land‐atmosphere exchange of carbon dioxide (CO_2_) (Ryu et al., 2019). GPP is thus one of the main processes driving climate regulation, C sequestration and C storage (Le Quéré et al., 2018) as well as being an important proxy for a range of ecosystem services, including the production of food, fibre and fuel, and the regulation of the habitability of Earth (Rockström et al., 2009; Steffen et al., 2015). Human population growth and increased food and fibre consumption have increased the use of global primary production (Krausmann et al., 2017), with severe consequences for services and functions of global ecosystems (Rockström et al., 2009; Steffen et al., 2015). An improved understanding of spatial and temporal dynamics in regional and global GPP is additionally essential to better understand, quantify and forecast the effects of current and future climate change (Tagesson et al., 2020), also in relation to design of climate change mitigation policies and in the early detection of ecosystem change (IPBES, 2018).

Models driven by satellite‐based Earth observation data are commonly used for quantifying GPP for large areas, allowing to evaluate the role of GPP within the global carbon cycle (Ciais et al., 2013). The most common approach for estimating GPP from satellite data involves the light use efficiency (LUE) model, where LUE is defined as the conversion efficiency of absorbed photosynthetically active radiation (PAR) into GPP (Madani et al., 2014; Monteith, 1972, 1977; Potter et al., 1993; Ruimy et al., 1994; Running et al., 2004). The LUE concept has been applied in various methods, either by using a biome‐specific LUE (Ruimy et al., 1994) modified using meteorological variables (Running et al., 2004), or modified using plant traits (Madani et al., 2014). The LUE model can be formulated as the product of incoming radiation, the fraction of PAR absorbed by the vegetation (FAPAR), and the LUE; where LUE and PAR provide the relationship between GPP and light, whereas FAPAR incorporates dynamics in green vegetation cover.

The advantage of the LUE model is its simplicity using a linear relationship, and it has been shown to work well in a range of biomes and environmental conditions (Kolby Smith et al., 2015; Martínez et al., 2020; Running et al., 2004; Ryu et al., 2019; Stocker et al., 2019; Tagesson, Mastepanov, et al., 2012). However, this simplicity also comes with a cost. For the assumption of a linear relationship to be valid, the intercept between GPP and absorbed PAR must equal zero, as otherwise a systematic error is introduced. Observations show that the relationship between GPP and PAR generally follows an asymptotic shape, and only by multiplying with FAPAR does the relationship turn approximately linear (Baldocchi, 1994; Falge et al., 2001; Lindroth et al., 2007; Monteith, 1972; Ruimy et al., 1995; Tagesson, Fensholt, Cropley, et al., 2015). However, when FAPAR approaches one, this linearization does not occur, and the actual relationship thereby remains asymptotic. Furthermore, the LUE‐derived GPP strongly depends on FAPAR, as it sets the maximum level of absorbed PAR, and thereby also the maximum level of GPP during a certain phenological phase. FAPAR is generally defined as PAR absorbed by green vegetation, but there is yet no exact definition of ‘green’ in this context. In reality, PAR is strongly absorbed by all components of the canopy, and an estimate of ‘green FAPAR’ is therefore impossible to verify based on ecosystem‐scale field observations. Finally, there is a hysteresis in the GPP‐absorbed PAR relationship, since LUE is not the same during different phases of the phenological cycle (Madani et al., 2014; Ryu et al., 2019; Tagesson, Fensholt, Cropley, et al., 2015).

Global‐scale Earth observation‐based GPP products are of high societal relevance, and current products are based either on LUE, or machine learning techniques (Booth et al., 2012; Jung et al., 2011; Kolby Smith et al., 2015; Running et al., 2004; Stocker et al., 2019; Yao Zhang et al., 2017). To better accommodate for the nonlinear relationship known to exist between PAR and carbon assimilation, we take an ecosystem‐level physiologically realistic approach by instead converting Earth observations to biome‐specific parameters of an asymptotic relationship between GPP and PAR, referred to as the light response function (LRF). We further analyse the impact of local air temperature (*T*_air_), soil water content (SWC), vapour pressure deficit (VPD) and atmospheric CO_2_ concentrations on GPP, and test relevant relationships to constrain LRF‐modelled GPP. We then apply these relationships biome‐wise to derive spatially explicit estimates of GPP globally. The new GPP estimates derived using the LRF model are cross‐validated against ground observations and compared to a set of state‐of‐the‐art Earth observation GPP products. Finally, the LRF‐based GPP product is used for quantifying dynamics in global‐scale GPP budgets over the period 1982–2015 and the contribution from different terrestrial regions to the global GPP.

## 2 MATERIALS AND METHODS

### 2.1 Data collection and pre‐processing

#### 2.1.1 Gridded data

##### NDVI and land cover data

The Global Inventory Monitoring and Modelling System third generation (GIMMS3g) data archive is based on observations by the advanced very high resolution radiometer (AVHRR) (Pinzon & Tucker, 2014). GIMMS3g provides global semi‐monthly normalized difference vegetation index (NDVI) at 1/12° spatial resolution for the period 1981 to 2015. Among all existing long‐term AVHRR‐based datasets, the GIMMS3g NDVI time series has been shown to have the highest temporal consistency, decreasing the risk of artefacts caused by sensor shifts (Tian et al., 2015).

The MCD12C1 version 6 land cover type product (land cover as defined by the International Geosphere Biosphere Programme, IGBP) is derived from Moderate Resolution Imaging Spectroradiometer (MODIS) observations based on a combined dataset of Terra and Aqua platforms. It provides the dominant land cover type at a spatial resolution of 0.05° × 0.05°. No global dynamic IGBP dataset exists for the full period 1982–2015, and we therefore used the MCD12Q1 product for 2001 (representing the middle of our study period). The multiple IGBP classes were simplified to eight ‘biomes’ by merging the following land cover classes: closed shrublands (IGBP class 6), open shrublands (7) and woody savannahs (8) were merged with the class savannahs (9). Permanent wetlands (11) were merged with grasslands (10). The cropland/natural vegetation mosaic class (14) was combined with croplands (12). Urban (13), snow and ice (15), and barren or sparsely vegetated (16) were combined. The final biomes recognised were evergreen needleleaf forest; evergreen broadleaf forest; deciduous needleleaf forest; deciduous broadleaf forest; mixed forest; savannah/shrublands; grasslands; and croplands.

##### Ancillary meteorological data

We downloaded daily European Centre for Medium‐Range Weather Forecasts (ECMWF) Reanalysis (ERA)‐interim *T*_air_ at 2 m height (in degrees K), volumetric soil water in 0–0.07 m soil depth (m^3^ m^‐3^), downwelling shortwave solar radiation at the surface (W/m^2^), and total column water vapour (kg/m^2^) from 1982 to 2015. Data had a spatial resolution of 0.25° × 0.25°, and were available for every 12 hr period (daytime and night‐time) (Dee et al., 2011; ECMWF, 2016), which were averaged to daily values. PAR (W/m^2^) was estimated as 46% of the downwelling shortwave radiation (Iqbal, 1983).

All gridded data were resampled to 0.05° × 0.05° resolution using bilinear interpolation to be collocated and having identical spatial resolutions.

##### Global‐scale GPP and solar‐induced fluorescence products

In order to compare our modelled GPP against previous GPP products, we included six state‐of‐the‐art GPP products based on Earth observation data (Table 1). Based on data from MODIS, we used: the MOD17GPP collection 5.5 (Running et al., 2004), the Soil Moisture Active Passive (SMAP) GPP product (Booth et al., 2012) and the Vegetation Photosynthesis Model (VPM) GPP product (Zhang et al., 2017). Based on GIMMS3g NDVI we used: GPP as modelled by the P‐model (Stocker et al., 2019), GPP from FLUXCOM (Jung et al., 2011) and GPP modelled with a MOD17‐based algorithm by Kolby Smith et al. (2015).

**Table 1:** Overview of global‐scale gross primary production (GPP) and Solar‐Induced Fluorescence (SIF) products. The products of GPP are: light response function (LRF)‐modelled GPP developed in this study, GPP from FLUXCOM, Kolby Smith‐modelled GPP using the MOD17‐based algorithm, GPP as modelled by the P‐model, the MOD17GPP, the Soil Moisture Active Passive (SMAP) GPP and the Vegetation Photosynthesis Model (VPM) GPP. For SIF, we used the contiguous SIF (CSIF) dataset. Information of spatial and temporal resolution, study period of the products and their references are included

Product	Spatial resolution	Temporal resolution	Study period	Reference
LRF	0.05° × 0.05	Daily	1982–2015	(This study)
FLUXCOM	0.5° × 0.5	Monthly	1982–2015	Jung et al. (2011)
Kolby Smith	0.5° × 0.5	Monthly	1982–2015	Kolby Smith et al., (2015)
P‐model	0.5° × 0.5	Daily	1980–2013	Stocker et al., (2019)
MOD17	0.5° × 0.5	Monthly	2000–2016	Running et al., (2004)
SMAP	9 × 9 km^2^	Daily	2000–2016	Booth et al., (2012)
VPM	0.05° × 0.05	8‐day	2000–2016	Zhang et al., (2017)
CSIF	0.05° × 0.05	4‐day	2000–2017	Zhang et al., (2018)

Solar‐induced fluorescence (SIF) is light emitted during photosynthesis, and thereby supposedly a proxy for GPP (Zhang et al., 2018). We used a contiguous global coverage product (CSIF), developed from Orbiting Carbon Observatory‐2 (OCO2) SIF data by training a neural network using MODIS surface reflectance data (Zhang et al., 2018). Dynamics in CSIF are consistent with SIF from OCO‐2 and the Global Ozone Monitoring Experiment‐2 (GOME‐2), but have a longer time series and the same spatial resolution as used in this study (Table 1).

#### 2.1.2 Site data

##### Eddy covariance data and atmospheric CO_2_ concentrations

Daily GPP observations (partitioned with the daytime methods, with variable user threshold, i.e. the variable called GPP_DT_VUT_MEAN) were acquired from the Tier 1 and Tier 2 database of FLUXNET 2015 (Pastorello et al., 2020). FLUXNET is a global network of micrometeorological flux measurement sites measuring net CO_2_ exchange using the eddy covariance method. FLUXNET2015 collated measurements from 212 of these sites into an open database. To ensure an accurate point‐to‐pixel comparison, only sites measuring fluxes over the same land cover as the dominant cover of the associated MCD12C1 pixels were included. Additionally, ten sites located close to confounding elements for NDVI, such as rivers, oceans or towns were disregarded. In this study, 79 of the 212 sites were used (Table S1). From the selected sites, only GPP with high‐quality control flags (>0.5) was used in the final analysis.

Atmospheric CO_2_ concentrations derived from in situ air measurements at Mauna Loa, Hawaii were retrieved from the NOAA Earth System Research Laboratories (ESRL) (Keeling et al., 2005).

##### NPP data

To further evaluate the modelled GPP data against an independent ground observation dataset, we downloaded the net primary productivity (NPP) estimates compiled by the Global Primary Production Data Initiative (GPPDI) (Olson et al., 2013). From the GPPDI version B dataset, we extracted NPP from all sites which had an uncertainty flag value lower than 50. In total, it was 157 site‐years distributed globally across 58 sites between 1982 and 1998 (Olson et al., 2013).

### 2.2 Data handling

#### 2.2.1 Time series of Earth observation data

We used the TIMESAT software (Jönsson & Eklundh, 2004) in which we applied the double logistic fitting method for gap‐filling and smoothing of the quality‐filtered GIMMS3g NDVI time series. The double logistic method smooths the seasonal curves based on intervals around maxima and minima in the time series, resulting in low sensitivity to noise. This method was reported to perform best on time series data where quality is not well known, and where noise can be introduced for instance by long periods of snow cover and persistent cloud cover (Beck et al., 2006; Jönsson & Eklundh, 2004; Zeng et al., 2011). The parameters applied in TIMESAT were based on visual interpretation of the NDVI time series of the FLUXNET2015 sites (Jönsson & Eklundh, 2004): seasonal parameter = 0.5, number of envelope iterations = 2, adaptation strength = 2, Savitzky–Golay window size = 4. To remove outliers from the NDVI time series, a spike method of median filter was employed in TIMESAT with the spike parameter set to two.

#### 2.2.2 Estimates of photosynthetic capacity and quantum efficiency

The effect of incoming PAR on GPP at an ecosystem level was modelled using the physiologically realistic nonlinear Mitscherlich light‐response function (LRF) following (Falge et al., 2001; Lindroth et al., 2007; Tagesson et al., 2017):

(1) $$\text{GPP}=‐\left(F_{\text{opt}}\right)\times \left(1‐\exp^{\left(\frac{‐\alpha \times \text{PAR}}{F_{\text{opt}}}\right)}\right),$$

where *F*_opt_ is the optimized carbon uptake at light saturation which is the maximum level at which photosynthesis can operate (photosynthetic capacity; g C m^−2^ day^−1^), and *α* is the quantum efficiency (g C day^−1^ W^−1^ PAR), representing the initial slope of the light response curve.

For each of the selected FLUXNET2015 sites and each semi‐monthly period available, we extracted the 95th percentile of daily GPP as an estimate of *F*_opt_. We estimated *α* as the median of daily ratios of GPP and incoming PAR for all days with an average daily incoming PAR of <25 W/m^2^. Occasionally, daily *α* estimated in this way was unrealistically high, and values of *α* larger than 0.25 g C day^−1^ W^−1^ were excluded.

### 2.3 Relationships with explanatory variables

**Figure 1:**
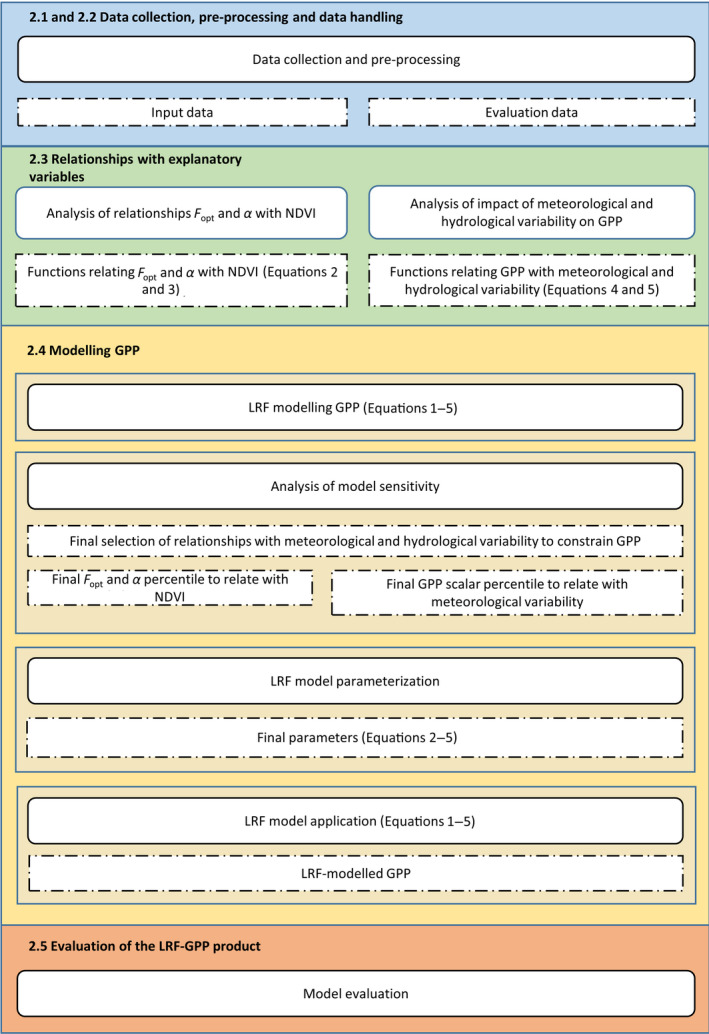
Overview of the workflow of the model development and evaluation. Rectangles with rounded corners present methods whereas rectangles with dashed borders are output of these. Coherent steps of the workflow are colour‐coded, and referred to by their respective subheadings. *F*_opt_ is the optimized carbon uptake at light saturation (photosynthetic capacity; g C m^−2^ day^−1^), and *α* is the quantum efficiency (g C W^‐1^ PAR)

For an overview of the workflow of the model development, see Figure 1.

#### 2.3.1 Coupling photosynthetic capacity and quantum efficiency with NDVI

In order to spatially and temporally extrapolate GPP; we parameterized logistic relationships between the parameters of the LRF model (*F*_opt_ and *α* in Equation 1) and GIMMS3g NDVI for each biome:

(2) $$F_{\text{opt}}=\frac{F_{\text{optmax}}}{1‐\exp(b_{F_{\text{opt}}}\times \text{NDVI}+F_{\text{optmid}})},$$

(3) $$\alpha =\frac{\alpha_{t\max}}{1‐\exp(b_{\alpha }\times \text{NDVI}+\alpha_{\text{mid}})},$$

where *F*_optmax_ and *α*_max_ are the maximum possible *F*_opt_ and *α* for the specific land cover class, *F*_optmid_ and *α*_mid_ are parameters of the logistic functions denoting the NDVI midpoint of the function, and *b*_fopt_ and b_*α*_ represent the steepness of the curve. A logistic function was chosen as it turned out to describe the response of *F*_opt_ and *α* to NDVI well (Section 3.1).

**Figure 2:**
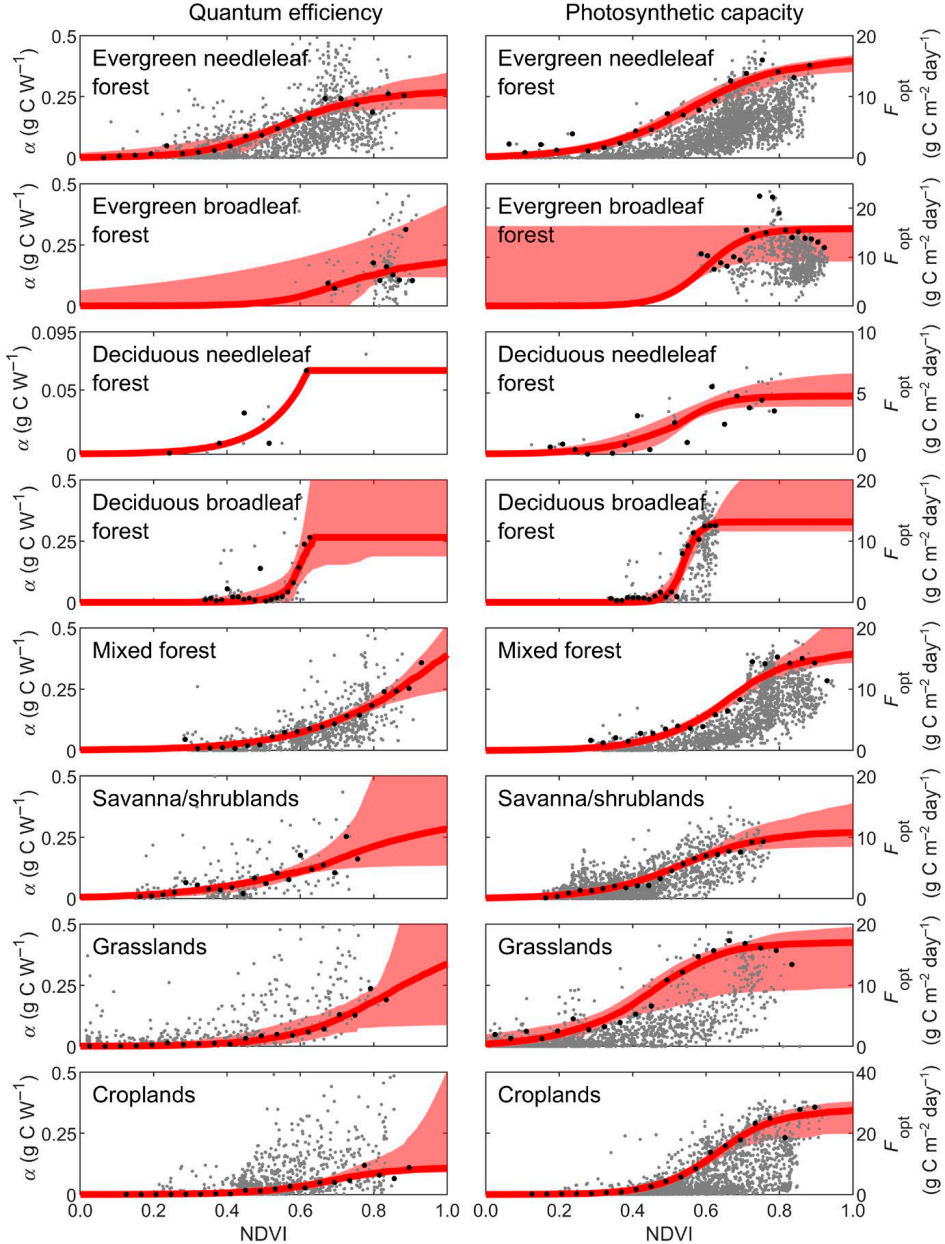
Relationship between quantum efficiency (*α*) and photosynthetic capacity (*F*_opt_) and the normalized difference vegetation index (NDVI). Shown are: all data (grey dots), percentiles of GPP extracted from NDVI bins (biome‐wise percentiles given in Table 2; black dots), and the fitted logistic relationships (red lines) including the 5^th^ to 95^th^ confidence interval obtained from bootstrapping (shaded area)

In the first estimates of *F*_opt_ and *α* (Section 2.2.2 above), a large fraction of the variability was caused by spatial and temporal variability in biogeophysical conditions. Therefore, in a model optimization procedure, we used biome‐specific percentiles of *F*_opt_ and *α* for 20 equally sized NDVI bins when fitting the functions. We fitted all *F*_opt_ and *α* percentiles (1–100) and modelled GPP for each *F*_opt_ and *α* percentile combination (Section 2.4.2 below). The final biome‐specific percentile used was the one generating the lowest root‐mean‐square‐error (RMSE) in the subsequent model evaluation (Figure 2; Table 2).

#### 2.3.2 Impact of meteorological and hydrological variability on GPP

In order to derive relationships used for constraining GPP within the modelling framework (Figure 1), we analysed impacts of daily variability in meteorological and hydrological conditions (T_air_, CO_2_, SWC and VPD) on daily GPP. We clustered the daily FLUXNET2015 GPP by separating the meteorological and hydrological data into bins (bin width: T_air_: 5°C; SWC 5%Vol; CO_2_ 5 ppm; VPD 25 hPa) and extracted the 50th and 98th percentile GPP associated with each bin. The 50th percentile indicates the median impact of the meteorological and hydrological variables, whereas the 98th percentile indicates the upper boundary for GPP.

A large fraction of the GPP variability caused by meteorological and hydrological variability is already captured by the NDVI variability, and is therefore already included in the GPP modelled by Equations (1), (2), (3). In order to estimate GPP that is *not* captured by Equations (1), (2), (3), we calculated a GPP scalar by taking the ratio of measured GPP and GPP modelled using Equations (1), (2), (3). This is considered to be GPP variability caused by short‐term meteorological and hydrological variability, whereas Equations (1), (2), (3) capture seasonal dynamics in GPP caused by phenological drivers. We first used regression tree analysis to determine the variable importance of the meteorological and hydrological variables (Supplementary subsection S2). The environmental variables were introduced in the GPP model in the order of the variable importance for the different biomes. We thereafter analysed impact of daily variability in meteorological and hydrological conditions (*T*_air_, CO_2_, SWC and VPD) on the GPP scalars. These GPP scalars were also separated using the meteorological and hydrological bins described above and the 50th and 98th GPP scalar percentiles were extracted from each bin.

The relationships between the GPP and *T*_air_, and GPP and VPD turned out to follow logistic curves (Section 3.1; Supplementary subsection S3). We therefore fitted such a function with 50th and 98th percentile binned GPP, and 50th and 98th percentile GPP scalar for each biome:

(4) $$f\left(\text{ENV}\right)=\frac{a_{\text{max}}}{1‐\exp(k\times \text{ENV}+b_{\text{mid}})},$$

where *f*(ENV) is a function for constraining GPP due to variability in the meteorological variables (ENV; in this case *T*_air_, and VPD). *a*_max_, *k* and *b*_mid_ are parameters of the logistic function giving the maximum value of the function, steepness of the curve and the ENV value at the midpoint of the function respectively.

The relationship between GPP and SWC turned out to follow a bell‐shaped curve, and we thereby fitted a Gaussian function as it turned out to describe the response with 50th and 98th percentile binned GPP, and 50th and 98th percentile GPP scalar well (Section 3.1; Supplementary Subsection S3):

(5) $$f\left(\text{SWC}\right)=a_{\text{max}}\times \exp\frac{{\left(‐\text{SWC}‐b_{\text{mid}}\right)}^{2}}{2\times b_{\sigma }},$$

where *f*(SWC) is a function to constrain GPP variability due to SWC stress. *a*_max_, *b*_mid_ and *b_σ_* are parameters of the Gaussian function giving the peak value of the curve, the position at the centre of the peak, and the width of the curve (i.e. the standard deviation of the response of GPP to a change in SWC) respectively.

### 2.4 Modelling GPP

#### 2.4.1 Constraining modelled GPP by meteorological and hydrological stress

Based on the results of the analysis of the impact of meteorological and hydrological variables on GPP (Sections 2.3.2 and 3.1), we included constraining scalars in the GPP model that were optimized per biome (Figure 1; Supplementary Subsection S4). When strong relationships between GPP and the meteorological and hydrological variables were observed, but at the same time, no relationship between the GPP scalar and a variable was found; this indicated that the GPP model already captured the variability caused by that specific variable in its variability with NDVI and PAR (Equations (1), (2), (3)). In those cases, no scalar was included in the model. If no relationship to a meteorological or hydrological scalar was found, whereas there was a strong relationship between the upper boundary of GPP (the 98th percentile) and the meteorological or hydrological variables, we included a model of the upper boundary of GPP (i.e. a value that modelled GPP cannot exceed) (Supplementary Subsection S3–S4).

#### 2.4.2 Model sensitivity analysis

We tested the sensitivity of GPP output to F_opt_ and *α* variability by extracting 1%–100% semi‐monthly F_opt_ and *α* percentiles for 20 equally sized NDVI bins before fitting the functions. The models were fitted with these 100 F_opt_ and *α* estimates, generating different GPP outputs and model statistics depending on F_opt_ and *α* variability. We also tested the sensitivity of the model to the variability in the GPP scalars by extracting the 1–100 percentiles of the scalars from each meteorological and hydrological variable bins before fitting the selected functions (Equations 4 and 5). A scalar can only decrease GPP, and scalars larger than one were therefore set to one.

#### 2.4.3 Model parameterization and application

From the model sensitivity analysis, the percentiles that generated the lowest RMSE were used in the final biome‐specific model parameterization (Figure 1; Table 2). For the parameterization and evaluation of the models, we used bootstrap simulations with 200 iterations. For each iteration, some of the FLUXNET2015 sites were included and some were omitted. After the site exclusion process described above (Section 2.1.2), deciduous needleleaf forest and deciduous broadleaf forest had only one site each. Therefore, site years were instead included or omitted within the bootstrap simulations for these biomes. The bootstrap simulations generated a set of 200 model parameters. Modelled GPP parameterized from the site data included in the bootstrap simulations was evaluated by calculating the RMSE and by fitting an ordinary least square linear regression between simulated GPP and observed GPP from the omitted sites.

Average values and standard deviations were calculated from the model parameters estimated by the 200 bootstrap simulations (Supplementary Subsection S6; Table S3–S5). The average parameters were used in the final spatial and temporal upscaling of GPP, and their standard deviations provide parameter uncertainties. As input data to the GPP model, we used semi‐monthly NDVI, daily PAR, daily *T*_air_, daily SWC and daily VPD. This modelled GPP is hereinafter called LRF‐GPP, after the light response function (Equation 1).

### 2.5 Evaluation of the LRF‐GPP product

The LRF‐GPP was compared with other Earth observation‐based global GPP products by evaluating the ability to capture spatial, inter‐ and intra‐annual variability of the FLUXNET2015 GPP data. However, since the GPP products are originally parameterized using the FLUXNET database, and in order to do a truly independent evaluation, the inter‐annual variability of GPP was also evaluated against NPP data from the GPPDI sites. The GPPDI data were collected prior to 1998, and this evaluation was therefore only performed for the GIMMS3g NDVI products. CSIF data were also extracted for all FLUXNET2015 sites, and all GPP products were evaluated against spatial, inter‐ and intra‐annual dynamics in the CSIF data. The agreement between the GPP products and these datasets was evaluated by fitting ordinary least‐square linear regressions and by calculating their RMSE.

## 3 RESULTS

### 3.1 Relationships with explanatory variables

#### 3.1.1 Photosynthetic capacity and quantum efficiency in relation to NDVI

The relationships between *F*_opt_ and *α* and NDVI are highly variable and we therefore extracted percentiles of *F*_opt_ and *α* for NDVI bins before studying the relationships (Figure 2). Strong logistic relationships then emerged between binned *F*_opt_ and *α* and NDVI for all biomes (Figure 2; Table S3). Only few estimates of *α* were available for the deciduous needleleaf and deciduous broadleaf biomes, and the fitted relationships generated unrealistically large *α* values when NDVI was high. Maximum binned *α* were therefore used as a limit (Figure 2).

**Figure 3:**
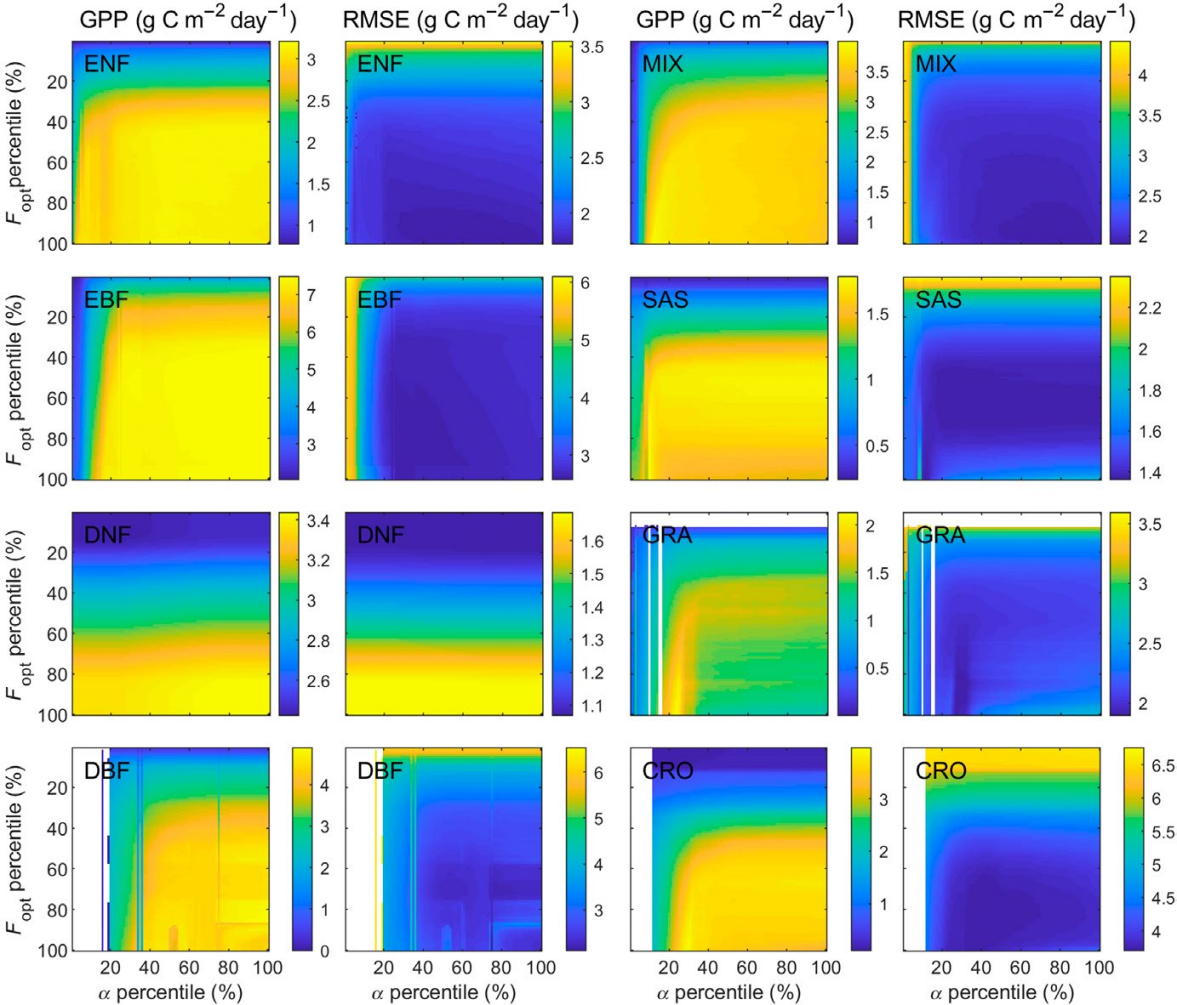
Sensitivity of modelled gross primary production (GPP) to variability in optimized carbon uptake at light saturation (*F*_opt_) and quantum efficiency (*α*). Shown is the impact on GPP and the root‐mean‐square‐error (RMSE) for: evergreen needleleaf forest (ENF); evergreen broadleaf forest (EBF); deciduous needleleaf forest (DNF); deciduous broadleaf forest (DBF); mixed forest (MIX); savannah/shrublands (SAS); grasslands (GRA); and croplands (CRO). White areas are caused by too few data in the bins. Note that scales differ for the different biomes. For coefficient of determination (*R* ^2^) and slope and intercept from ordinary least square linear regression fitted between modelled and field measured GPP, see Supplementary Subsection S5

The sensitivity test indicated that LRF‐GPP is substantially more sensitive to variation in *F*_opt_ than *α* (Figure 3; Supplementary Subsection S5). The sensitivity was also larger at lower *F*_opt_ and *α* percentiles, as the upper percentiles GPP are instead constrained by the environmental scalars. From this sensitivity space, the *F*_opt_ and *α* percentiles generating the lowest RMSE can be extracted, and these percentiles were used in the final model parameterization.

#### 3.1.2 Impact of meteorological and hydrological variability on GPP

**Figure 4:**
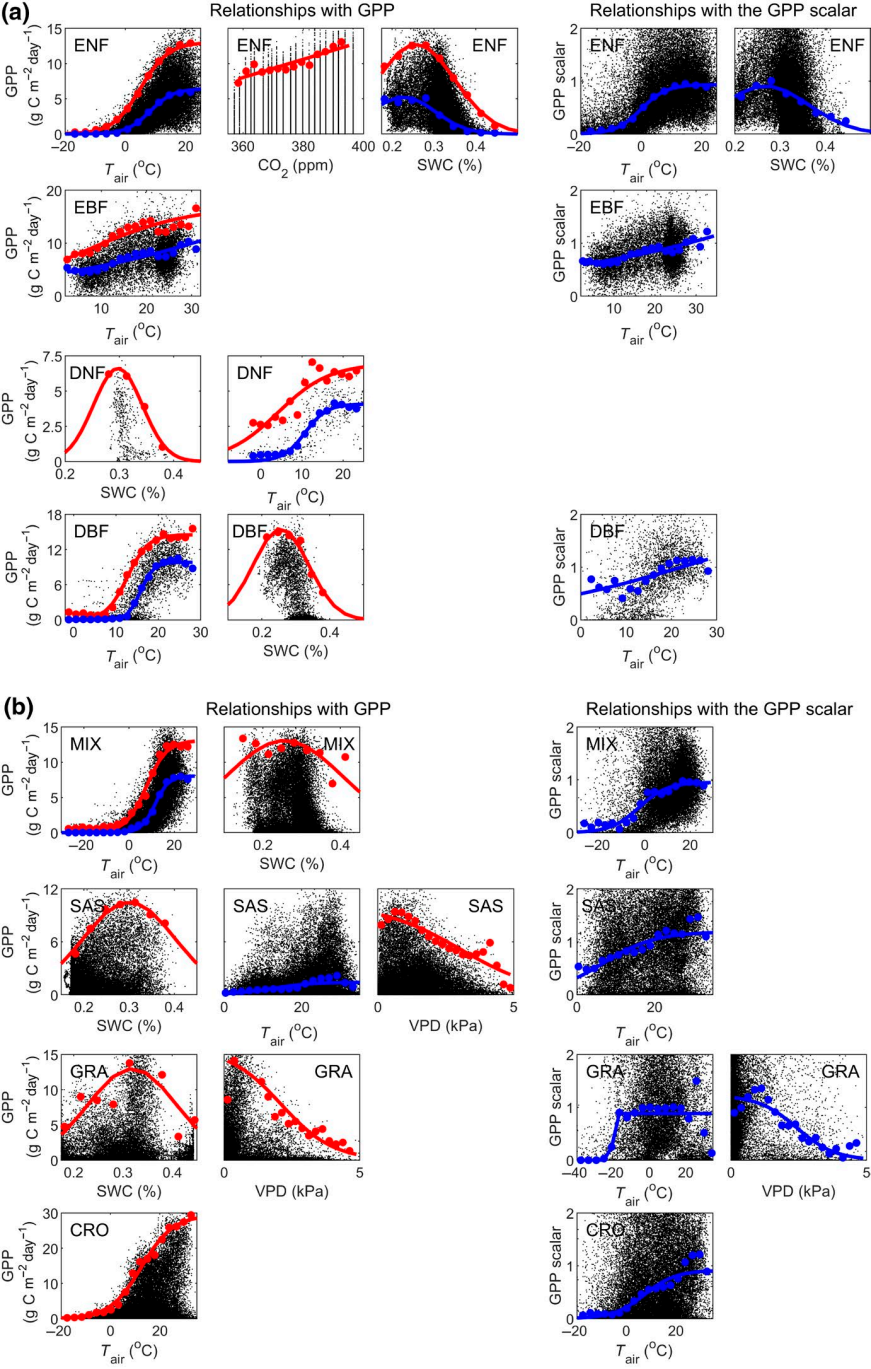
Relationships between gross primary production (GPP) and the constraining environmental variables: air temperature (*T*_air_), soil water content (SWC), atmospheric carbon dioxide (CO_2_) concentrations and vapour pressure deficit (VPD). Shown are relationships for (a) the biomes: evergreen needleleaf forest (ENF); evergreen broadleaf forest (EBF); deciduous needleleaf forest (DNF); deciduous broadleaf forest (DBF); and (b) the biomes: mixed forest (MIX); savannah/shrublands (SAS); grasslands (GRA); and croplands (CRO). Only those constraining variables with strong relationships against median GPP (blue), upper GPP boundary (98th percentile; red) and against the median GPP scalars are included. For all relationships, see Supplementary Information Subsection S3

Strong relationships between median binned GPP, and *T*_air_, VPD and SWC exist (Figure 4; Supplementary Subsection S2–S3). Generally, most of the variability was explained by one dominant factor and the remaining variables explained substantially less (Table S2). After NDVI and PAR, *T*_air_ was the dominant constraining variable explaining GPP variability in most biomes (Figure 4; Supplementary Subsection S2–S3). For all biomes except savannah/shrublands and grasslands, upper boundaries of GPP were also found against *T*_air_ (Figure 4; Supplementary Subsection S4). The second most important variable was SWC, where upper boundaries of GPP were observed for all biomes except for evergreen broadleaf forest and croplands (Figure 4; Supplementary Subsection S3). The optimal SWC level was quite similar among biomes, ranging between 26% and 31% (Table S5). The widest range in the response of GPP to SWC was found for mixed forest (Figure 4; Table S5, as indicated by the standard deviation of the fitted Gaussian curve). Finally, VPD was found to constrain GPP in savannah/shrublands and grasslands, with associated upper GPP boundaries (Figure 4).

After GPP had been modelled with the light response functions (Equations (1), (2), (3)), which accounted for the variation associated with NDVI and PAR, GPP variability caused by *T*_air_ was still not fully captured for any biome except for deciduous needleleaf forest (Figure 4; subplots against GPP scalars; supplementary subsection S3). The GPP variability caused by SWC and VPD was neither well captured by Equations (1), (2), (3) for evergreen needleleaf forest and grasslands respectively (Figure 4).

**Figure 5:**
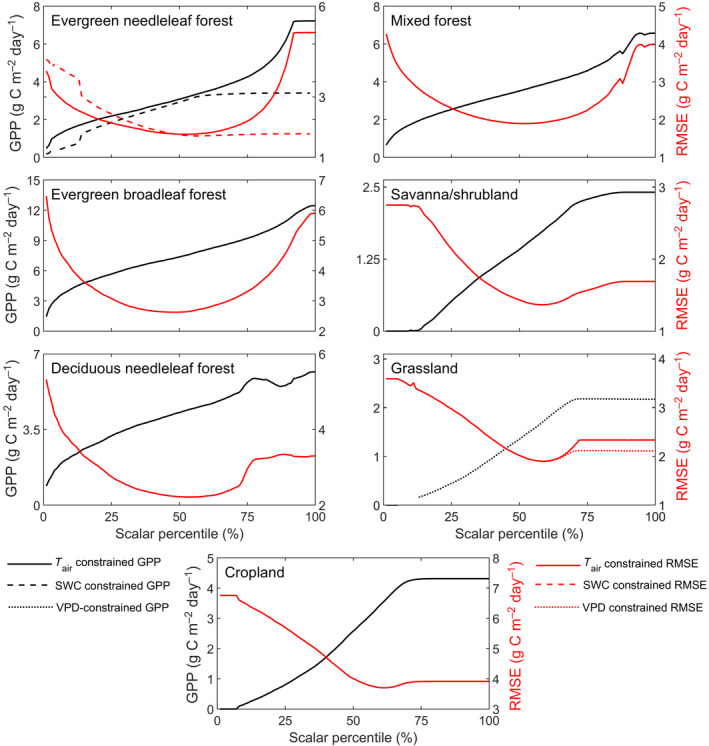
Sensitivity of the gross primary production (GPP) model to variability in environmental scalars (air temperature (*T*_air_), soil water content (SWC) and vapour pressure deficit (VPD)). The *T*_air_ scalars were applied for all biomes except for deciduous needleleaf forest. For evergreen needleleaf forest and grasslands, a SWC and a VPD scalar were additionally applied respectively. Note that scales differ for the different biomes. The percentiles of each scalar were extracted and the constraining models were fitted. Shown is the impact of this percentile fitting on GPP (black), and root‐mean‐square‐error (RMSE) (red). For remaining statistics, see Supplementary Information Subsection S5.2

The sensitivity analysis for LRF‐GPP against variability in the *T*_air_, SWC and VPD scalars indicated that the percentiles generating the lowest RMSE value were relatively close to the median values (Figure 5). At the lower and higher percentiles, RMSE values were substantially larger (Figure 5). From this sensitivity test, the *T*_air_, SWC and VPD scalar percentiles generating the lowest RMSE were extracted, and these percentiles were used in the final model parameterization. The SWC and VPD scalars did not further reduce the lowest RMSE for evergreen needleleaf forest and grasslands respectively (Figure 5). These scalars were therefore excluded from the final model. The upper boundary of CO_2_ for evergreen needleleaf forest (Figure S1), decreased the model performance (data not shown) and was therefore also excluded.

### 3.2 Model evaluation

The model evaluation indicates that the LRF‐GPP captured the variability in field observations relatively well for all biomes except evergreen broadleaf forest (Table 2), where the average GPP was still well captured but the variability was underestimated (as indicated by slope and intercept in Table 2; Figure S24).

**Table 2:** Statistics for the bootstrap simulations of gross primary production (GPP; g C m^−2^ day^−1^) and percentiles generating the lowest root‐mean‐square‐errors (RMSE). The uncertainty is ±1 standard deviation from the bootstrap simulations. The numbers in parentheses are the International Geosphere Biosphere Programme (IGBP) land cover classes. *F*_opt_ is the optimized carbon uptake at light saturation (photosynthetic capacity; g C m^−2^ day^−1^), *α* is the quantum efficiency (g C W^−1^ PAR), *T*_air_ is air temperature, and *R* ^2^ is the coefficient of determination

Biome	*F*_opt_ percentile	*α* percentile	*T*_air_ percentile	Mean GPP	Bias	RMSE	Slope	intercept	*R* ^2^
Evergreen Needleleaf Forest (1)	98	76	53	3.2 ± 0.6	−0.00 ± 0.59	1.40 ± 0.19	0.67 ± 0.14	1.10 ± 0.42	0.65 ± 0.09
Evergreen Broadleaf Forest (2)	97	50	50	7.0 ± 1.4	0.10 ± 1.81	1.56 ± 0.58	0.27 ± 0.21	5.18 ± 1.67	0.20 ± 0.15
Deciduous Needleleaf forest (3)	17	50		2.8 ± 0.14	−0.03 ± 0.14	1.16 ± 0.04	0.78 ± 0.04	0.63 ± 0.04	0.61 ± 0.01
Deciduous broadleaf forest (4)	72	76	54	5.9 ± 0.4	−0.30 ± 0.48	2.15 ± 0.39	0.93 ± 0.12	1.21 ± 0.55	0.83 ± 0.07
Mixed forest (5)	97	74	52	3.4 ± 0.71	0.13 ± 1.01	1.52 ± 0.46	0.74 ± 0.25	0.86 ± 0.30	0.72 ± 0.14
Savannah/shrublands (6–9)	61	50	58	1.5 ± 0.55	0.23 ± 0.43	0.88 ± 0.24	0.47 ± 0.15	0.67 ± 0.41	0.52 ± 0.17
Grasslands (10,11)	94	29	57	1.4 ± 0.46	0.28 ± 0.64	1.42 ± 0. 45	0.63 ± 0.23	0.40 ± 0.27	0.48 ± 0.15
Croplands (12)	85	42	61	2.5 ± 0.86	1.08 ± 0.90	2.15 ± 0.80	0.40 ± 0.14	1.06 ± 0.58	0.51 ± 0.17

**Figure 6:**
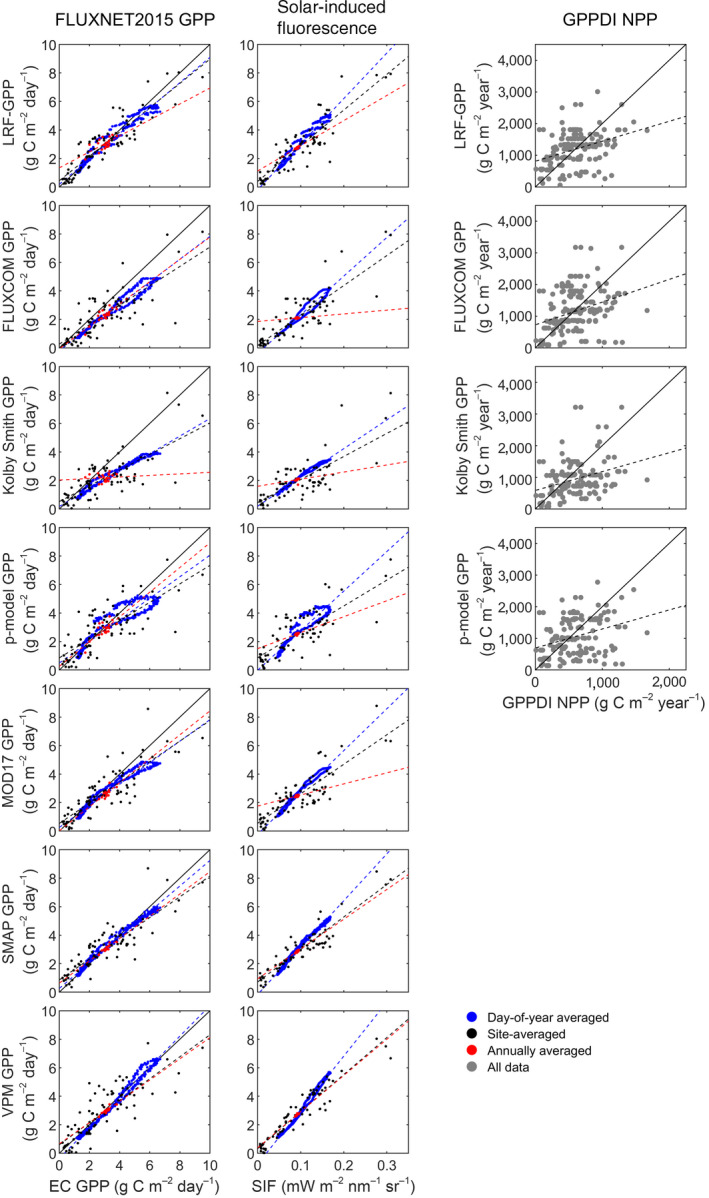
Evaluation of the Earth observation‐based products of gross primary production (GPP). Included comparisons are: eddy covariance (EC)‐based GPP from the 79 FLUXNET2015 sites used in this study; solar‐induced fluorescence (SIF) extracted from the 79 FLUXNET2015 sites; and annual sums of GPP against annual sums of net primary production (NPP) of the Global Primary Production Data Initiative (GPPDI) sites. A one‐to‐one line is included in the FLUXNET2015 comparison whereas in the GPPDI comparison a two‐to‐one line is included since the NPP/GPP ratio is ~0.5 (Landsberg et al., 2020). For statistics of the linear relationships, see Table 3

The evaluation of seven global Earth observation GPP products (including the LRF‐GPP developed in this study) showed that all products captured variability of the FLUXNET2015 GPP well (Figure 6; Table 3; Table S6). Firstly, on global scale, they captured variability well when GPP was averaged across sites, averaged annually and by day‐of‐year (Figure 6; Table 3). The LRF‐GPP captured spatial variability best, SMAP and VPM captured seasonal dynamics best (as given by day‐of‐year average), whereas inter‐annual dynamics were overall best captured by the MOD17 (Figure 6; Table 3; Table S6). Some hysteresis is visible in most of the GPP products (day‐of‐year averages forming circles in Figure 6). Secondly, when separated into biomes, all GPP products captured the variability represented by the FLUXNET2015 GPP well (Table S6). The LRF‐GPP then captured variability better or at least equally well as the other GPP products (Table S6; slope closer to 1.0, and lower RMSE and bias). LRF‐GPP captured the seasonal dynamics well on a global‐scale average (Figure 6), but also agreed well with the seasonal dynamics for the different biomes (Figure S25). The seasonal dynamics were better captured than by the other GIMMS3g NDVI‐based GPP products, and equally well as the MODIS‐based GPP products (Figures S25–S31).

**Table 3:** Statistics for the linear regression analysis comparison between Earth observation‐based and eddy covariance (EC)‐based gross primary production (GPP; g C m^−2^ day^−1^), and Earth observation‐based GPP against solar‐induced fluorescence (SIF; mW m^−2^ nm^−2^ sr^−1^) extracted from the FLUXNET2015 sites. RMSE is the root‐mean‐square‐error and *R* ^2^ is the coefficient of determination

Models		EC GPP			SIF		
	RMSE	Slope	Intercept	*R* ^2^	Slope	Intercept	*R* ^2^
LRF‐GPP							
Spatial	0.02	0.85	0.44	0.82	25.0	0.4	.79
Inter‐annual	0.02	0.56	1.35	0.42	17.7	1.1	.48
Intra‐annual	0.15	0.89	0.19	0.96	32.0	0.2	.97
FLUXCOM							
Spatial	0.62	0.68	0.27	0.63	21.1	0.2	.69
Inter‐annual	0.62	0.78	−0.06	0.71	2.6	1.9	.04
Intra‐annual	0.81	0.80	−0.19	0.97	27.2	−0.4	.97
K Smith							
Spatial	0.78	0.55	0.52	0.56	16.2	0.4	.56
Inter‐annual	0.78	0.05	2.03	0.01	5.0	1.6	.01
Intra‐annual	1.02	0.62	0.17	0.97	20.2	0.2	.99
P‐model							
Spatial	0.13	0.65	0.85	0.60	18.2	0.9	.59
Inter‐annual	0.26	0.87	0.26	0.59	11.2	1.5	.24
Intra‐annual	0.13	0.76	0.50	0.85	27.8	−0.0	.88
MOD17							
Spatial	0.25	0.72	0.54	0.72	20.7	0.5	.74
Inter‐annual	0.25	0.84	0.02	0.55	7.8	1.8	.13
Intra‐annual	0.50	0.76	0.26	0.96	29.4	−0.2	.98
SMAP							
Spatial	0.08	0.73	0.86	0.75	22.8	0.7	.87
Inter‐annual	0.08	0.78	0.64	0.81	20.9	0.9	.62
Intra‐annual	0.05	0.90	0.27	0.98	32.9	−0.2	.99
VPM							
Spatial	0.05	0.77	0.60	0.69	26.0	0.3	.90
Inter‐annual	0.05	0.75	0.66	0.79	25.4	0.4	.73
Intra‐annual	0.14	1.07	−0.36	0.99	38.0	−0.8	.99

In the comparison against SIF data, all products captured both global‐scale spatial, inter‐ and intra‐annual variability well (Figure 6; Table 3). SMAP and VPM captured SIF‐variability better than the GIMMS3g NDVI‐based models (including LRF‐GPP). All products captured the seasonal dynamics in global‐scale average SIF well (Figure 6), and they also agreed well on the seasonal dynamics for the different biomes (Figures S32–S38).

Finally, the variability in GPPDI NPP was relatively well captured by the GIMMS3g NDVI‐based GPP models, even though they underestimated high and overestimated low NPP (Figure 6; Table S7). When assuming that NPP is about half of GPP (Landsberg et al., 2020), the GPP levels were on average fitted well, especially by the LRF‐GPP and the FLUXCOM GPP models (Table S7). Most data points are located close to the 2 to 1 line, indicating that the GPP products agree well with the variability of NPP (Figure 6).

### 3.3 Global patterns revealed by LRF‐GPP

**Figure 7:**
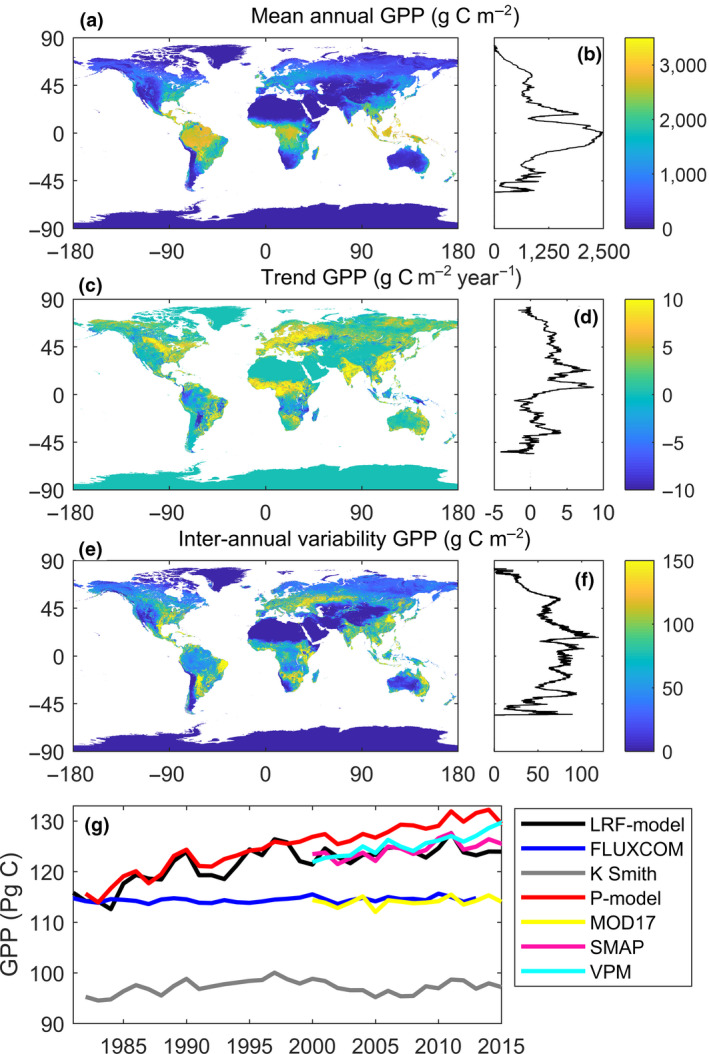
Patterns in light response function (LRF) modelled gross primary production (LRF‐GPP) 1982–2015. (a) Spatial distribution of mean GPP; (b) latitudinal distribution in mean GPP; (c) spatial distribution of trends in GPP 1982–2015; (d) latitudinal distribution in trends in GPP; (e) spatial distribution of inter‐annual variability in de‐trended GPP; (f) latitudinal distribution in inter‐annual variability in de‐trended GPP; and g) a time series 1982–2015 of global‐scale budgets of GPP for the different Earth observation‐based GPP products (see Table 1 for overview of products)

The LRF‐GPP quantifies an annual average global terrestrial GPP budget of 121.8 ± 3.5 Pg C (±1 standard deviation of inter‐annual variability) for the period 1982–2015. High values were found in the areas of tropical evergreen broadleaf forest, whereas arid regions and high northern latitudes have lower average GPP (Figure 7a). The latitudinal distribution of average GPP shows higher values around the equator and a poleward decrease, with a strong drop around 15° North, corresponding to the subtropical deserts.

With the new LRF‐GPP product, a pronounced trend in global‐scale terrestrial GPP was observed with an increase of 0.27 ± 0.02 Pg C year^−1^ (±1 standard error of fitted slope) for the period 1982–2015 (Figure 7g). The latitudinal distribution of the trends is different to the latitudinal distribution of mean GPP, and the trends are generally larger in the northern compared to the southern hemisphere (Figure 7d). The regions with the largest positive trends were located in India, China, Eastern Europe and West Africa (Figure 7c). These regions have substantial areas with croplands, and West Africa recovered from a strong multi‐decadal drought during this period. Large parts of the tropics, especially Papua New Guinea, Indonesia, southern central African woodlands, eastern parts of the dry Miombo woodlands, Gran Chaco and north‐eastern Amazonia showed negative trends in GPP (Figure 7c), as do the semi‐arid regions in central Asia (Figure 7c). Trends in LRF‐GPP were especially strong during the eighties and nineties, whereas the other GPP products show either no trends, or a continuous increase throughout the study period (Figure 7g).

After de‐trending GPP, the average global‐scale inter‐annual variability was 0.74 ± 0.13 Pg C. The regions with the strongest inter‐annual variability were semi‐arid regions in South America, Africa and central Asia (Figure 7c). Cropland areas in China and India also showed strong inter‐annual variability. The areas with most stable GPP between years were hyper‐arid areas and high northern latitudes. The latitudinal distribution in inter‐annual variability indicates that regions between 45°N and 45°S have the highest inter‐annual variability, and variability decreases substantially northwards.

## 4 DISCUSSION

Given the central role of GPP within the Earth system and within climate change mitigation, accurate and continuous monitoring from space is pivotal. Hence, forming an ensemble of Earth observation‐based independent approaches is key in better understanding and addressing the uncertainties in our global GPP budget. In this study, we present a novel GPP product (LRF‐GPP) based on an asymptotic light response function. The model reproduced GPP well, as measured by eddy covariance flux measurements, traditional NPP datasets and against SIF being a GPP proxy. Its bias was within the uncertainty range of both eddy covariance‐based estimates (Schaefer et al., 2012; Tagesson, Ardö, et al., 2016) and those based on coarse spatial resolution Earth observation data (Kimball et al., 2016; Martínez et al., 2020). Nevertheless, there is a large variability in the evaluation datasets, which was not fully captured by the model (Figure 5; Figure S24). Some of the largest uncertainties within the LRF‐model are: the FLUXNET2015 data not being representative for the full range of environmental conditions (thereby generating incorrect model parameterization and assumptions), the scale mismatch between the eddy covariance footprint and the gridded data, the assumption that LRF‐parameters are biome‐dependent and can be characterized using a global land cover classification, uncertainties within the eddy covariance‐derived GPP estimates and within the input data used for the upscaling, cloud contamination in the Earth observation data, differential nutrient availability and diffuse radiation not being considered in this approach, and differentiated land management strategies such as fertilization and irrigation that are not captured (Madani et al., 2014; Martínez et al., 2020; Ryu et al., 2019; Tagesson et al., 2017; Tagesson, Fensholt, et al., 2016). Another important factor omitted in the current model design is land cover change, which may have a strong influence on the trends in the GPP budgets (Supplementary subsection S8). The LRF‐GPP model is a simplified representation of GPP dynamics, and the large day‐to‐day variation in the field cannot be fully captured by a model based on semi‐monthly Earth observations and hydro‐meteorological constraints only. Nevertheless, the LRF‐GPP model captured broad patterns in GPP dynamics and differences between biomes well. To improve the model accuracy, the number of eddy covariance sites and the spatial resolution of the input data could be increased, a dynamic high‐accuracy land cover dataset should be included, and the model parameterization could be separated for spatial, inter‐ and intra‐annual variability.

This is to the authors’ best knowledge, the first attempt to model GPP on global scale using a nonlinear asymptotic LRF model against PAR directly, instead of using a linear LUE relationship and Earth observation‐based FAPAR to convert incoming PAR to absorbed PAR. The main disadvantage of using a nonlinear asymptotic relationship is its scale dependence, as a nonlinear model can only legitimately be applied on the spatial and temporal scale for which it has been parameterized. The classical LUE model does not suffer from such a scale dependence to the same extent, and the linearity assumption at temporal resolutions larger than weekly and at moderate spatial resolutions, has been shown to be reasonable in a range of biomes, and under various environmental conditions (VPM, P‐model, MOD17, SMAP, in Figure 6) (Kolby Smith et al., 2015; Martínez et al., 2020; Running et al., 2004; Stocker et al., 2019; Tagesson, Mastepanov, et al., 2012). However, if the aim is to improve our understanding of the GPP within the Earth system, increasing the spatial and temporal resolution of the GPP estimates is vital. With such an increase, the linearity assumption starts to fail, and we approach the asymptotic relationship between photosynthesis and PAR observed at plant level and at diurnal scales (Cannell & Thornley, 1998; Ruimy et al., 1995). Here, we applied such an asymptotic model with a daily temporal resolution, but the LRF‐GPP model can be reparametrized and applied at a both higher temporal and spatial resolutions than those used in this study.

The strong logistic relationships between both *F*_opt_ and NDVI, and *α* and NDVI (Figure 2), show clear signs of saturation. The NDVI is known to saturate to a degree greater than some other vegetation indices as a result of a saturation of the red band abortion in dense vegetation (Huete et al., 2002; Roujean & Breon, 1995). This causes NDVI to level off above leaf area index values of about 2–5 (Haboudane et al., 2004). It can be argued that this is more problematic for leaf area index and biomass estimates than for GPP estimates, since a saturation of red absorption implies a saturation in photosynthetic radiation absorption, and thus GPP. It could even be so that the use of NDVI to generate FAPAR products explains why the LUE model works so well, since a saturation in the modelled GPP is then inadvertently included via the NDVI saturation. Another benefit of NDVI in relation to vegetation indices less influenced by saturation is that indices sensitive to biomass changes at high biomass loads become less sensitive at low levels (Huete et al., 2002).

Air temperature was the most important constraining variable in most biomes, especially for tropical forest where it was the only variable with a strong impact (Figure 4). Temperature determines physiological, biochemical and metabolic responses and is thus critical for the photosynthetic process. It determines the seasonal dynamics at high latitudes (Parmentier et al., 2011; Tagesson, Mastepanov, et al., 2012; Tagesson, Mölder, et al., 2012), whereas species generally operate close to their upper temperature limit in tropical environments (Pau et al., 2018). The second most constraining variable was SWC with strong effects on plant physiology (Cowan & Farquhar, 1977; Seneviratne et al., 2010; Tewari & Mishra, 2018). Our results indicated a bell‐shaped relationship between upper boundary of GPP and SWC (Figure 4), indicating that GPP is constrained both at low SWC (drought stress) and high SWC (waterlogging stress). Drought stress causes stomatal closure, reduces leaf size, stem extension and root proliferation, and disturbs plant water relations (Farooq et al., 2009). Waterlogging leads to reduced oxygen availability, lower root growth, reduced respiration rates, lower nutrient availability and thereby reduced plant growth (Patel et al., 2014; Tewari & Mishra, 2018). Most Earth observation‐based GPP models do not include the waterlogging effect, and assume that drought stress is sufficiently captured by the FAPAR and the VPD signals (Stocker et al., 2019). Our results show that the impact of SWC is well captured by NDVI for most biomes. Nevertheless, occasionally GPP was modelled to be higher than the upper boundary as indicated by the bell‐shaped relationship seen with SWC (Figure 4), and LRF‐GPP was therefore reduced to this upper boundary level.

The average global terrestrial LRF‐GPP value is consistent with previous estimates, for instance by the IPCC report, which provides an estimate of 123 ± 8 Pg C year^−1^ (Beer et al., 2010; Ciais et al., 2013). In a review of multiple GPP models and products (Anav et al., 2015), global annual GPP values ranged from 112 to 169 Pg C, whereas a range of 100 to 150 Pg C is consistent with observations of variations in the oxygen isotope composition of atmospheric CO_2_ (Ciais et al., 1997; Farquhar et al., 1993). The average global GPP estimated by the LRF‐GPP is in the middle of this range of global‐scale GPP, indicating a realistic GPP budget estimate.

Several factors affect the trends in global GPP, including rising atmospheric CO_2_ concentrations, climate change and land‐use and land cover change (Nemani et al., 2003; Piao et al., 2020). GPP trends quantified by Earth observation‐based models are generally smaller than those simulated by dynamic global vegetation models, and this difference has been attributed to the absence of CO_2_ fertilization effects in the observation‐based approaches (Anav et al., 2015; Kolby Smith et al., 2015; Piao et al., 2020). Many studies have indicated increased CO_2_ uptake under elevated CO_2_ (Gifford, 2004; Schimel et al., 2015; Tagesson et al., 2020). On the other hand, analyses of biomass increment in CO_2_ enrichment experiments and tree rings in forests indicate that the fertilization effect disappears at large spatial scales, due to the overriding effects of other constraints, such as extreme weather events, nutrients and SWC (Hararuk et al., 2019; Terrer et al., 2019). We note that, in our analysis, we do not see any relationships between atmospheric CO_2_ concentrations and GPP for any biome, except against the upper boundary for evergreen needleleaf forest (SI subsection S3). Our analysis was based on FLUXNET data from 1992 to 2015, that is, over a substantial part of the period during which the trend was estimated. The trend in LRF‐GPP followed the NDVI pattern of GIMMS3g closely (Piao et al., 2020), and it can be argued that the effect of CO_2_ fertilization may already be captured in NDVI, through an increase in greenness.

There has been a pause in the growth rate of atmospheric CO_2_ during the hiatus period of stagnating global temperatures (1998–2013), which has been attributed to increased CO_2_ uptake caused by the CO_2_ fertilization effects (Keenan et al., 2016; Tagesson et al., 2020). Other Earth observation‐based GPP products showed either no trends (falsifying this hypothesis) or a continuous increase throughout this hiatus period (supporting this hypothesis) (Figure 7). Over the study period 1982–2015, there was no overall trend in precipitation (Adler et al., 2017), but there were trends in solar irradiance at the Earth surface (Wild, 2016) and in atmospheric CO_2_ concentrations (Keeling et al., 2005), whereas air temperature increased during the eighties and nineties and stagnated during the hiatus period (Keenan et al., 2016). The time series of the LRF‐GPP budget also showed particularly strong trends during the eighties and nineties, and a stagnation 2000–2015 (Figure 7g), indicating that trends in the LRF‐GPP may be mainly driven by temperature rather than precipitation, irradiance or atmospheric CO_2_ concentrations. We note that the trend of the global terrestrial carbon sink as estimated by the global carbon project is also larger during the eighties and nineties than during the period after 2000 (Le Quéré et al., 2018), and state‐of‐the‐art Vegetation Optical Depths based on the microwave Ku band (VODCA) trends are also substantially more positive for 1987–2016 than for 2002–2017 (Moesinger et al., 2020). The LRF‐GPP budgets thereby support the studies claiming that the pause in the growth rate of atmospheric CO_2_ is caused by reduced respiration or land‐use emission rates, rather than an enhanced CO_2_ uptake (Ballantyne et al., 2017; Keenan et al., 2016; Piao et al., 2018).

Over the last decades, dynamic global vegetation models have been increasingly used to quantify GPP at various spatial and temporal scales (Prentice et al., 2007). In general, these models are based on the Farquhar leaf photosynthesis model (Farquhar et al., 1980). The Farquhar model is particularly sensitive to uncertainty in the photosynthetic capacity (Zhang et al., 2014). We, and several previous studies, have shown that both photosynthetic capacity and the light response of vegetation have substantial spatiotemporal variability, both within and between biomes (Eamus et al., 2013; Ma et al., 2014; Madani et al., 2014; Tagesson et al., 2017; Tagesson, Fensholt, Huber, et al., 2015). Yet, most models apply constant values based on land cover classes or plant functional types (Garbulsky et al., 2010). This study shows that Earth observation data can be used to produce spatially explicit estimates of such ecosystem‐level physiological variables, which can improve our ability to simulate GPP. Spatially explicit estimates of GPP based on Earth observation at high temporal and spatial resolution require a modelling approach taking the GPP saturation at high PAR levels into account. Improving the spatial and temporal resolution in the Earth observation‐based GPP is essential for global and regional‐scale studies, increasing our knowledge regarding relationships of the Earth system to climatic change and anthropogenic forcing.

## Supporting information

Supplementary Material

## Data Availability

The LRF‐modelled GPP data derived in this study are available at annual and daily temporal resolutions at: https://doi.org/10.17894/ucph.b2d7ebfb‐c69c‐4c97‐bee7‐562edde5ce66. The GIMMS NDVI3g data are available upon request from Pinzon and Tucker (2014). The other available datasets were: MCD12C1 land cover data (https://lpdaac.usgs.gov/products/mcd12c1v006/); the ERA‐Interim meteorological data (http://apps.ecmwf.int/datasets/data/interim‐full‐daily/levtype=sfc/); the FLUXNET2015 dataset (https://fluxnet.org/data/fluxnet2015‐dataset/); the MOD17, SMAP and Koly‐Smith GPP products (https://www.ntsg.umt.edu/project/default.php); VPM‐GPP (https://doi.org/10.1038/sdata.2017.165); P‐model GPP (https://zenodo.org/record/1423484.X41NszZ7laQ); FLUXCOM GPP (https://www.fluxcom.org/); the CSIF dataset (https://doi.org/10.6084/m9.figshare.6387494); and the GPPDI NPP data (https://daac.ornl.gov/NPP/guides/NPP_GPPDI.html).
