# Supplementary material for: A physiology‐based Earth observation model indicates stagnation in the global gross primary production during recent decades
Source: Glob Chang Biol. 2020 Dec 6;27(4):836–54. doi: 10.1111/gcb.15424 (PMC7898396; doi:10.1111/gcb.15424)
Supplement: Supplementary file 1 — Supplementary Material [file GCB-27-836-s001.pdf]

Supplementary information:

A physiology-based Earth observation model indicate stagnation in the global gross primary production during recent decades

Torbern Tagesson[1,2*], Feng Tian[1,3], Guy Schurgers[2], Stephanie Horion[2], Robert Scholes[4], Anders Ahlström[1,5], Jonas Ardö[1], Alvaro Moreno[6,7], Nima Madani[8], Stefan Olin[1], Rasmus Fensholt[2]

[1]Department of Physical Geography and Ecosystem Science, Lund University, Sölvegatan 12, SE-223 62 Lund, Sweden

[2]Department of Geosciences and Natural Resource Management, University of Copenhagen, Øster Voldgade 10, DK-1350 Copenhagen, Denmark

[3]School of Remote Sensing and Information Engineering, Wuhan University, Wuhan 430079, China

[4]Global Change Institute, University of the Witwatersrand, Johannesburg 2050, South Africa

[5]Center for Middle Eastern Studies, Lund University, Box 201, SE-221 00 Lund, Sweden.

[6]Image Processing Laboratory (IPL), Universitat de València, C/ Catedrático José Beltrán, 2, 46980 Paterna, València, Spain

[7]Numerical Terradynamic Simulation Group, W.A. Franke College of Forestry & Conservation, University of Montana, Missoula, MT, 59812 USA.

[8]Jet Propulsion Laboratory, 4800 Oak Grove Drive, Pasadena, CA 91109, USA.

S1 FLUXNET2015 sites included in the study

Table S1. List of FLUXNET2015 sites included in this study. The International Geosphere Biosphere Programme (IGBP) classes are: evergreen needleleaf forest (1), evergreen broadleaf forest (2), deciduous needleaf forest (3), deciduous broadleaf forest (4), mixed forest (5), closed shrublands (6), open shrublands (7), woody savannas (8), savannas (9) grasslands (10), permanent wetlands (11), and croplands (12). The biomes are: evergreen needleleaf forest (ENF), evergreen broadleaf forest (EBF), deciduous needleaf forest (DNF), deciduous broadleaf forest (DBF), mixed forest (MIX), savanna/shrublands (SAS), grasslands (GRA), croplands (CRO).

Site name	SITE ID	Latitude	Longitude	IGBP-class	Biome	Site name	SITE ID	Latitude	Longitude	IGBP-class	Biome
AT-Neu	3	47.117	11.318	10	GRA	DK-ZaH	92	74.473	-20.550	10	GRA
AU-Ade	4	-13.077	131.118	8	SAV	ES-Amo	93	36.834	-2.252	6	OSH
AU-Cpr	6	-34.002	140.589	9	SAV	ES-LJu	95	36.927	-2.752	6	OSH
AU-DaS	9	-14.159	131.388	8	SAV	FI-Let	99	60.642	23.960	1	ENF
AU-Dry	10	-15.259	132.371	8	SAV	FR-Gri	103	48.844	1.952	12	CRO
AU-GWW	14	-30.191	120.654	9	SAV	GH-Ank	107	5.269	-2.694	2	EBF
AU-RDF	17	-14.564	132.478	8	SAV	IT-CA2	110	42.377	12.026	12	CRO
AU-Rob	19	-17.118	145.630	2	EBF	MY-PSO	129	2.973	102.306	2	EBF
AU-Stp	20	-17.151	133.350	10	GRA	NO-Adv	132	78.186	15.923	11	WET
AU-TTE	21	-22.287	133.640	6	OSH	RU-Ha1	139	54.725	90.002	10	GRA
AU-Tum	22	-35.657	148.152	2	EBF	RU-Sam	140	72.373	126.498	10	GRA
AU-Wac	23	-37.426	145.188	2	EBF	RU-SkP	141	62.255	129.168	3	DNF
AU-Ync	26	-34.989	146.291	10	GRA	RU-Tks	142	71.594	128.888	10	GRA
BE-Bra	27	51.309	4.521	5	MF	SE-St1	145	68.354	19.050	11	WET
BE-Lon	28	50.552	4.746	12	CRO	US-AR2	148	36.636	-99.598	10	GRA
BE-Vie	29	50.305	5.998	5	MF	US-ARM	151	36.606	-97.489	12	CRO
BR-Sa3	31	-3.018	-54.971	2	EBF	US-Atq	152	70.470	-157.409	11	WET
CA-Gro	32	48.217	-82.156	5	MF	US-Blo	153	38.895	-120.633	1	ENF
CA-Man	33	55.880	-98.481	1	ENF	US-Cop	154	38.090	-109.390	10	GRA
CA-NS1	34	55.879	-98.484	1	ENF	US-CRT	155	41.629	-83.347	12	CRO
CA-NS2	35	55.906	-98.525	1	ENF	US-Lin	164	36.357	-119.842	12	CRO
CA-NS3	36	55.912	-98.382	1	ENF	US-LWW	166	34.960	-97.979	10	GRA

CA-NS4	37	55.912	-98.382	1	ENF	US-Me1	167	44.579	-121.500	1	ENF
CA-NS5	38	55.863	-98.485	1	ENF	US-Me2	168	44.452	-121.557	1	ENF
CA-Qfo	43	49.693	-74.342	1	ENF	US-Me3	169	44.315	-121.608	1	ENF
CA-SF2	45	54.254	-105.878	1	ENF	US-Me4	170	44.499	-121.622	1	ENF
CH-Oe2	58	47.286	7.734	12	CRO	US-Me5	171	44.437	-121.567	1	ENF
CN-Cha	59	42.403	128.096	5	MF	US-Me6	172	44.323	-121.608	1	ENF
CN-Din	62	23.173	112.536	2	EBF	US-MMS	173	39.323	-86.413	4	DBF
CN-Du2	63	42.047	116.284	10	GRA	US-Ne1	175	41.165	-96.477	12	CRO
CN-Du3	64	42.055	116.281	10	GRA	US-Ne2	176	41.165	-96.470	12	CRO
CN-Ha2	65	37.609	101.327	11	WET	US-Ne3	177	41.180	-96.440	12	CRO
CN-HaM	66	37.370	101.180	10	GRA	US-PFa	181	45.946	-90.272	5	MF
CN-Sw2	68	41.790	111.897	10	GRA	US-SRC	183	31.908	-110.840	6	OSH
CZ-BK1	69	49.502	18.537	1	ENF	US-SRM	185	31.821	-110.866	8	SAV
DE-Geb	73	51.100	10.914	12	CRO	US-Syv	187	46.242	-89.348	5	MF
DE-Obe	79	50.784	13.720	1	ENF	US-Ton	188	38.432	-120.966	8	SAV
DE-Tha	85	50.964	13.567	1	ENF	US-Whs	198	31.744	-110.052	6	OSH
DK-Fou	88	56.484	9.587	12	CRO	ZA-Kru	211	-25.020	31.497	8	SAV
DK-ZaF	91	74.481	-20.555	11	WET						

S2 Order of the environmental constraints on GPP

To determine in which order the constraining environmental variables (T_{air}, CO_2, SWC, and VPD) were to be introduced in the model for the different biomes, we used a regression tree analysis. It is a robust statistical tool to analyse complex, nonlinear relationships and interactions between a single response variable and several explanatory variables (De'ath & Fabricius, 2000). The data set was repeatedly split into more and more homogeneous subgroups, each categorized by values of both the dependent and the independent variables. Splitting continues until a tree is created, which is then pruned back to a proper size according a cross validation procedure. In each analysis, we separated the data into ten subgroups of approximately equal size. Each subgroup was left out once, and ten trees were created with the remaining nine subgroups, which were evaluated against the left-out subgroup. The error was summed for all ten trees and for each of the different tree sizes. The smallest tree with the minimum error was selected. The cross-validation was repeated 100 times and the most common tree size was used in the final analysis. We repeated the analysis allowing various number of days in each subgroup (bin size) for further splitting the tree, and then used the bin size generating the lowest RMSE in the final analysis. The analysis picks out the environmental variables which makes it easiest to place GPP within a certain easily defined range. It was therefore used for deciding the order in which the environmental constraining variables were applied in the GPP model for the different biomes (Table S2).

Table S2. Statistics for the regression tree analysis studying relationships between seasonal dynamics in the gross primary production and the explanatory variables for the various biomes. The R^2 is the coefficient of determination and RMSE is the root-mean-square-error for the regression trees. T_{air} is air temperature at 2 m height (°C), NDVI is the normalized difference vegetation index, PAR is daily mean photosynthetically active radiation (W m^{-2}), SWC is soil water content at 0.07 m soil depth (% volumetric water content), and VPD is vapour pressure deficit (kPa). The values in brackets are relative predictor importance. The bin size is minimum data size for each subgroup, and pruning level is the number of splits of the regression tree.

Explanatory variables:	1	2	3	4	5	6	R^2	RMSE	Bin size	Pruning level
Evergreen needleleaf forest (1)	T_{air} (0.60)	NDVI (0.20)	PAR (0.09)	CO_2 (0.07)	SWC (0.02)	VPD (0.01)	0.90	0.95	4	400
Evergreen broadleaf forest (2)	NDVI (0.28)	T_{air} (0.26)	PAR (0.23)	CO_2 (0.14)	SWC (0.06)	VPD (0.04)	0.72	1.61	2	141
Deciduous needleleaf forest (3)	NDVI (0.66)	PAR (0.11)	SWC (0.09)	CO_2 (0.07)	T_{air} (0.05)	VPD (0.03)	0.62	0.48	1	16
Deciduous broadleaf forest (4)	T_{air} (0.67)	NDVI (0.13)	PAR (0.12)	CO_2 (0.03)	SWC (0.03)	VPD (0.02)	0.95	1.08	5	159
Mixed forest (5)	T_{air} (0.64)	NDVI (0.16)	PAR (0.13)	SWC (0.04)	CO_2 (0.02)	VPD (0.01)	0.89	1.18	12	422
Savanna/shrublands (6-9)	NDVI (0.73)	SWC (0.08)	PAR (0.08)	T_{air} (0.06)	CO_2 (0.03)	VPD (0.02)	0.86	0.45	1	1910
Grasslands/wetlands (10,11)	NDVI (0.66)	SWC (0.14)	PAR (0.12)	CO_2 (0.03)	T_{air} (0.03)	VPD (0.02)	0.93	0.79	3	610
Croplands (12)	NDVI (0.77)	T_{air} (0.10)	PAR (0.07)	CO_2 (0.04)	SWC (0.01)	VPD (0.01)	0.95	1.35	3	981

S3 Impact of meteorological and hydrological stress on GPP

S3.1 Evergreen needleleaf forest

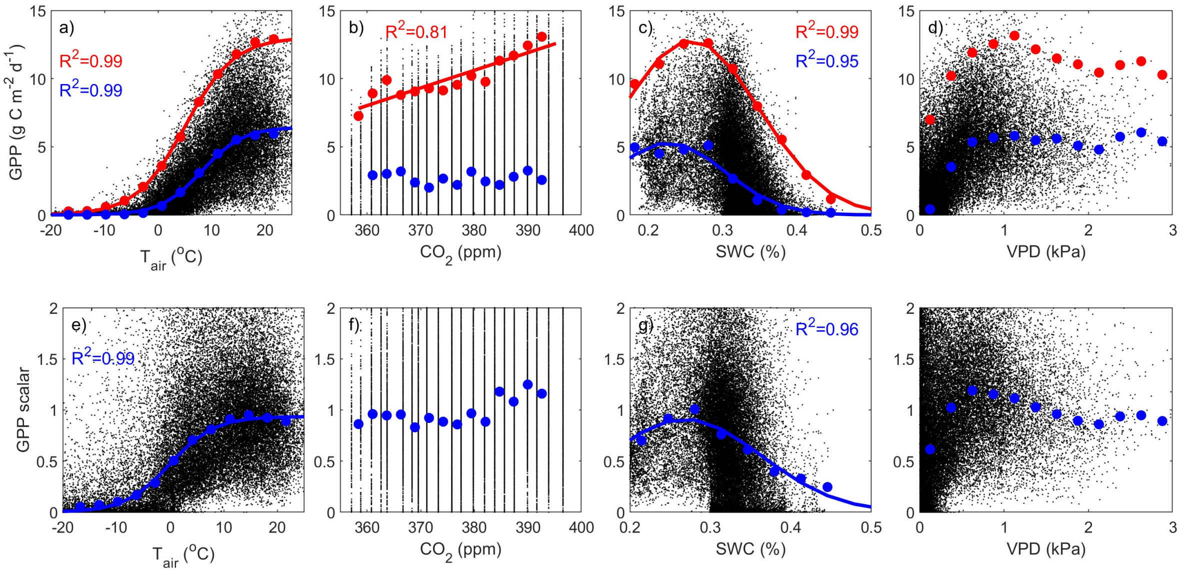

Figure S1 Relationships between GPP variability and environmental variables for evergreen needleleaf forest. a-d) Relationships between direct measures of GPP and the environmental variables, and e-h) relationship between the ratio between modelled and measured GPP (the GPP scalar) and the environmental variables. The environmental variables are: air temperature at 2 m height (T_{air}; °C), atmospheric CO_2 concentrations, soil water content at 0.0-0.07 m soil depth (SWC %volumetric water content), and vapour pressure deficit (VPD; kPa). The variables follow the order of introduction into the model based on the regression tree analysis (Table S2). Included are the impact on the upper threshold (98th percentile; red) and the median (blue).

S3.2 Evergreen broadleaf forest

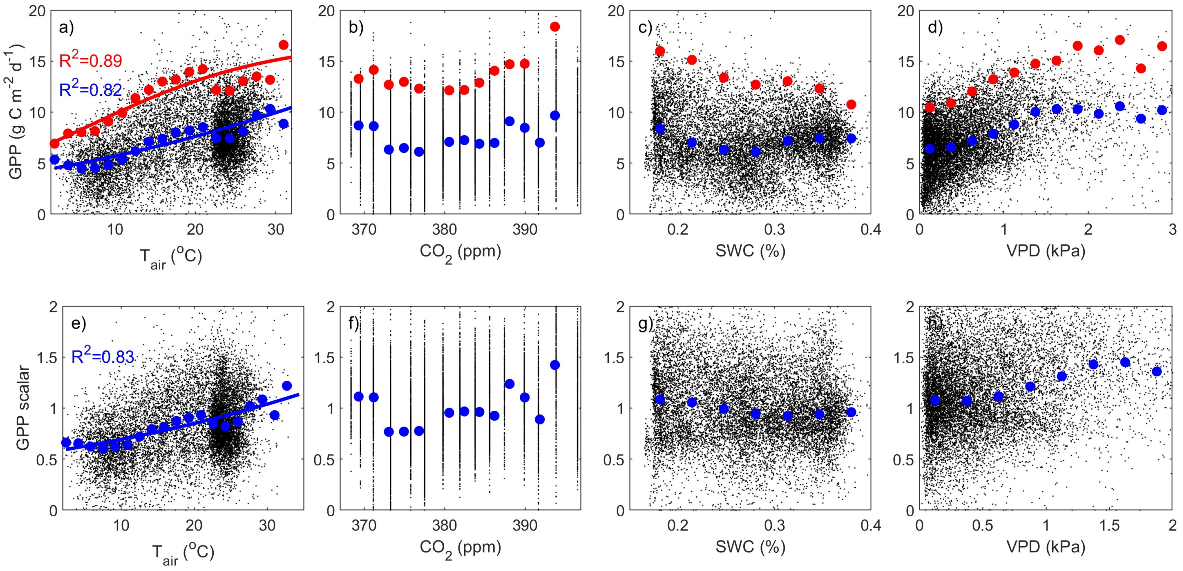

Figure S2 Relationships between GPP variability and environmental variables for evergreen broadleaf forest. a-d) Relationships between direct measures of GPP and the environmental variables, and e-h) relationship between the ratio between modelled and measured GPP (the GPP scalar) and the environmental variables. The environmental variables are: air temperature at 2 m height (T_{air}; °C), atmospheric CO_2 concentrations, soil water content at 0.0-0.07 m soil depth (SWC %volumetric water content), and vapour pressure deficit (VPD; kPa). The variables follow the order of introduction into the model based on the regression tree analysis (Table S2). Included are the impact on the upper threshold (98th percentile; red) and the median (blue).

S3.3 Deciduous needleleaf forest

Deciduous needleleaf forest is mainly larch forest located at the northern hemisphere in the Siberian tundra (Fig S1). It is the biome with least data in FLUXNET 2015 data base generating a highly uncertain analysis for this biome.

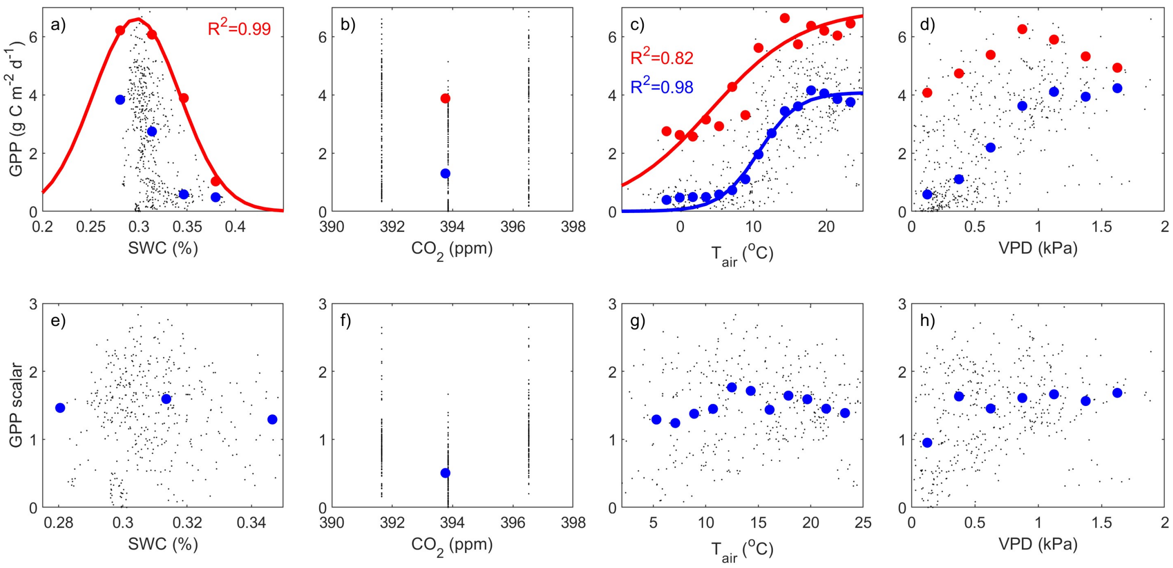

Figure S3 Relationships between GPP variability and environmental variables for deciduous needleleaf forest. a-d) Relationships between direct measures of GPP and the environmental variables, and e-h) relationship between the ratio between modelled and measured GPP (the GPP scalar) and the environmental variables. The environmental variables are: air temperature at 2 m height (T_{air}; °C), atmospheric CO_2 concentrations, soil water content at 0.0-0.07 m soil depth (SWC %volumetric water content), and vapour pressure deficit (VPD; kPa). The variables follow the order of introduction into the model based on the regression tree analysis (Table S2). Included are the impact on the upper threshold (98th percentile; red) and the median (blue).

S3.4 Deciduous broadleaf forest

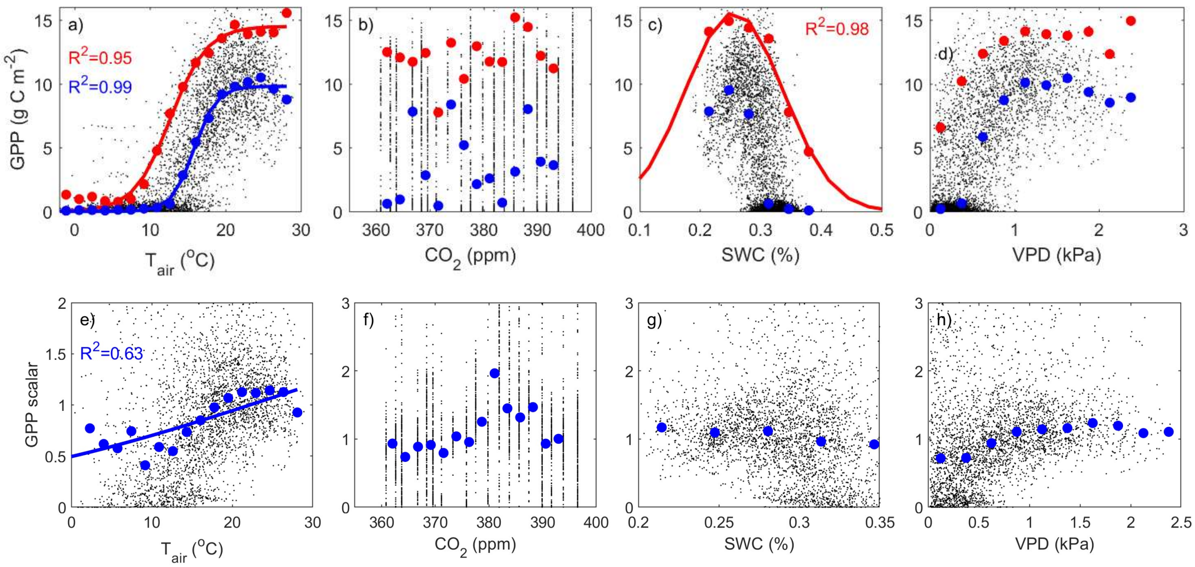

Figure S4 Relationships between GPP variability and environmental variables for deciduous needleleaf forest. a-d) Relationships between direct measures of GPP and the environmental variables, and e-h) relationship between the ratio between modelled and measured GPP (the GPP scalar) and the environmental variables. The environmental variables are: air temperature at 2 m height (T_{air}; °C), atmospheric CO_2 concentrations, soil water content at 0.0-0.07 m soil depth (SWC %volumetric water content), and vapour pressure deficit (VPD; kPa). The variables follow t the order of introduction into the model based on the regression tree analysis (Table S2). Included are the impact on the upper threshold (98th percentile; red) and the median (blue).

S3.5 Mixed forest

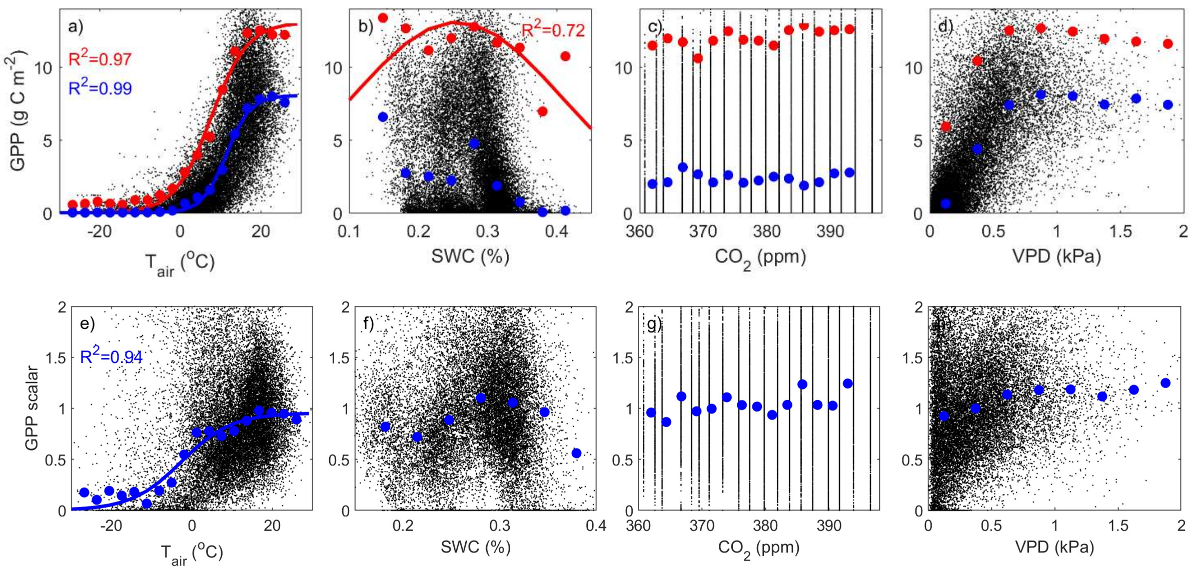

Figure S5 Relationships between GPP variability and environmental variables for mixed forest. a-d) Relationships between direct measures of GPP and the environmental variables, and e-h) relationship between the ratio between modelled and measured GPP (the GPP scalar) and the environmental variables. The environmental variables are: air temperature at 2 m height (T_{air}; °C), atmospheric CO_2 concentrations, soil water content at 0.0-0.07 m soil depth (SWC %volumetric water content), and vapour pressure deficit (VPD; kPa). The variables follow the order of introduction into the model based on the regression tree analysis (Table S2). Included are the impact on the upper threshold (98th percentile; red) and the median (blue).

S3.6 Savanna/shrublands

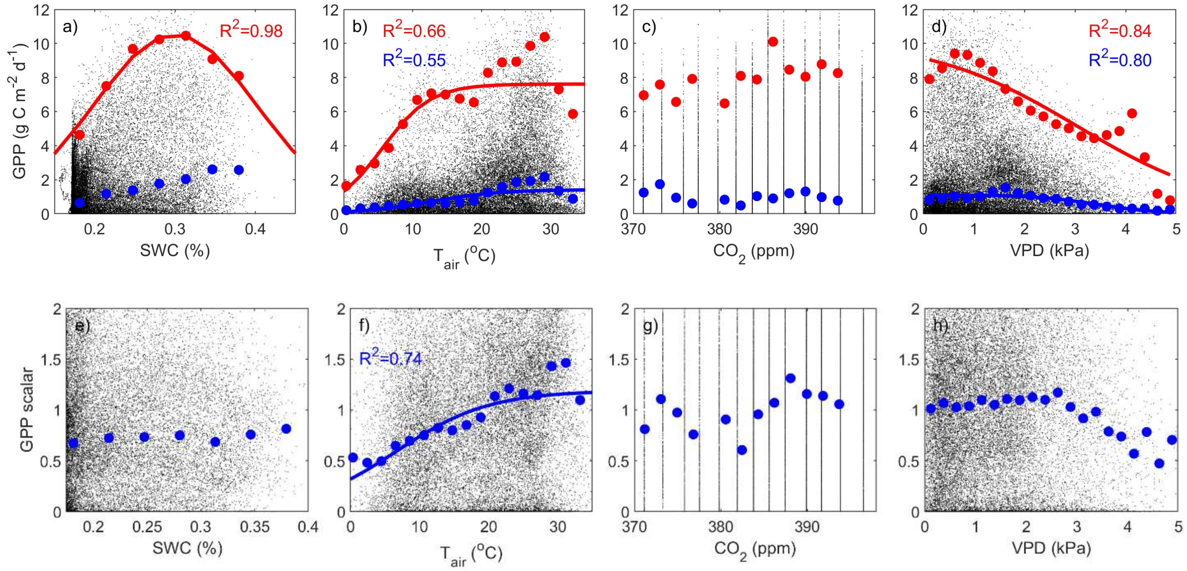

Figure S6 Relationships between GPP variability and environmental variables for savanna/shrublands. a-d) Relationships between direct measures of GPP and the environmental variables, and e-h) relationship between the ratio between modelled and measured GPP (the GPP scalar) and the environmental variables. The environmental variables are: air temperature at 2 m height (T_{air}; °C), atmospheric CO_2 concentrations, soil water content at 0.0-0.07 m soil depth (SWC %volumetric water content), and vapour pressure deficit (VPD; kPa). The variables follow the order of introduction into the model based on the regression tree analysis (Table S2). Included are the impact on the upper threshold (98th percentile; red) and the median (blue).

S3.7 Grasslands

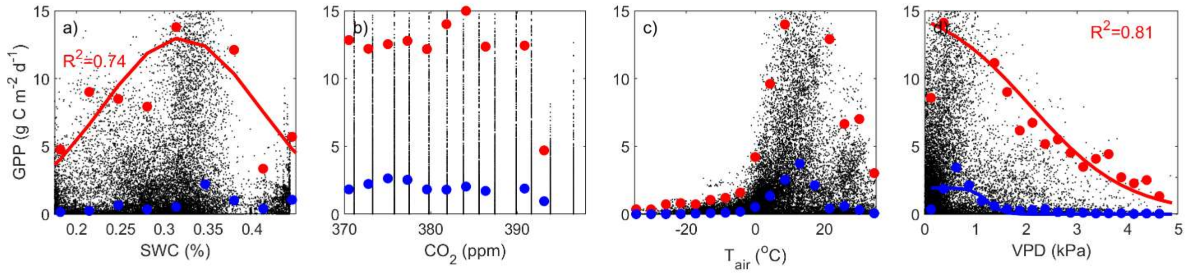

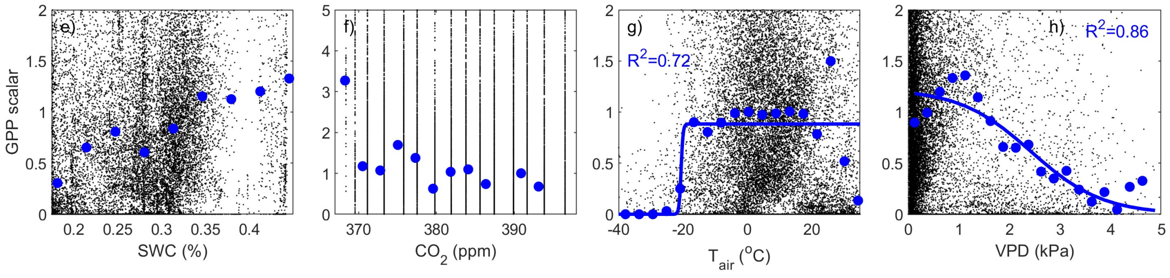

Figure S7 Relationships between GPP variability and environmental variables for grasslands a-d) Relationships between direct measures of GPP and the environmental variables, and e-h) relationship between the ratio between modelled and measured GPP (the GPP scalar) and the environmental variables. The environmental variables are: air temperature at 2 m height (T_{air}; °C), atmospheric CO_2 concentrations, soil water content at 0.0-0.07 m soil depth (SWC %volumetric water content), and vapour pressure deficit (VPD; kPa). The variables follow the order of introduction into the model based on the regression tree analysis (Table S2). Included are the impact on the upper threshold (98th percentile; red) and the median (blue).

S3.8 Croplands

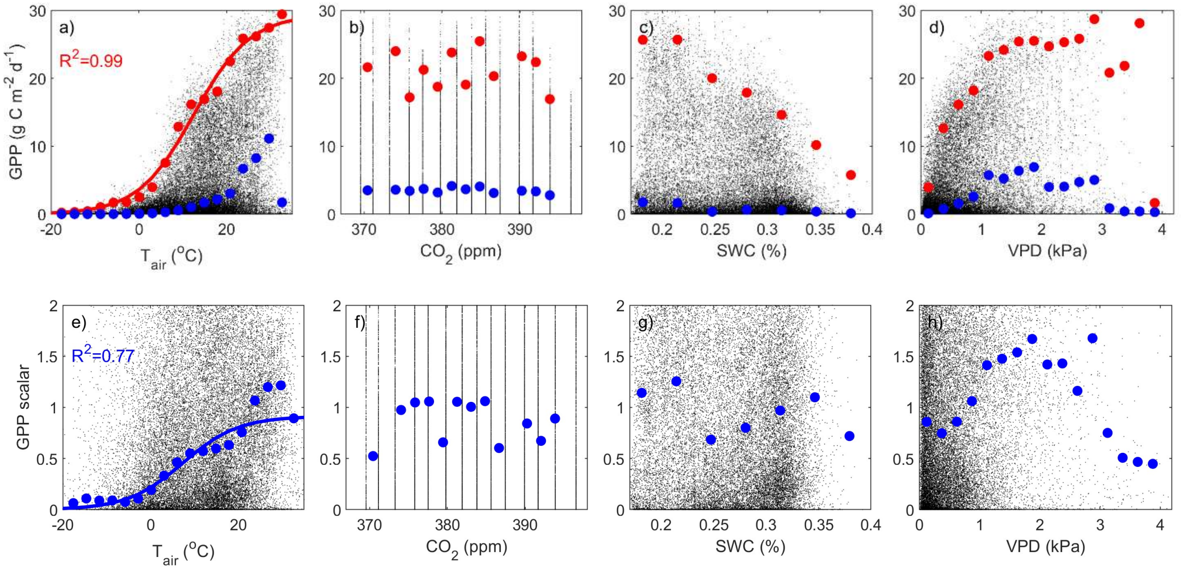

Figure S8 Relationships between GPP variability and environmental variables for grasslands. a-d) Relationships between direct measures of GPP and the environmental variables, and e-h) relationship between the ratio between modelled and measured GPP (the GPP scalar) and the environmental variables. The environmental variables are: air temperature at 2 m height (T_{air}; °C), atmospheric CO_2 concentrations, soil water content at 0.0-0.07 m soil depth (SWC %volumetric water content), and vapour pressure deficit (VPD; kPa). The variables follow the order of introduction into the model based on the regression tree analysis (Table S2). Included are the impact on the upper threshold (98th percentile; red) and the median (blue).

S4 Constraining GPP based on environmental stress

For evergreen needleleaf forest, deciduous broadleaf forest, and mixed forest, GPP was modelled as:

$$\begin{aligned} &\mathrm{GPP} = GPP_{opt} \times S_{Tair} && GPP < T_{SWC} \\ &\mathrm{GPP} = T_{SWC} && GPP > T_{SWC} \end{aligned} \qquad \text{(S1)}$$

Where GPP is the final modelled GPP product, GPP_{opt} is the modelled GPP based on the light response function with NDVI-modelled F_{opt} and α (Eq. 1-3 in main text). S_{Tair} is a scalar constraining GPP at low air temperature modelled using Eq. 4 in main text. However, air temperature can only constrain GPP, never enhance it and as such S_{Tair} larger than one was set to one. The T_{SWC} is the threshold of the upper GPP boundary for a certain soil water condition (SWC) condition, and it was modelled based on Eq. 5 in main text. GPP at a certain SWC can never reach higher values than as estimated by the upper boundary, and modelled GPP that was larger was therefore set to T_{SWC}.

For evergreen broadleaf forest and croplands, there were only strong relationships between GPP and air temperature and GPP for these biomes were therefore modelled as:

$$\mathrm{GPP} = GPP_{opt} \times S_{Tair} \qquad \text{(S2)}$$

Strong relationships between upper threshold of GPP and air temperature and SWC were seen for deciduous needleleaf forest, and GPP for this biome was modelled as:

$$\begin{aligned} &\mathrm{GPP} = GPP_{opt} && GPP < T_{SWC} \\ &\mathrm{GPP} = T_{SWC} && GPP > T_{SWC} \\ &\mathrm{GPP} = GPP_{opt} && GPP < T_{Tair} \\ &\mathrm{GPP} = T_{Tair} && GPP > T_{Tair} \end{aligned} \qquad \text{(S3)}$$

Where T_{Tair} is the threshold of the upper GPP for a certain air temperature, and it was modelled based on Eq. 4 in main text.

For savanna/shrublands and grasslands, we additionally saw strong relationships between the upper boundary of GPP and vapour pressure deficit, and modelled GPP was therefore limited to never reach above this threshold:

$$\begin{aligned} &\mathrm{GPP} = GPP_{opt} \times S_{Tair} && GPP < T_{SWC} \\ &\mathrm{GPP} = T_{SWC} && GPP > T_{SWC} \\ &\mathrm{GPP} = GPP_{opt} \times S_{Tair} && GPP < T_{VPD} \\ &\mathrm{GPP} = T_{VPD} && GPP > T_{VPD} \end{aligned} \qquad \text{(S4)}$$

Where T_{VPD} is the upper GPP threshold for a certain vapour pressure deficit condition, and it was modelled based on Eq. 4 in main text.

S5 Model sensitivity analysis

S5.1 Sensitivity of LRF-GPP to F_{opt} and α variability

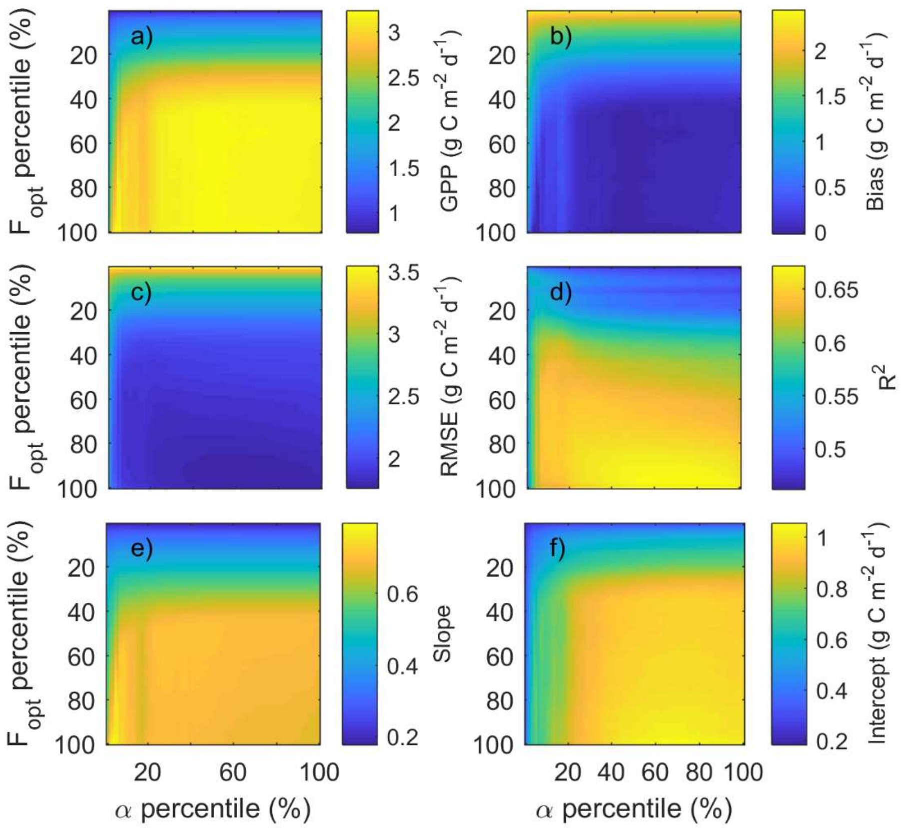

Figure S9 Sensitivity of LRF-GPP to variability in optimized carbon uptake at light saturation (F_{opt}) and quantum efficiency (α) for evergreen needle leaf forest. Impact on a) GPP; b) bias (modelled GPP subtracted from field measured); c) root-mean-square-error (RMSE); d) coefficient of determination (R^2); and e) slope and f) intercept from an ordinary least square linear regression fitted between modeled and field measured GPP. Note that scales differ for the different biomes (Fig. S9-S16).

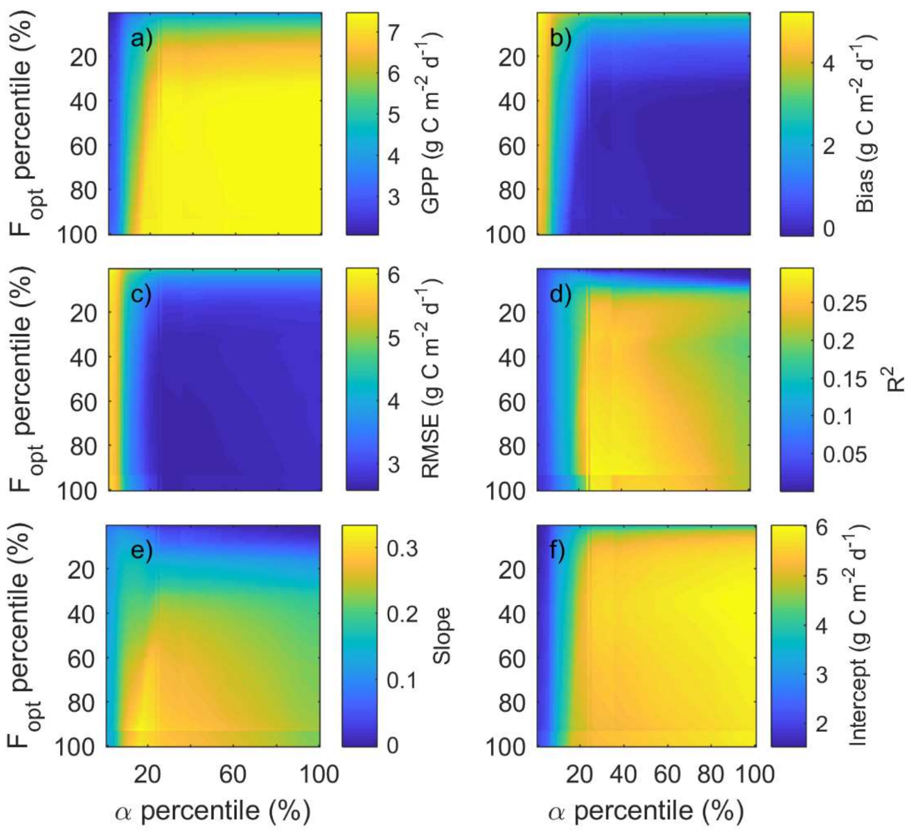

Figure S10 Sensitivity of the LRF-GPP to variability in optimized carbon uptake at light saturation (F_{opt}) and quantum efficiency (α) for evergreen broadleaf forest. Impact on a) GPP; b) bias (modelled GPP subtracted from field measured); c) root-mean-square-error (RMSE); d) coefficient of determination (R^2); and e) slope and f) intercept from an ordinary least square linear regression fitted between modeled and field measured GPP. Note that scales differ for the different biomes (Fig. S9-S16).

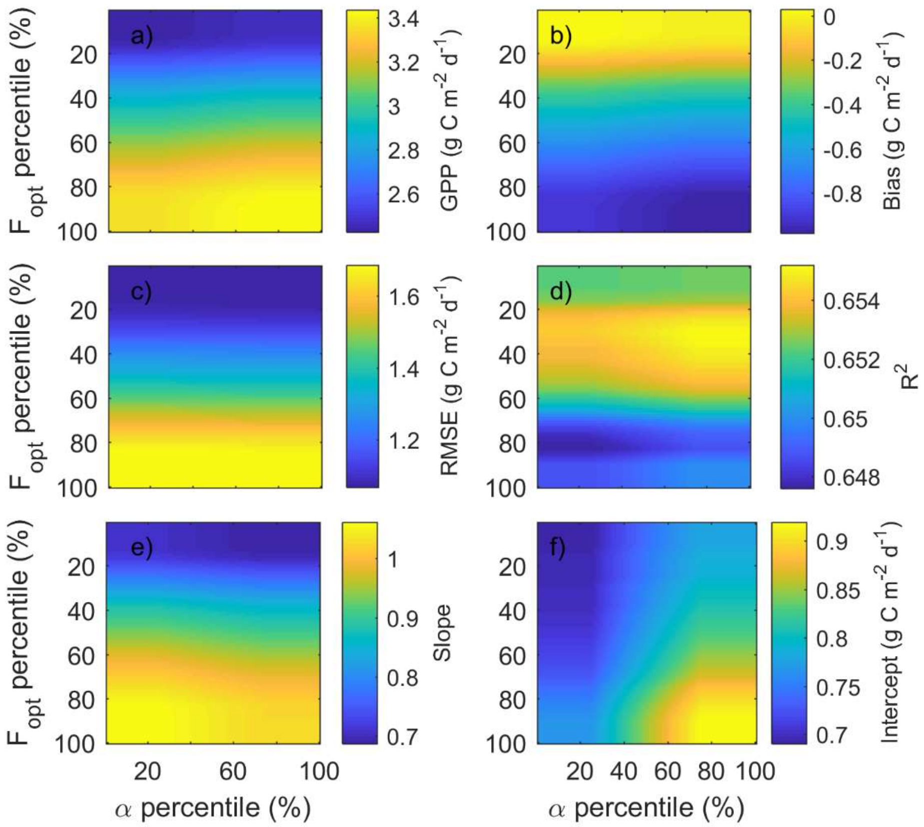

Figure S11 Sensitivity of LRF-GPP to variability in optimized carbon uptake at light saturation (F_{opt}) and quantum efficiency (α) for deciduous needleleaf forest. Impact on a) GPP; b) bias (modelled GPP subtracted from field measured); c) root-mean-square-error (RMSE); d) coefficient of determination (R^2); and e) slope and f) intercept from an ordinary least square linear regression fitted between modeled and field measured GPP. Note that scales differ for the different biomes (Fig. S9-S16).

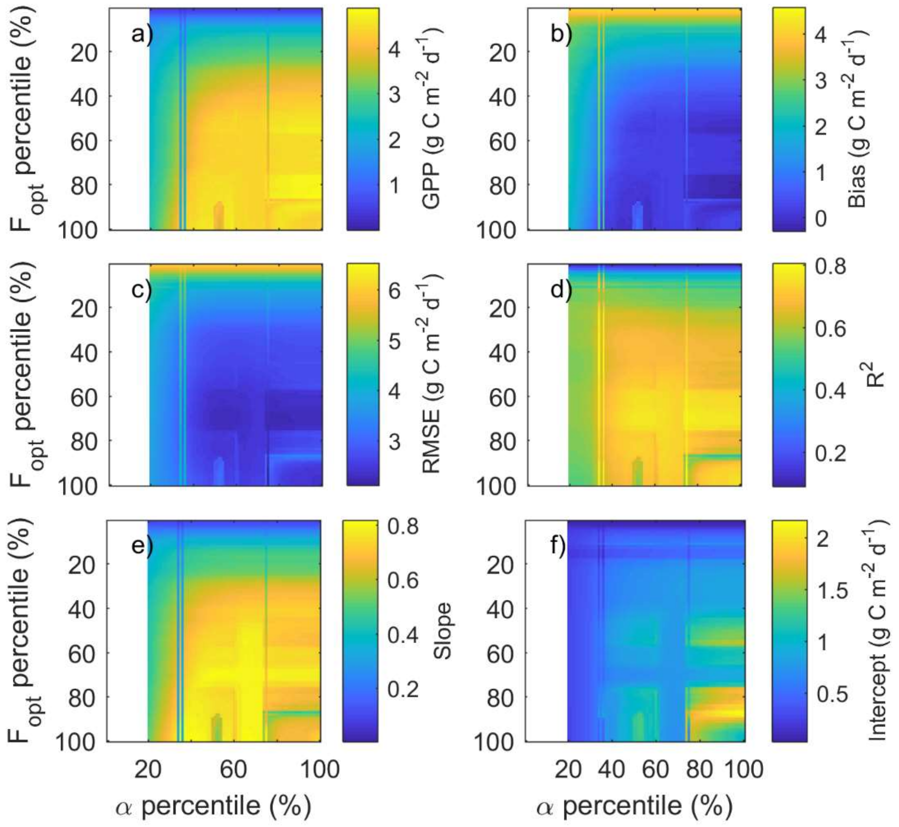

Figure S12 Sensitivity of LRF-GPP to variability in optimized carbon uptake at light saturation (F_{opt}) and quantum efficiency (α) for deciduous broadleaf forest. Impact on a) GPP; b) bias (modelled GPP subtracted from field measured); c) root-mean-square-error (RMSE); d) coefficient of determination (R^2); and e) slope and f) intercept from an ordinary least square linear regression fitted between modeled and field measured GPP. Note that scales differ for the different biomes (Fig. S9-S16).

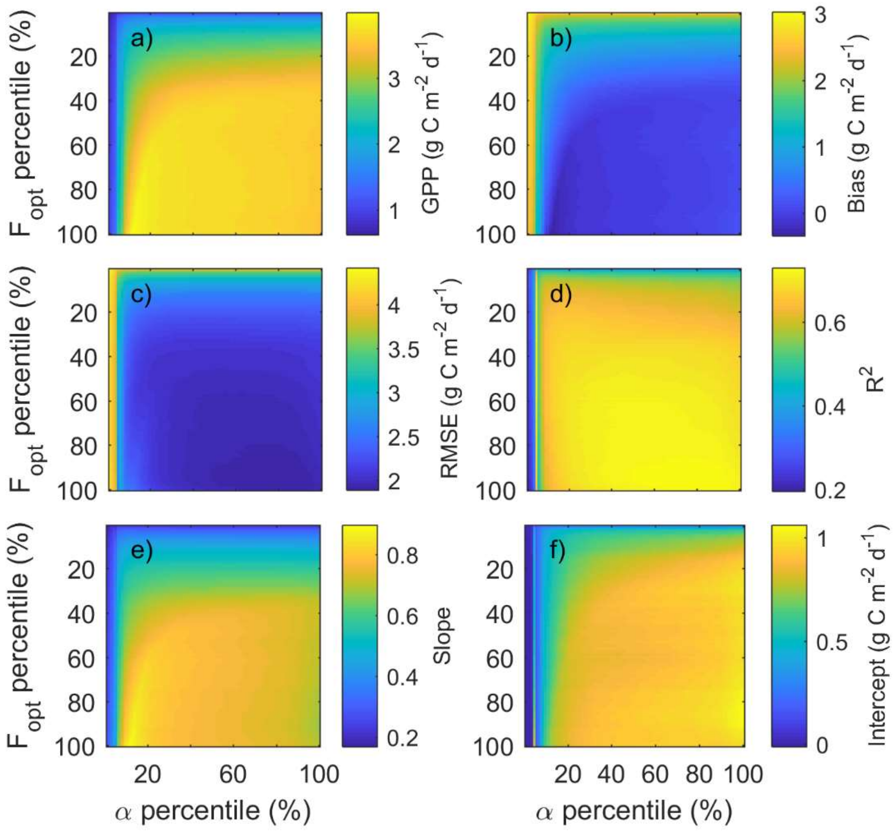

Figure S13 Sensitivity of the LRF-GPP to variability in optimized carbon uptake at light saturation (F_{opt}) and quantum efficiency (α) for mixed forest. Impact on a) GPP; b) bias (modelled GPP subtracted from field measured); c) root-mean-square-error (RMSE); d) coefficient of determination (R^2); and e) slope and f) intercept from an ordinary least square linear regression fitted between modeled and field measured GPP. Note that scales differ for the different biomes (Fig. S9-S16).

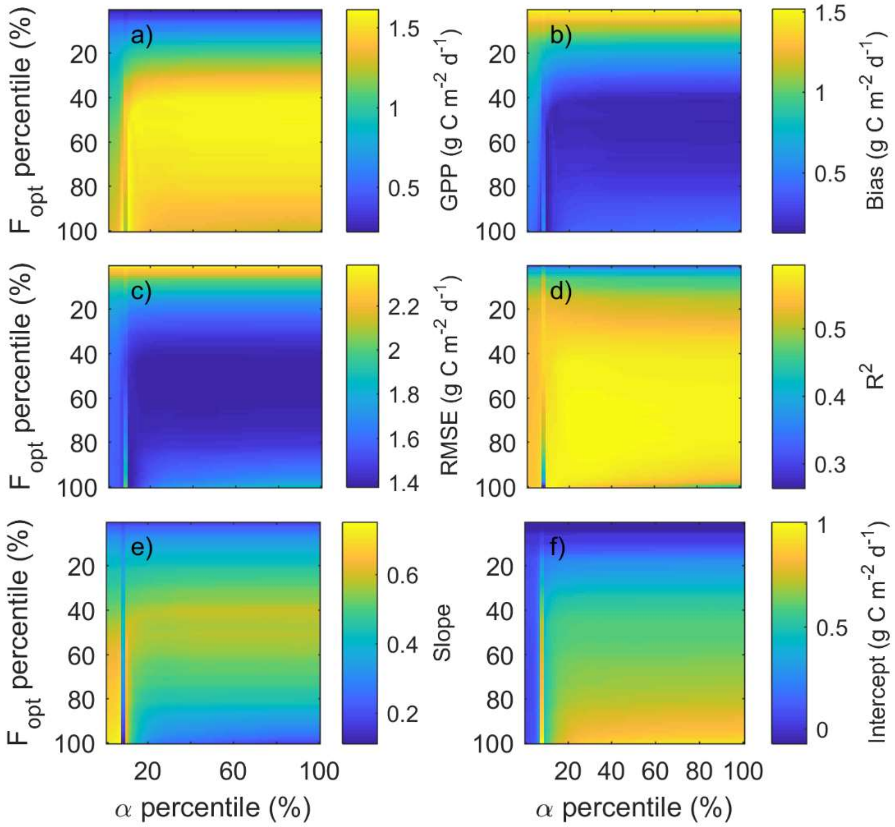

Figure S14 Sensitivity of LRF-GPP to variability in optimized carbon uptake at light saturation (F_{opt}) and quantum efficiency (α) for savanna/shrublands. Impact on a) GPP; b) bias (modelled GPP subtracted from field measured); c) root-mean-square-error (RMSE); d) coefficient of determination (R^2); and e) slope and f) intercept from an ordinary least square linear regression fitted between modeled and field measured GPP. Note that scales differ for the different biomes (Fig. S9-S16).

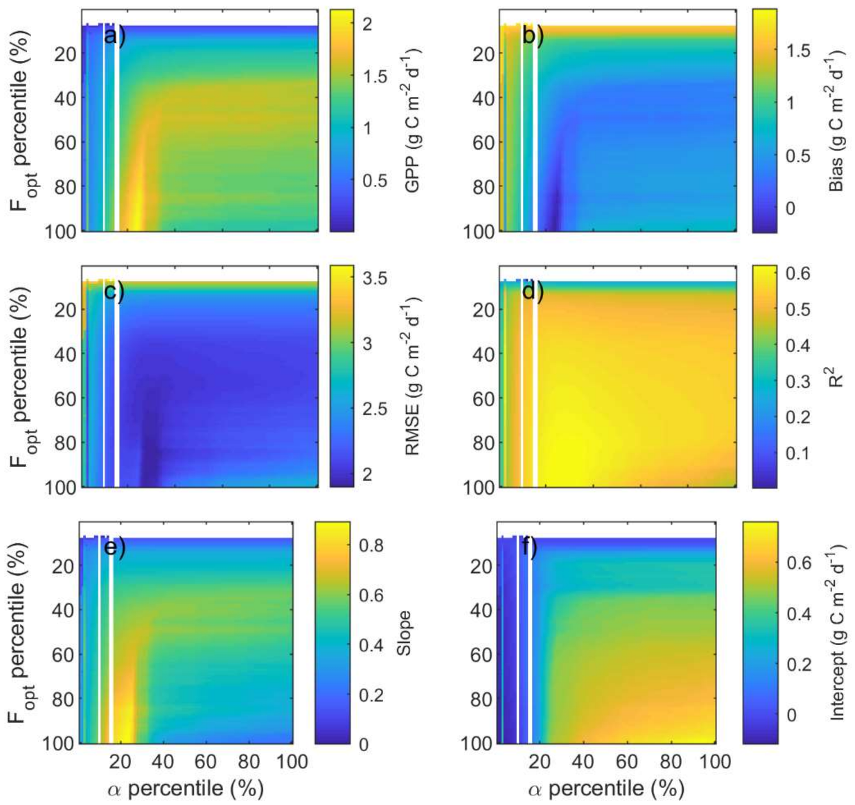

Figure S15 Sensitivity of LRF-GPP to variability in optimized carbon uptake at light saturation (F_{opt}) and quantum efficiency (α) for grasslands. Impact on a) GPP; b) bias (modelled GPP subtracted from field measured); c) root-mean-square-error (RMSE); d) coefficient of determination (R^2); and e) slope and f) intercept from an ordinary least square linear regression fitted between modeled and field measured GPP. Note that scales differ for the different biomes (Fig. S9-S16).

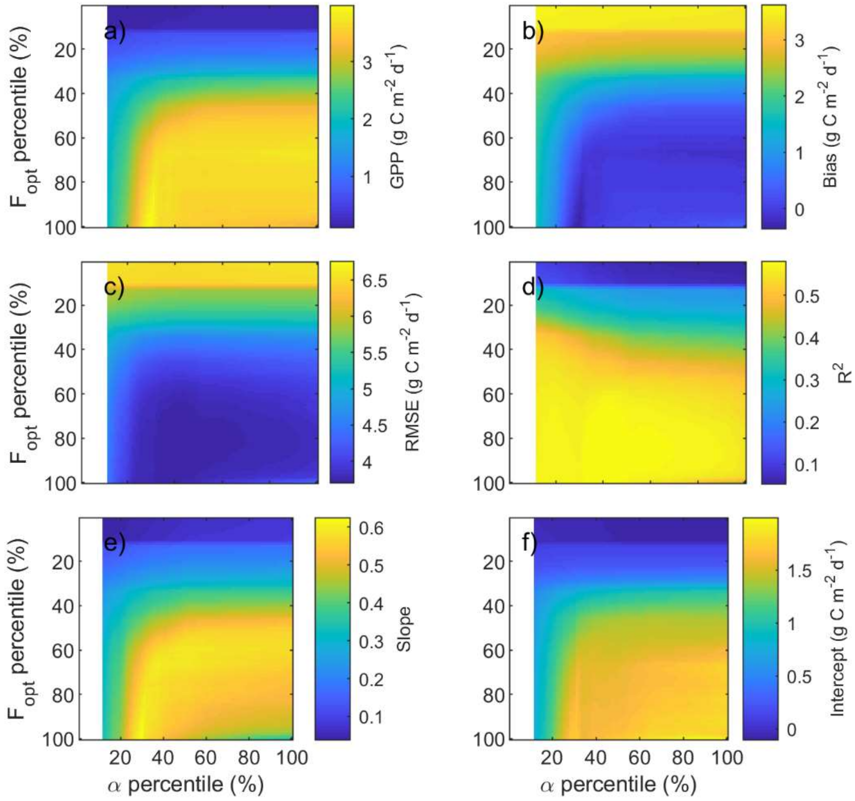

Figure S16 Sensitivity of LRF-GPP to variability in optimized carbon uptake at light saturation (F_{opt}) and quantum efficiency (α) for croplands. Impact on a) GPP; b) bias (modelled GPP subtracted from field measured); c) root-mean-square-error (RMSE); d) coefficient of determination (R^2); and e) slope and f) intercept from an ordinary least square linear regression fitted between modeled and field measured GPP. Note that scales differ for the different biomes (Fig. S9-S16).

S5.2 Sensitivity of the LRF-GPP to S_{Tair} and S_{VPD} variability

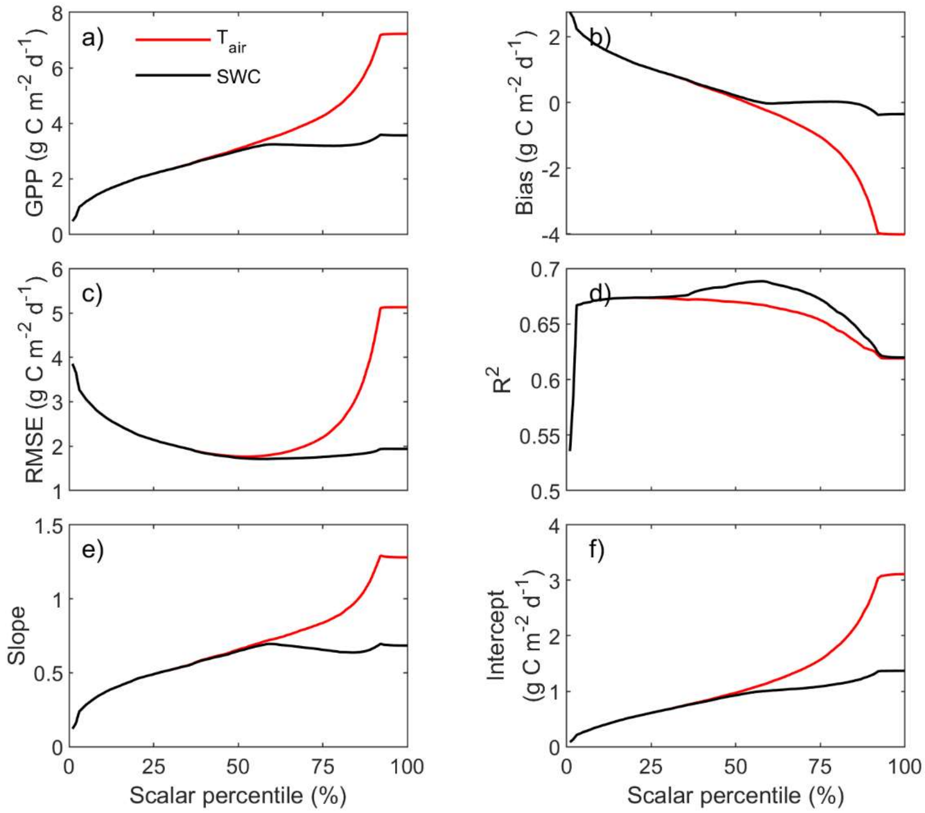

Figure S17 Sensitivity of LRF-GPP to variability in environmental scalars (air temperature (T_{air}); soil water content (SWC)) for evergreen needleleaf forest. The percentiles of each scalar were extracted and the constraining models were fitted. Shown is impact of this percentile fitting on a) GPP; b) bias (modelled GPP subtracted from measured); c) root-mean-square-error (RMSE); d) coefficient of determination (R^2); and e) slope and f) intercept from an ordinary least square linear regression fitted between modeled and measured GPP. Note that scales differ for the different biomes (Fig. S17-S26).

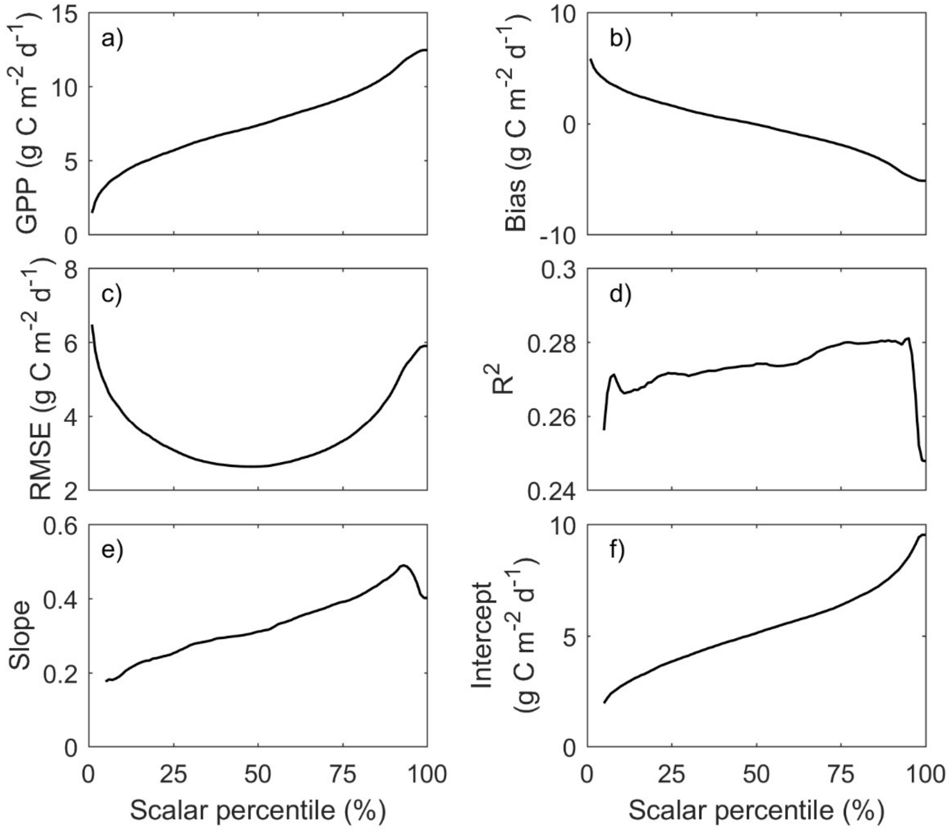

Figure S18 Sensitivity of LRF-GPP to variability in the air temperature scalar for evergreen broadleaf forest. The percentiles of each scalar were extracted and the constraining models were fitted. Shown is impact of this percentile fitting on a) GPP; b) bias (modelled GPP subtracted from measured); c) root-mean-square-error (RMSE); d) coefficient of determination (R^2); and e) slope and f) intercept from an ordinary least square linear regression fitted between modeled and field measured GPP. Note that scales differ for the different biomes (Fig. S17-S26).

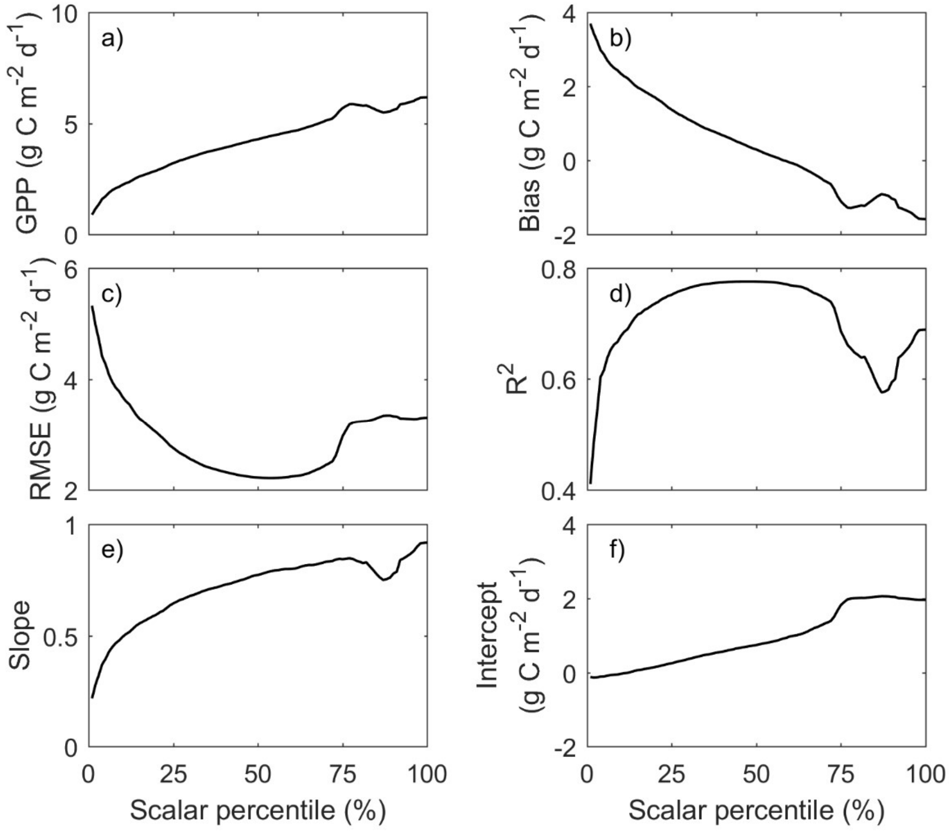

Figure S19 Sensitivity of LRF-GPP to variability in the air temperature scalar for deciduous broadleaf forest. The percentiles of each scalar were extracted and the constraining models were fitted. Shown is impact of this percentile fitting on a) GPP; b) bias (modelled GPP subtracted from field measured); c) root-mean-square-error (RMSE); d) coefficient of determination (R^2); and e) slope and f) intercept from an ordinary least square linear regression fitted between modeled and field measured GPP. Note that scales differ for the different biomes (Fig. S17-S26).

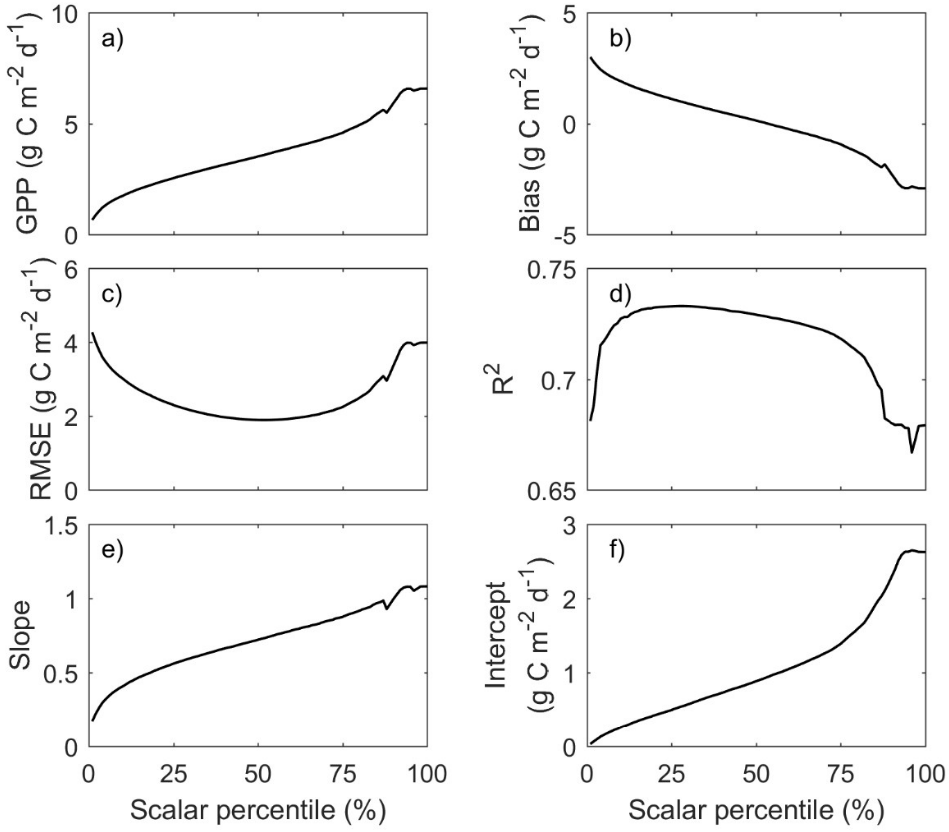

Figure S20 Sensitivity of LRF-GPP to variability in air temperature scalar for mixed forest. The percentiles of each scalar were extracted and the constraining models were fitted. Shown is impact of this percentile fitting on a) GPP; b) bias (modelled GPP subtracted from field measured); c) root-mean-square-error (RMSE); d) coefficient of determination (R^2); and e) slope and f) intercept from an ordinary least square linear regression fitted between modeled and field measured GPP. Note that scales differ for the different biomes (Fig. S17-S26).

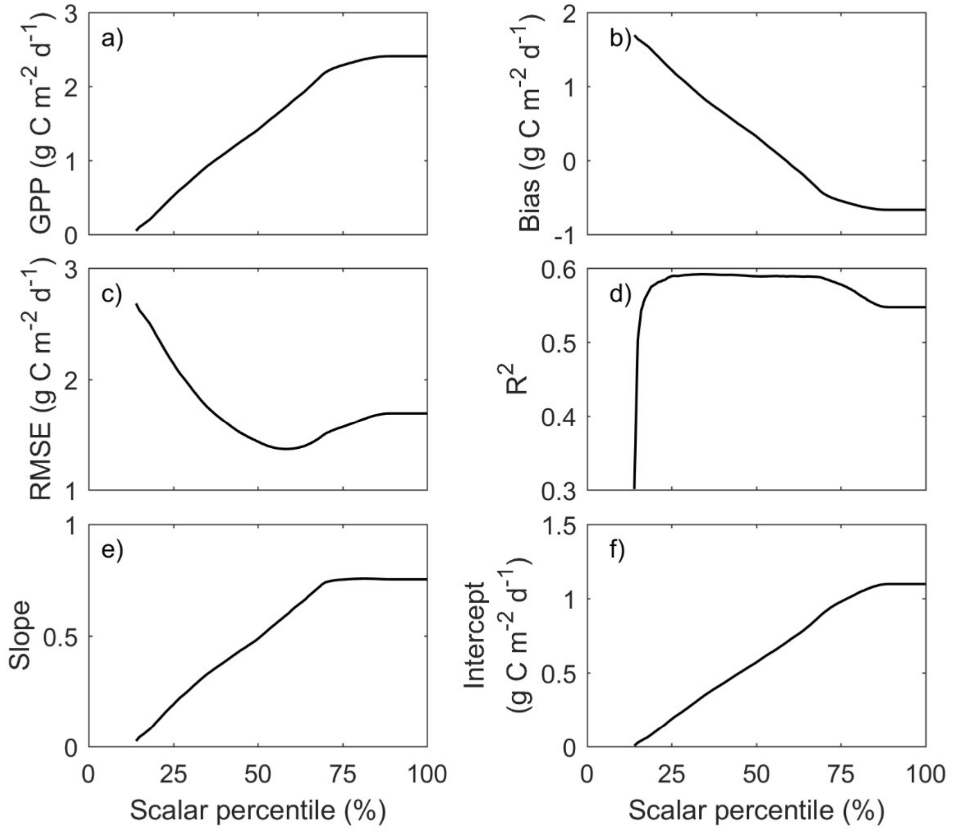

Figure S21 Sensitivity of LRF-GPP to variability in air temperature scalar for savanna/shrublands. The percentiles of each scalar were extracted and the constraining models were fitted. Shown is impact of this percentile fitting on a) GPP; b) bias (modelled GPP subtracted from field measured); c) root-mean-square-error (RMSE); d) coefficient of determination (R^2); and e) slope and f) intercept from an ordinary least square linear regression fitted between modeled and field measured GPP. Note that scales differ for the different biomes (Fig. S17-S26).

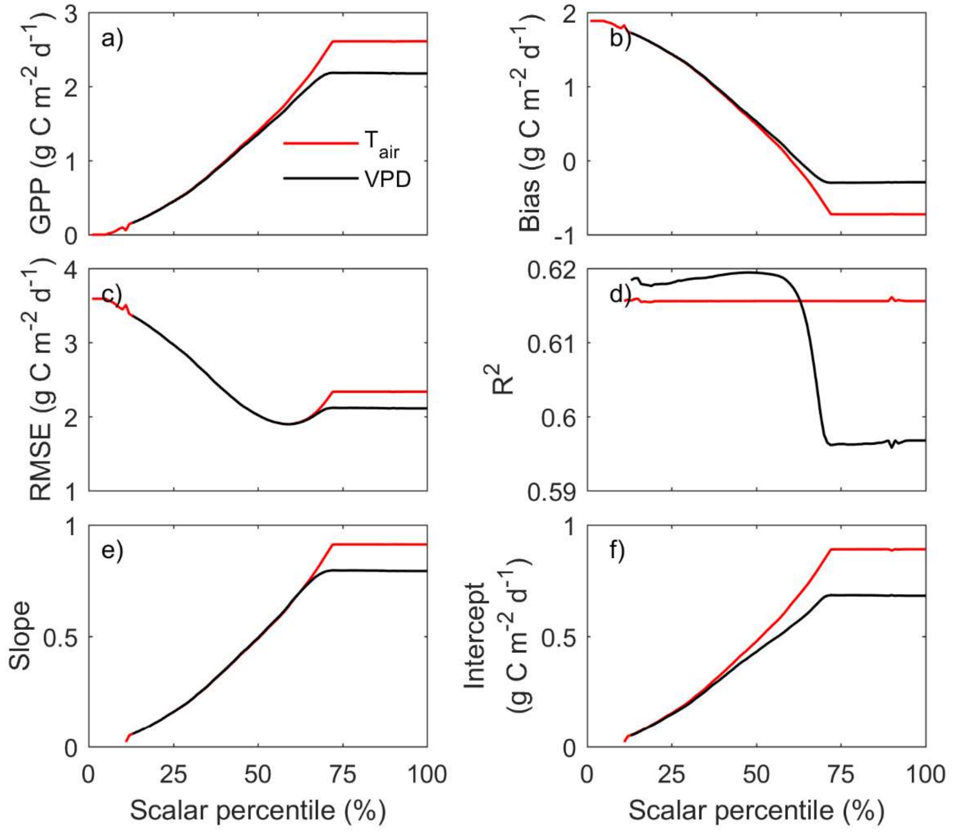

Figure S22 Sensitivity of LRF-GPP to variability in the air temperature (T_{air}) and vapor pressure deficit (VPD) scalars for grasslands. The percentiles of each scalar were extracted and the constraining models were fitted. Shown is impact of this percentile fitting on a) GPP; b) bias (modelled GPP subtracted from field measured); c) root-mean-square-error (RMSE); d) coefficient of determination (R^2); and e) slope and f) intercept from an ordinary least square linear regression fitted between modeled and field measured GPP. Note that scales differ for the different biomes (Fig. S17-S26).

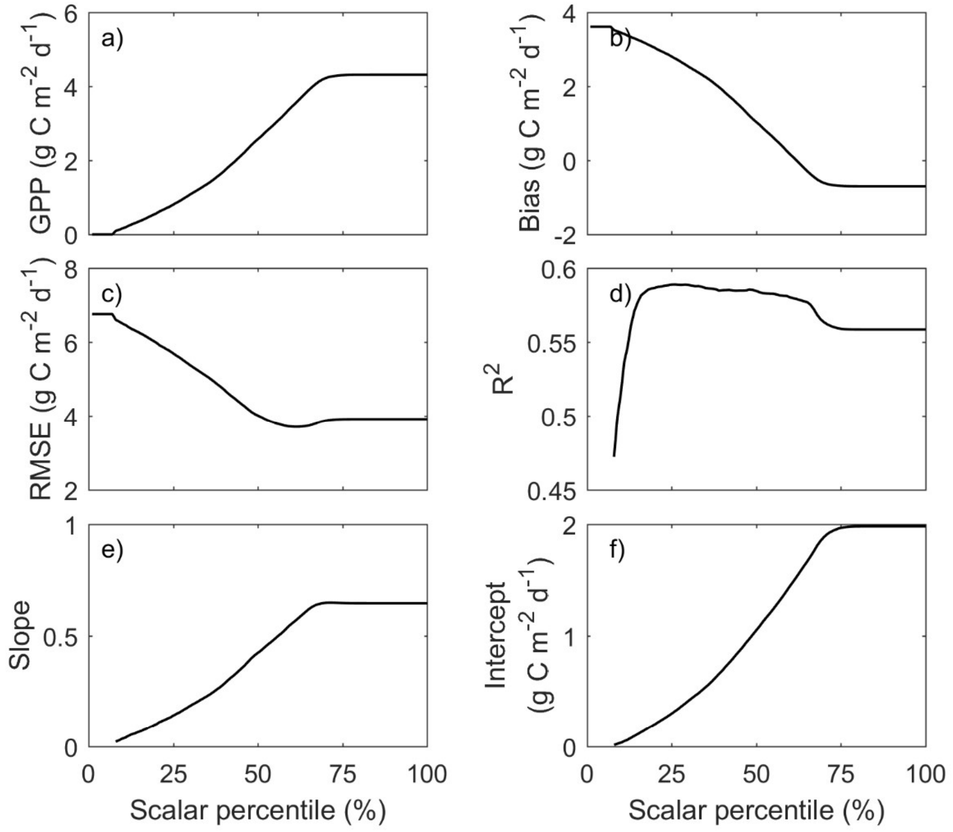

Figure S23 Sensitivity of LRF-GPP to variability in the air temperature scalar for croplands. The percentiles of each scalar were extracted and the constraining models were fitted. Shown is impact of this percentile fitting on a) GPP; b) bias (modelled GPP subtracted from field measured); c) root-mean-square-error (RMSE); d) coefficient of determination (R^2); and e) slope and f) intercept from an ordinary least square linear regression fitted between modeled and field measured GPP. Note that scales differ for the different biomes (Fig. S17-S26).

S6 Model parameters

Table S3. Table of the parameters used in the spatiotemporal extrapolation of photosynthetic capacity and quantum efficiency in the final LRF-GPP model. The parameters are given as average from the 200 bootstrap iterations; ± standard error from the fitting of the logarithmic regression equations; ±1 standard deviation from the fitted parameters of the 200 bootstrap iterations. The numbers in parentheses are the International Geosphere Biosphere Programme (IGBP) land cover classes.

Biome	F_{optmax}	k_{Fopt}	b_{Fopt}	α_{max}	k_{α}	b_{α}
Evergreen Needleleaf Forest (1)	17.16±1.98±3.18	0.07±0.01±0.02	0.57±0.04±0.07	0.26±0.02±0.15	0.10±0.02±0.05	0.54±0.03±0.10
Evergreen Broadleaf Forest (2)	15.82±1.67±141	0.17±0.12±0.21	0.60±0.03±1.88	0.22±0.54±60.3	0.08±0.33±6.56	0.74±0.66±4.00
Deciduous Needleleaf forest (3)	4.49±1.35±1.09	0.10±0.07 ±0.05	0.51±0.10 ±0.01	47.20±3522±1.84	0.09±0.48±0.01	1.36±849.56 ±0.04
Deciduous broadleaf forest (4)	12.02±0.52±2.96	0.89±0.21 ±0.30	0.53±0.00±0.02	67.58±1936±112.0	0.23±0.14±1.87	0.86±12.59±1.79
Mixed forest (5)	15.42±1.60±8.30	0.11±0.03±0.02	0.64±0.03±0.08	1.45±1.93±26.4	0.23±0.01±0.02	0.86±0.35±0.40
Savanna/shrublands (6-9)	10.57±0.90±7.64	0.09±0.01±0.02	0.54±0.03±0.08	0.39±0.53±117.6	0.05±0.02±0.15	0.73±0.47±0.65
Grasslands (10,11)	16.76±1.12±7.21	0.11±0.02±0.03	0.45±0.02±0.15	0.40±0.25±117.7	0.08±0.02±0.05	0.82±0.15±0.41
Croplands (12,16)	27.54±1.83±25.8	0.12±0.02±0.04	0.63±0.02±0.11	0.11±0.02±49.9	0.11±0.04±0.40	0.67±0.05±0.61

Table S4. Table of the scalar parameters used in the final LRF-GPP model. The parameters are given as an average from the 200 bootstrap iterations; ± uncertainty from the fitting of the logarithmic regression equations; ±1 standard deviation from the fitted parameters of the 200 bootstrap iterations. The numbers in parentheses are the International Geosphere Biosphere Programme (IGBP) land cover classes.

Biome		S_T	
Biome	a_{Tmax}	k_T	b_T
Evergreen Needleleaf Forest (1)	0.50±0.01±10.9	0.22±0.03±0.06	2.12±0.65±27.0
Evergreen Broadleaf Forest (2)	2.12±12.2±24.6	0.02±0.04±0.07	67.5±507±144
Deciduous Needleleaf forest (3)	-	-	-
Deciduous broadleaf forest (4)	0.91±0.04±50.3	0.30±0.05 ±0.19	13.4±0.64±346
Mixed forest (5)	0.64±0.02±23.0	0.19±0.02±0.10	2.95±0.03±124.8
Savanna/shrublands (6-9)	0.82±0.08±6.93	0.14±0.06±0.23	4.99±0.03±92.4
Grasslands (10,11)	0.65±0.07±7.21	0.64±1.14±0.03	-24.9±2.43±0.15
Croplands (12,16)	0.97±0.18±207	0.09±0.05±0.11	-2.41±6.19±176

Table S5. Table of the model parameters used for estimating upper thresholds of the final GPP model at certain meteorological and hydrological constraining conditions. In the fitting of the parameters for modelling upper GPP thresholds, all data were used. Therefore, the uncertainty given is from this fitting, and no uncertainty from bootstrap simulations was estimated. The numbers in parentheses are the International Geosphere Biosphere Programme (IGBP) land cover classes.

		T_{SWC}	
Biome	a_{max}	b_{mid}	a_{σ}
Evergreen Needleleaf Forest (1)	12.82±0.24	0.26±0.00	0.008±0.000
Evergreen Broadleaf Forest (2)	-	-	-
Deciduous Needleleaf forest (3)	6.61±0.45	0.30±0.01	0.002±0.001
Deciduous broadleaf forest (4)	15.59±0.80	0.26±0.01	0.007±0.002
Mixed forest (5)	13.08±1.36	0.26±0.02	0.023±0.011
Savanna/Shrublands (6-9)	10.55±0.31	0.30±0.00	0.010±0.001
Grasslands (10,11)	13.00±1.92	0.31±0.02	0.008±0.003
Croplands (12,16)	-	-	-
		T_T	
Biome	a_{Tmax}	k_T	b_T
Evergreen Needleleaf Forest (1)	-	-	-
Evergreen Broadleaf Forest (2)	-	-	-
Deciduous Needleleaf forest (3)	6.95±1.95	0.16±0.23	4.17±0.68
Deciduous broadleaf forest (4)	-	-	-
Mixed forest (5)	-	-	-
Savanna/shrublands (6-9)	-	-	-
Grasslands/wetlands (10,11)	-	-	-
Croplands (12,16)	-	-	-
		T_{VPD}	
Biome	a_{VPDmax}	k_{VPD}	b_{VPD}
Evergreen Needleleaf Forest (1)	-	-	-
Evergreen Broadleaf Forest (2)	-	-	-
Deciduous Needleleaf forest (3)	-	-	-
Deciduous broadleaf forest (4)	-	-	-
Mixed forest (5)	-	-	-
Savanna/Shrublands (6-9)	10.27±1.95	-0.68±0.23	3.05±0.68
Grasslands (10,11)	15.86±3.7	-1.05±0.38	2.10±0.61
Croplands (12,16)	-	-	-

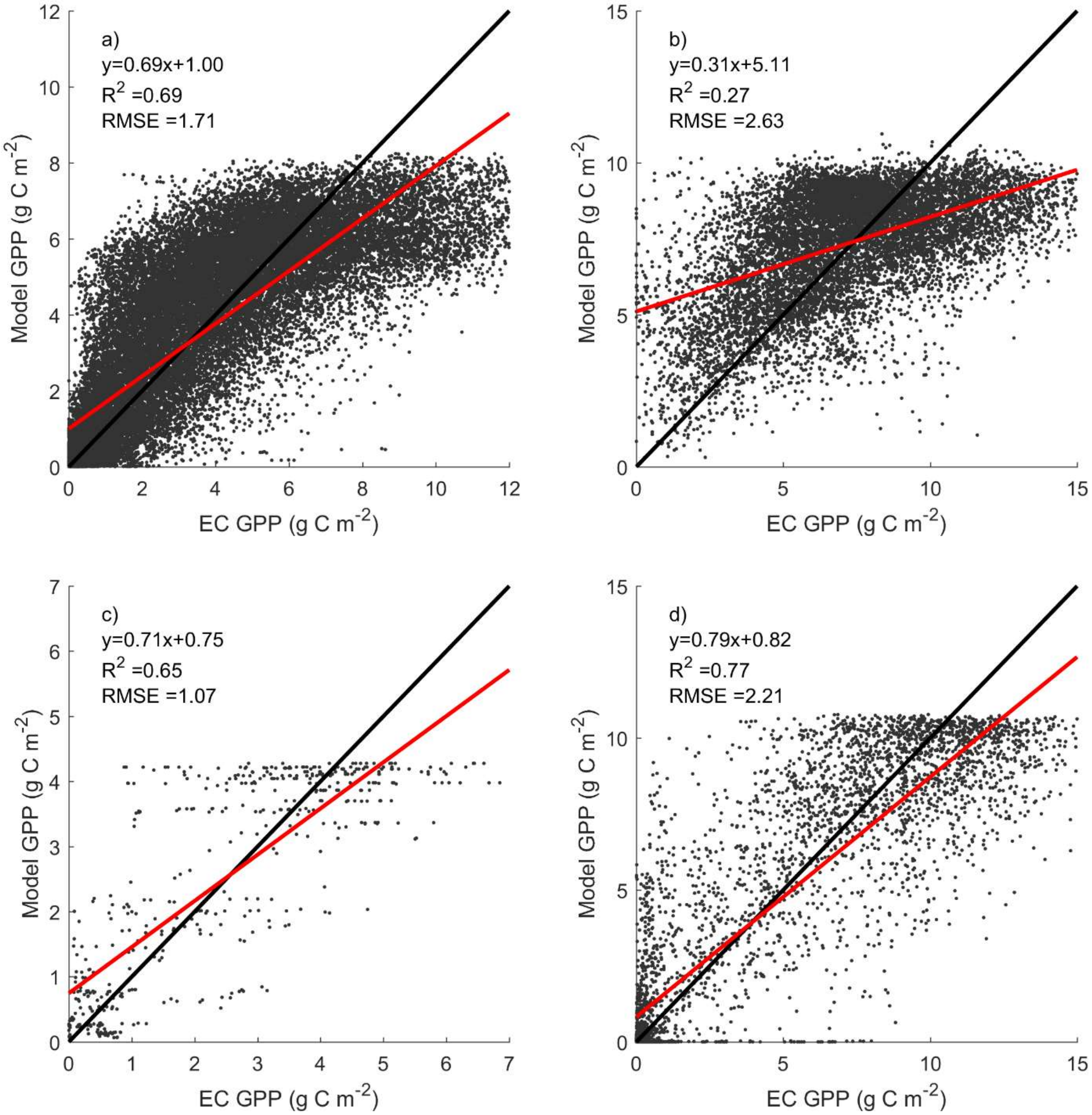
a)
y=0.69x+1.00
R2 =0.69
RMSE =1.71
Model GPP (g C m-2)
EC GPP (g C m-2)
b)
y=0.31x+5.11
R2 =0.27
RMSE =2.63
Model GPP (g C m-2)
EC GPP (g C m-2)
c)
y=0.71x+0.75
R2 =0.65
RMSE =1.07
Model GPP (g C m-2)
EC GPP (g C m-2)
d)
y=0.79x+0.82
R2 =0.77
RMSE =2.21
Model GPP (g C m-2)
EC GPP (g C m-2)

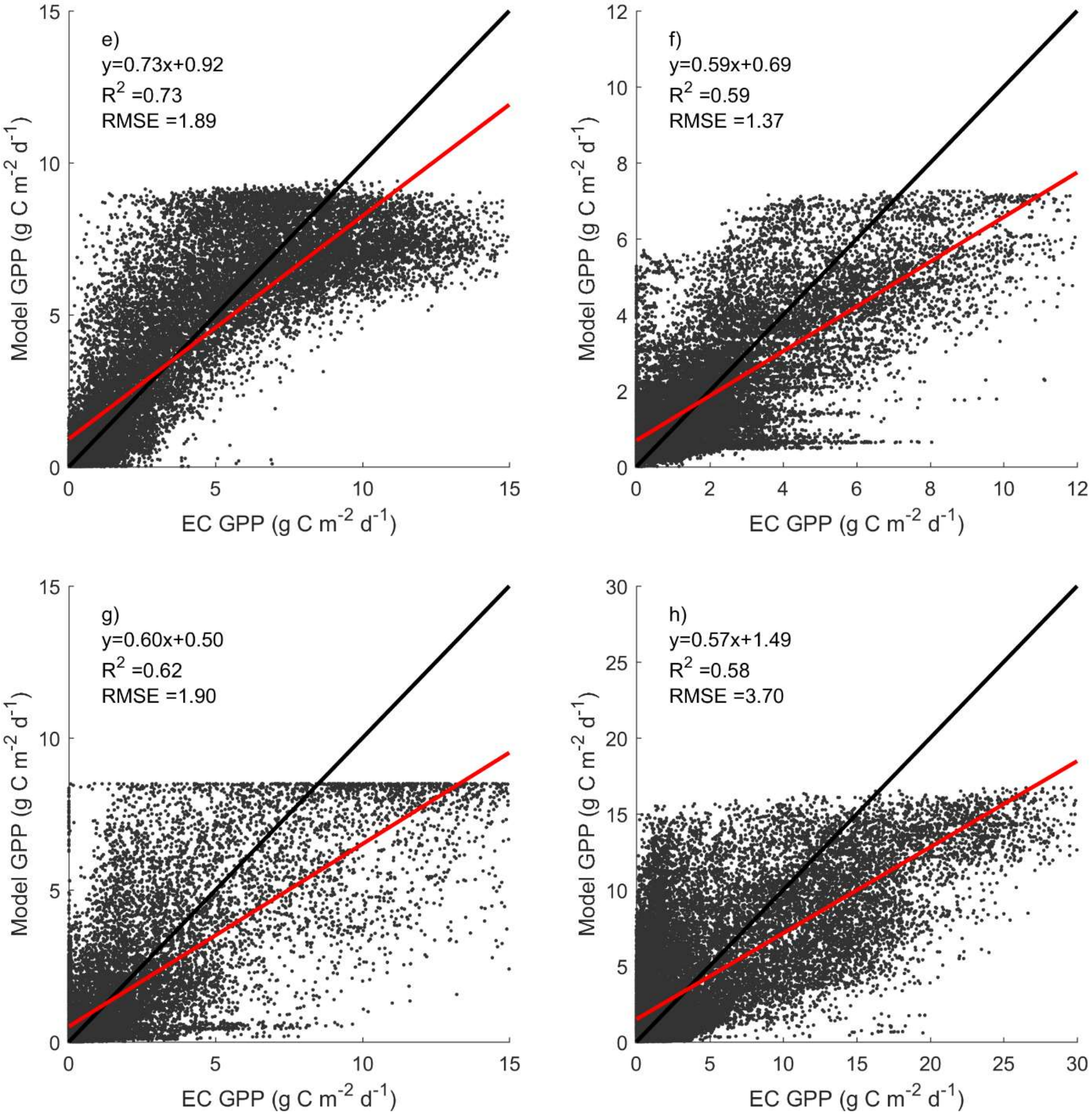

Figure S24 Modelled gross primary production (GPP) against eddy covariance (EC) based GPP for a) evergreen needleleaf forest; b) evergreen broadleaf forest; c) deciduous needleleaf forest; d) deciduous broadleaf forest; e) mixed forest; f) savanna/shrublands; g) grasslands; and g) croplands.

S7 Evaluation of global-scale Earth observation GPP products

S7.1 Evaluation against FLUXNET2015 GPP

Table S6 Evaluation of the different Earth Observation (EO) gross primary production (GPP) products against FLUXNET2015 GPP data for sites separated between the different biomes; evergreen needleleaf forest (ENF), evergreen broadleaf forest (EBF), deciduous needleaf forest

(DNF), deciduous broadleaf forest (DBF), mixed forest (MIX), savanna/shrublands (SAS), grasslands (GRA), croplands (CRO), and all sites. The uncertainty is ±one standard deviation.

	Mean EC GPP	Mean EO GPP	bias	RMSE	k	m	R^2
LRF model							
ENF	3.21±3.07	3.30±2.68	-0.00±1.67	1.68	0.73±0.003	0.95±0.011	0.70
EBF	7.27±3.07	6.58±1.75	0.69±2.66	2.74	0.28±0.005	4.48±0.037	0.26
DNF	2.45±1.81	2.38±1.4	0.08±1.05	1.05	0.63±0.02	0.82±0.062	0.66
DBF	4.58±4.65	6.21±4.69	-1.60±2.17	2.71	0.89±0.007	2.09±0.045	0.80
MIX	3.65±3.63	3.44±3.16	0.21±1.91	1.92	0.74±0.003	0.73±0.015	0.73
SAS	1.74±2.13	1.91±1.79	-0.10±1.3	1.31	0.66±0.003	0.74±0.008	0.63
GRA	1.83±3.02	1.53±2.36	0.3 ±1.87	1.89	0.61±0.003	0.40±0.012	0.62
CRO	3.61±5.71	2.98±3.46	0.64±3.63	3.69	0.48±0.002	1.24±0.013	0.63
All	3.22±4.03	3.07±3.08	0.16±2.37	2.37	0.62±0.001	1.06±0.006	0.66
FLUXCOM							
ENF	3.21±3.07	2.59±2.3	0.58±1.84	1.93	0.60±0.002	0.67±0.011	0.64
EBF	7.27±3.07	4.51±3.09	2.75±4.05	4.90	0.14±0.01	3.49±0.077	0.02
DNF	2.45±1.81	2.27±1.7	-0.00±1.02	1.02	0.80±0.03	0.48±0.085	0.68
DBF	4.58±4.65	3.24±2.55	1.34±2.87	3.16	0.45±0.005	1.14±0.032	0.69
MIX	3.65±3.63	3.12±3.08	0.53±2.13	2.20	0.68±0.003	0.60±0.017	0.65
SAS	1.74±2.13	1.44±1.49	0.31±1.45	1.48	0.51±0.003	0.54±0.008	0.55
GRA	1.83±3.02	1.04±1.57	0.85±2.04	2.21	0.41±0.002	0.26±0.007	0.66
CRO	3.61±5.71	2.53±2.66	1.09±4.3	4.43	0.32±0.002	1.33±0.012	0.51
All	3.22±4.03	2.39±2.56	0.84±2.84	2.97	0.45±0.001	0.92±0.006	0.52
Kolby Smith							
ENF	3.21±3.07	2.76±1.79	0.79±1.98	2.13	0.46±0.002	1.12±0.01	0.61
EBF	7.27±3.07	4.24±2.8	3.03±3.91	4.94	0.10±0.009	3.47±0.068	0.01
DNF	2.45±1.81	3.35±1.62	-0.80±1.13	1.40	0.70±0.026	1.57±0.079	0.62
DBF	4.58±4.65	3.26±2.07	1.32±2.95	3.23	0.39±0.003	1.44±0.02	0.79
MIX	3.65±3.63	2.42±2.34	1.35±2.63	2.96	0.44±0.003	0.74±0.016	0.48
SAS	1.74±2.13	1.10±0.85	0.65±1.69	1.81	0.26±0.002	0.63±0.005	0.45
GRA	1.83±3.02	1.40±1.43	1.01±2.55	2.74	0.31±0.002	0.64±0.009	0.58
CRO	3.61±5.71	2.03±1.66	1.61±4.75	5.01	0.19±0.001	1.31±0.008	0.47
All	3.22±4.03	2.20±1.99	1.23±3.17	3.40	0.31±0.001	1.11±0.005	0.43
p-model							
ENF	3.21±3.07	3.45±3.18	-0.2±2.36	2.37	0.74±0.004	1.05±0.017	0.51
EBF	7.27±3.07	4.66±2.76	2.61±3.94	4.72	0.08±0.008	4.07±0.067	0.01
DNF	2.45±1.81	4.34±2.61	-1.8±2.06	2.80	0.89±0.051	2.15±0.157	0.38
DBF	4.58±4.65	3.64±2.24	0.94±3.81	3.92	0.28±0.006	2.36±0.039	0.34
MIX	3.65±3.63	3.84±3.45	-0.1±2.59	2.60	0.69±0.004	1.29±0.021	0.54
SAS	1.74±2.13	1.79±1.38	-0.0±1.61	1.61	0.42±0.003	1.05±0.008	0.43
GRA	1.83±3.02	1.57±2.21	0.26±1.82	1.84	0.58±0.003	0.49±0.01	0.64
CRO	3.61±5.71	2.94±2.35	0.68±4.61	4.66	0.25±0.002	1.99±0.011	0.40

	All	3.22±4.03	2.93±2.79	0.29±3.12	3.13	0.44±0.001	1.51±0.007	0.40
MOD17								
	ENF	3.21±3.07	3.04±2.58	0.2 ±1.69	1.70	0.69±0.003	0.78±0.011	0.71
	EBF	7.27±3.07	5.93±1.54	1.34±2.97	3.26	0.15±0.004	4.77±0.035	0.10
	DNF	2.45±1.81	3.49±1.76	-1.0±1.13	1.53	0.77±0.026	1.58±0.081	0.64
	DBF	4.58±4.65	4.52±2.74	0.07±2.86	2.86	0.48±0.006	2.29±0.036	0.68
	MIX	3.65±3.63	3.29±2.76	0.35±1.91	1.94	0.65±0.003	0.92±0.013	0.74
	SAS	1.74±2.13	1.69±1.32	0.05±1.53	1.53	0.43±0.003	0.93±0.007	0.49
	GRA	1.83±3.02	1.17±1.6	0.68±2.04	2.15	0.41±0.002	0.40±0.008	0.63
	CRO	3.61±5.71	2.55±2.31	1.06±4.69	4.81	0.24±0.002	1.67±0.012	0.37
	All	3.22±4.03	2.69±2.47	0.53±2.86	2.91	0.43±0.001	1.28±0.006	0.52
SMAP								
	ENF	3.21±3.07	2.89±2.46	0.36±1.95	1.98	0.61±0.003	0.88±0.013	0.61
	EBF	7.27±3.07	6.38±2.55	0.9 ±3.09	3.22	0.34±0.007	3.90±0.057	0.17
	DNF	2.45±1.81	3.18±2.25	-0.7±1.63	1.78	0.86±0.04	1.05±0.123	0.49
	DBF	4.58±4.65	4.29±3.05	0.31±2.64	2.65	0.55±0.006	1.74±0.038	0.72
	MIX	3.65±3.63	3.51±3.26	0.14±2.2	2.20	0.71±0.004	0.88±0.018	0.64
	SAS	1.74±2.13	2.09±1.86	-0.3±1.47	1.51	0.64±0.003	0.97±0.009	0.54
	GRA	1.83±3.02	1.59±1.82	0.37±2.32	2.35	0.39±0.003	0.80±0.011	0.49
	CRO	3.61±5.71	3.7 ±3.84	-0.0±3.8	3.80	0.50±0.002	1.87±0.016	0.56
	All	3.22±4.03	3.14±3.07	0.12±2.63	2.63	0.57±0.001	1.26±0.006	0.59
VPM								
	ENF	3.21±3.07	2.68±2.74	0.57±1.72	1.81	0.73±0.003	0.29±0.012	0.70
	EBF	7.27±3.07	5.34±2.43	1.94±2.98	3.55	0.34±0.007	2.85±0.053	0.19
	DNF	2.45±1.81	3.23±2.6	-0.7±1.49	1.68	1.19±0.036	0.29±0.111	0.69
	DBF	4.58±4.65	5.86±5.22	-1.2±2.46	2.77	0.98±0.009	1.34±0.058	0.78
	MIX	3.65±3.63	4.01±4.33	-0.3±2.41	2.44	0.98±0.004	0.41±0.023	0.69
	SAS	1.74±2.13	2.15±2.45	-0.4±1.45	1.51	0.92±0.004	0.53±0.011	0.65
	GRA	1.83±3.02	1.33±2.08	0.52±2.17	2.23	0.48±0.003	0.44±0.012	0.49
	CRO	3.61±5.71	3.72±4.6	-0.1±3.46	3.46	0.64±0.003	1.40±0.018	0.63
	All	3.22±4.03	3.08±3.63	0.14±2.54	2.54	0.70±0.001	0.81±0.007	0.62

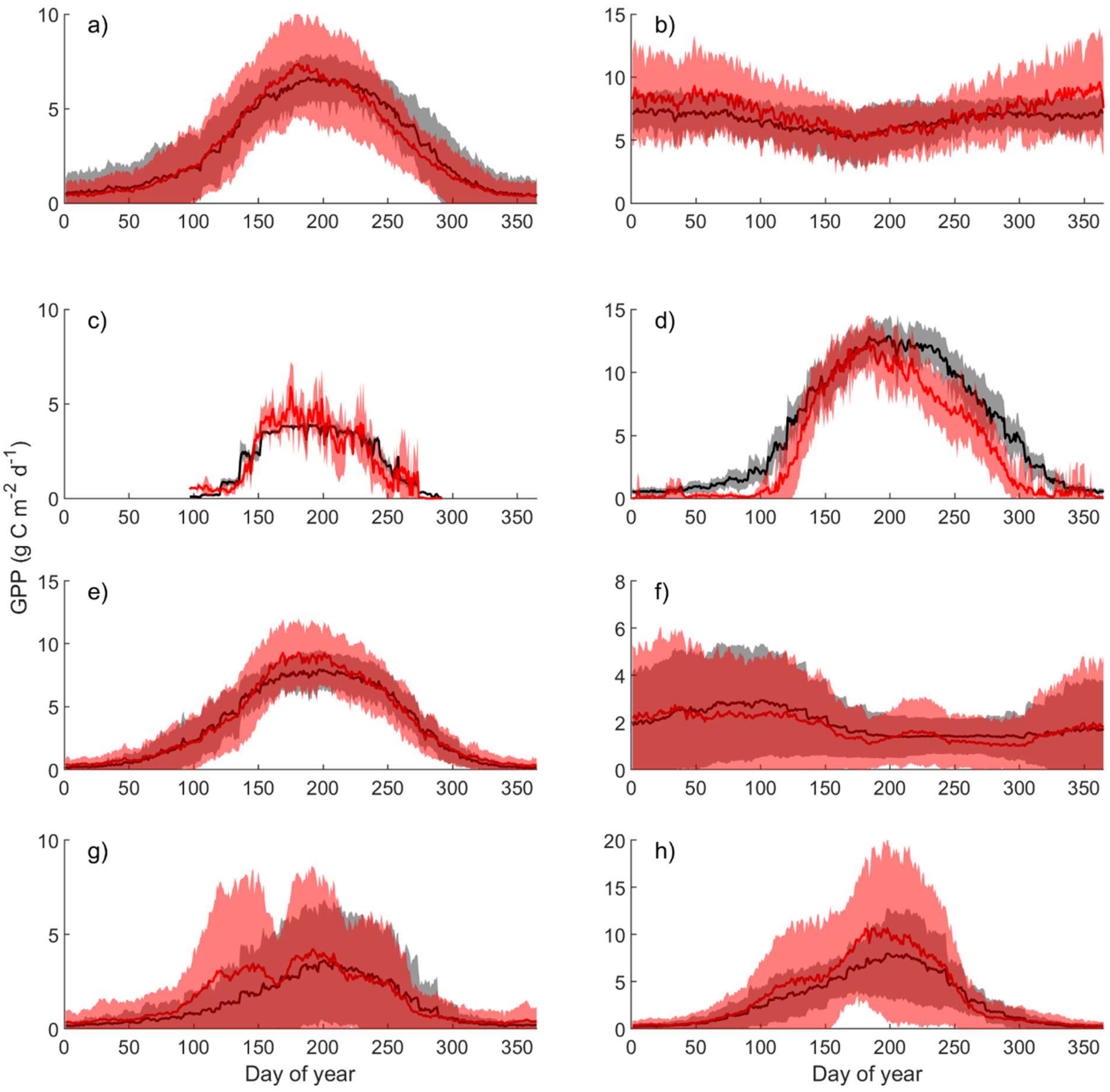

Figure 25 Evaluation of seasonal dynamics in modelled GPP (black) against GPP from the FLUXNET2015 sites (red) for the different biomes; a) evergreen needle leaf forest; b) evergreen broadleaf forest; c) deciduous needleleaf forest; d) deciduous broadleaf forest; e) mixed forest; f) savanna/shrublands; g) grasslands; and h) croplands. The uncertainty is ±one standard deviation.

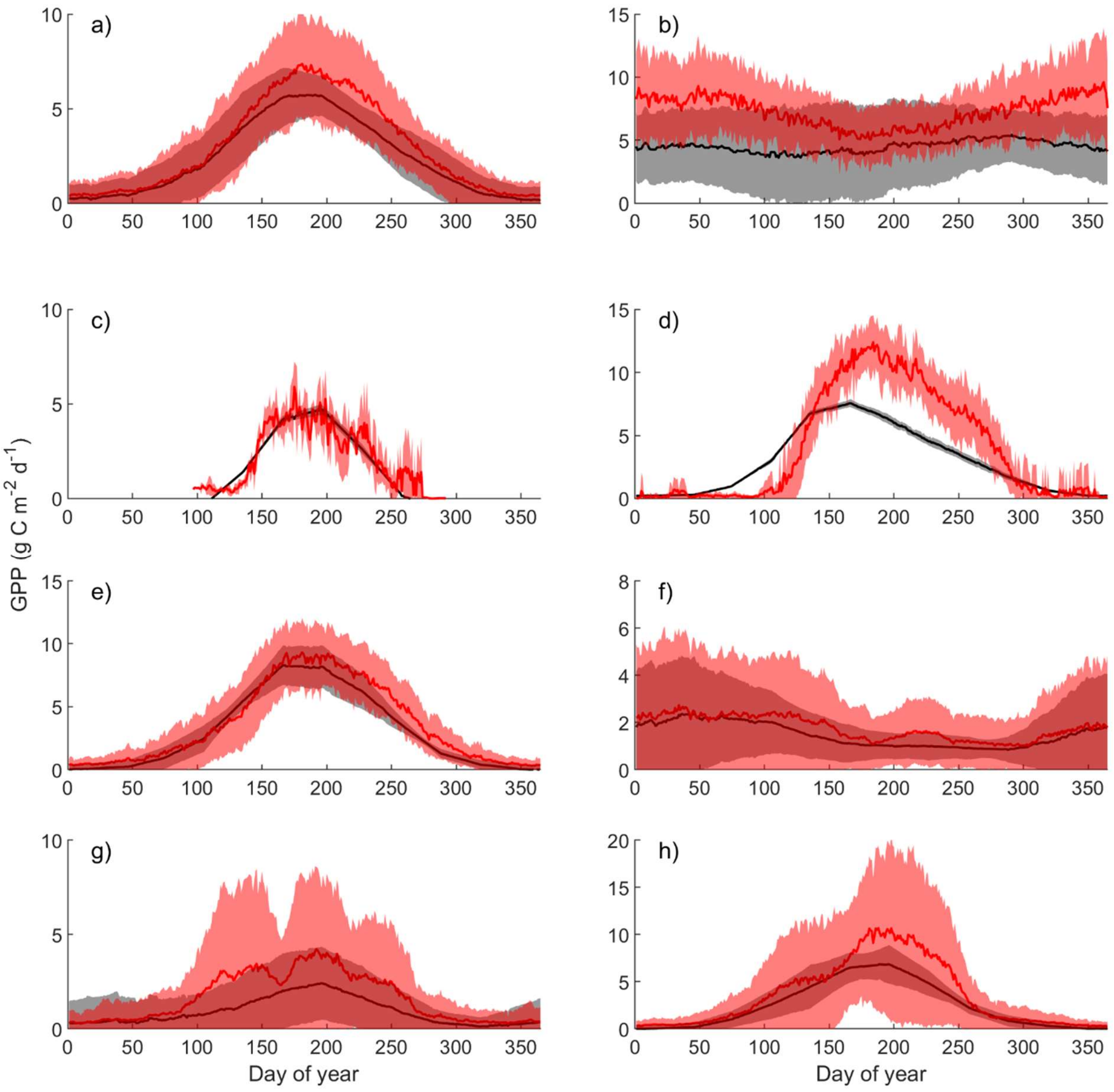

Figure S26 Evaluation of seasonal dynamics in FLUXCOM GPP (black) against GPP from the FLUXNET2015 sites (red) for the different biomes; a) evergreen needle leaf forest; b) evergreen broadleaf forest; c) deciduous needleaf forest; d) deciduous broadleaf forest; e) mixed forest; f) savanna/shrublands; g) grasslands; and h) croplands. The uncertainty is ±one standard deviation.

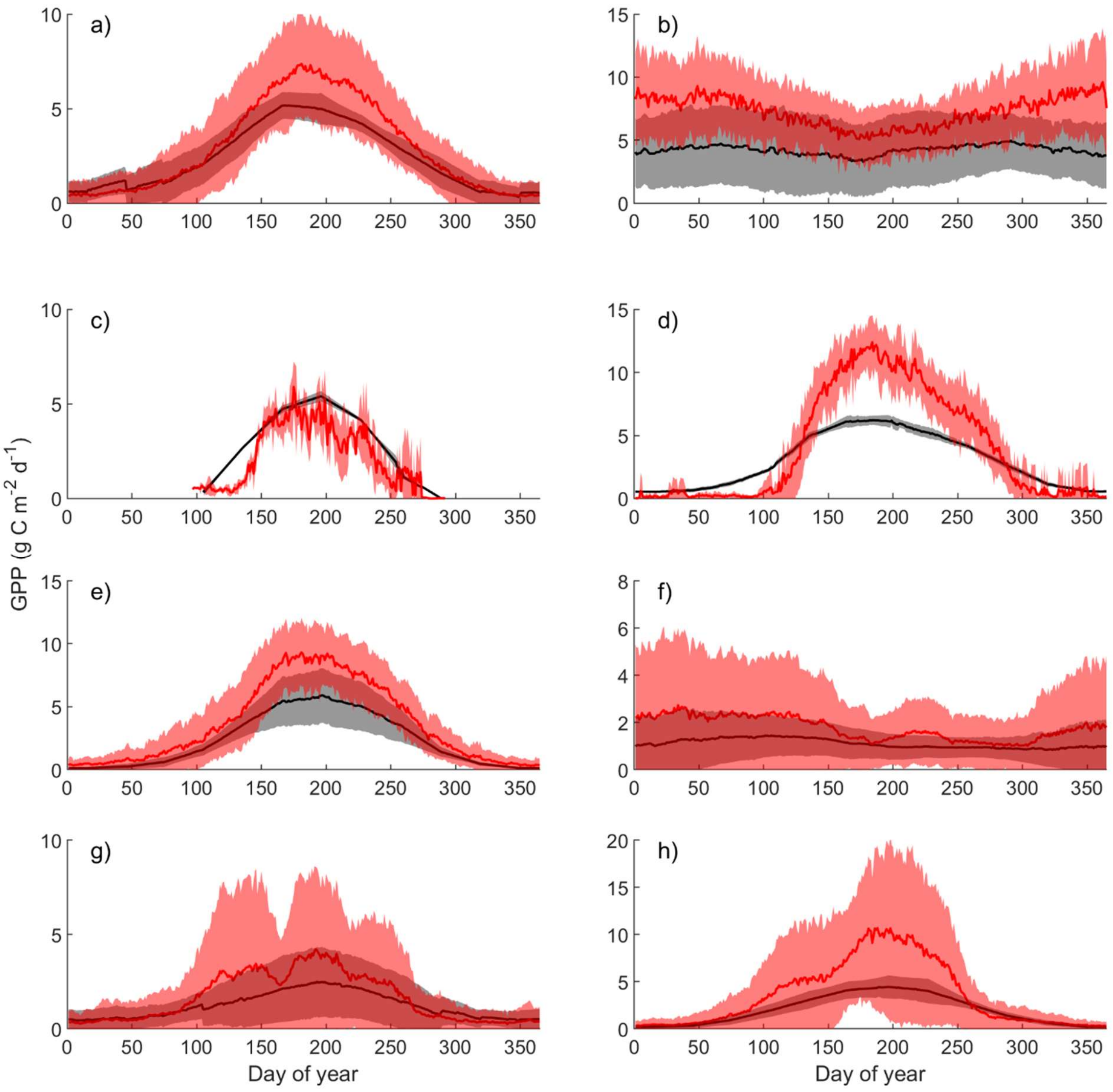

Figure S27 Evaluation of seasonal dynamics in Kolby Smith GPP (black) against GPP from the FLUXNET2015 sites (red) for the different biomes; a) evergreen needle leaf forest; b) evergreen broadleaf forest; c) deciduous needleaf forest; d) deciduous broadleaf forest; e) mixed forest; f) savanna/shrublands; g) grasslands; and h) croplands. The uncertainty is ±one standard deviation.

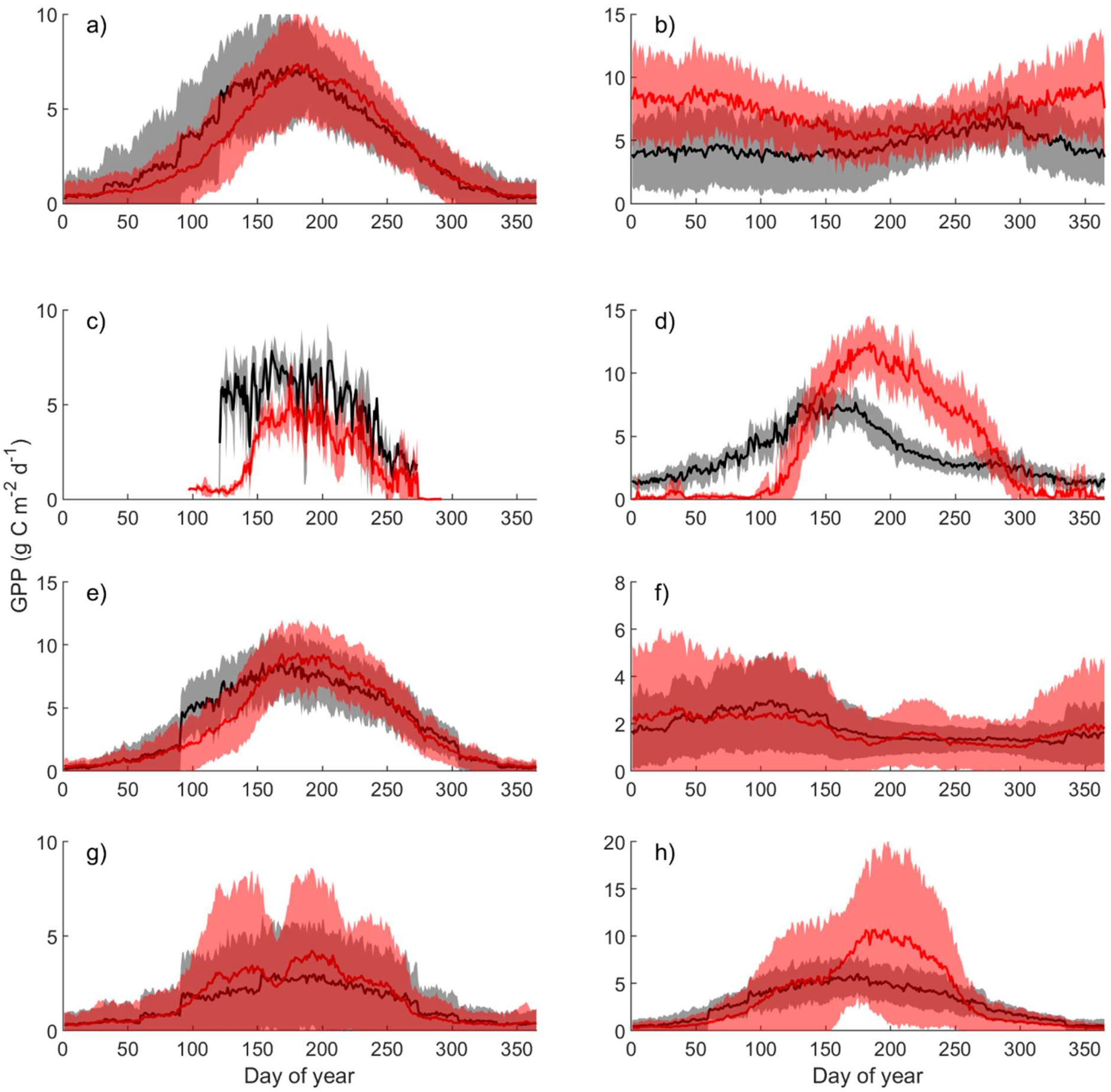

Figure S28 Evaluation of seasonal dynamics in p-model GPP (black) against GPP from the FLUXNET2015 sites (red) for the different biomes; a) evergreen needle leaf forest; b) evergreen broadleaf forest; c) deciduous needleaf forest; d) deciduous broadleaf forest; e) mixed forest; f) savanna/shrublands; g) grasslands; and h) croplands. The uncertainty is ±one standard deviation.

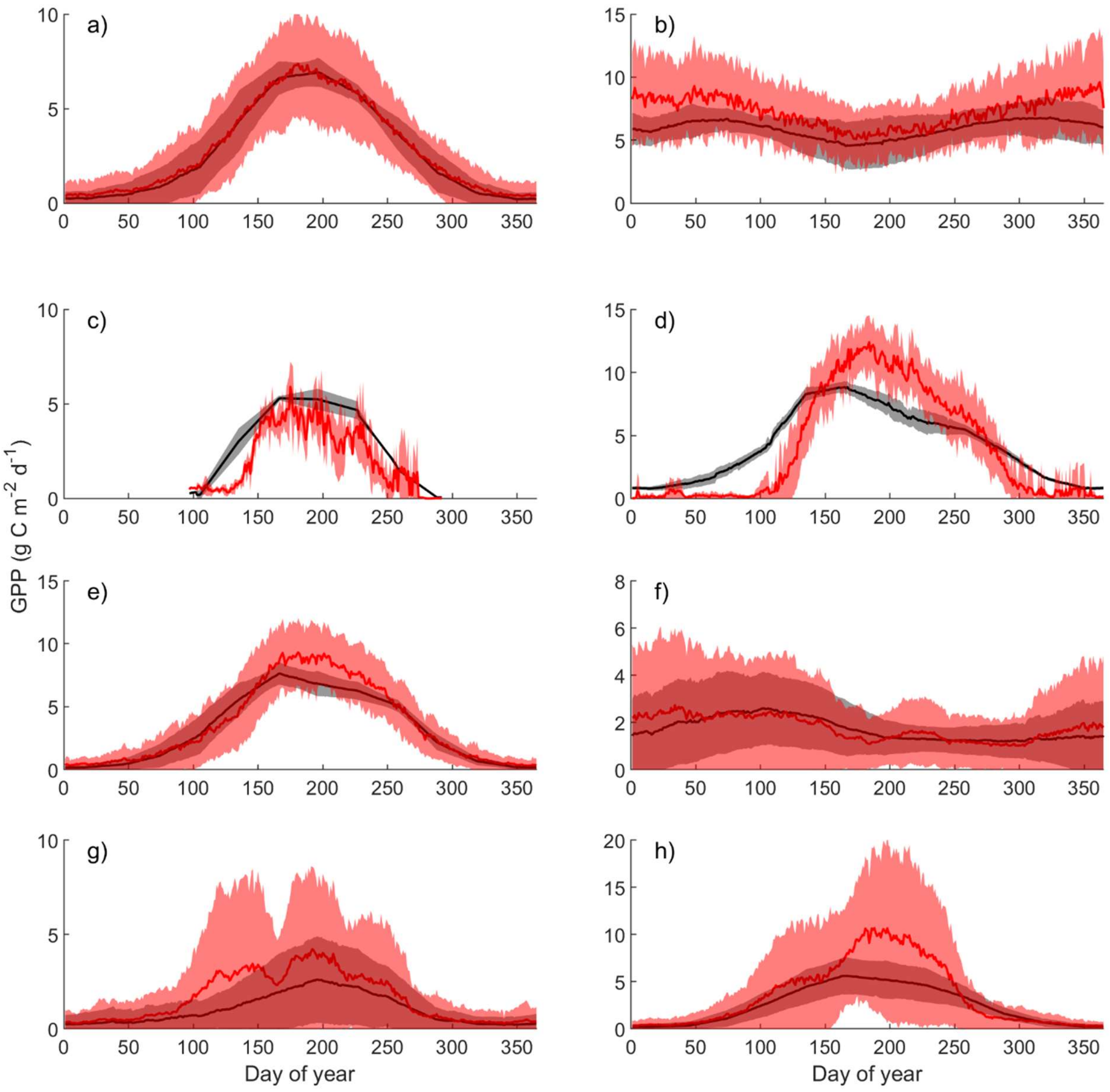

Figure S29 Evaluation of seasonal dynamics in MOD17 GPP (black) against GPP from the FLUXNET2015 sites (red) for the different biomes; a) evergreen needle leaf forest; b) evergreen broadleaf forest; c) deciduous needleaf forest; d) deciduous broadleaf forest; e) mixed forest; f) savanna/shrublands; g) grasslands; and h) croplands. The uncertainty is ±one standard deviation.

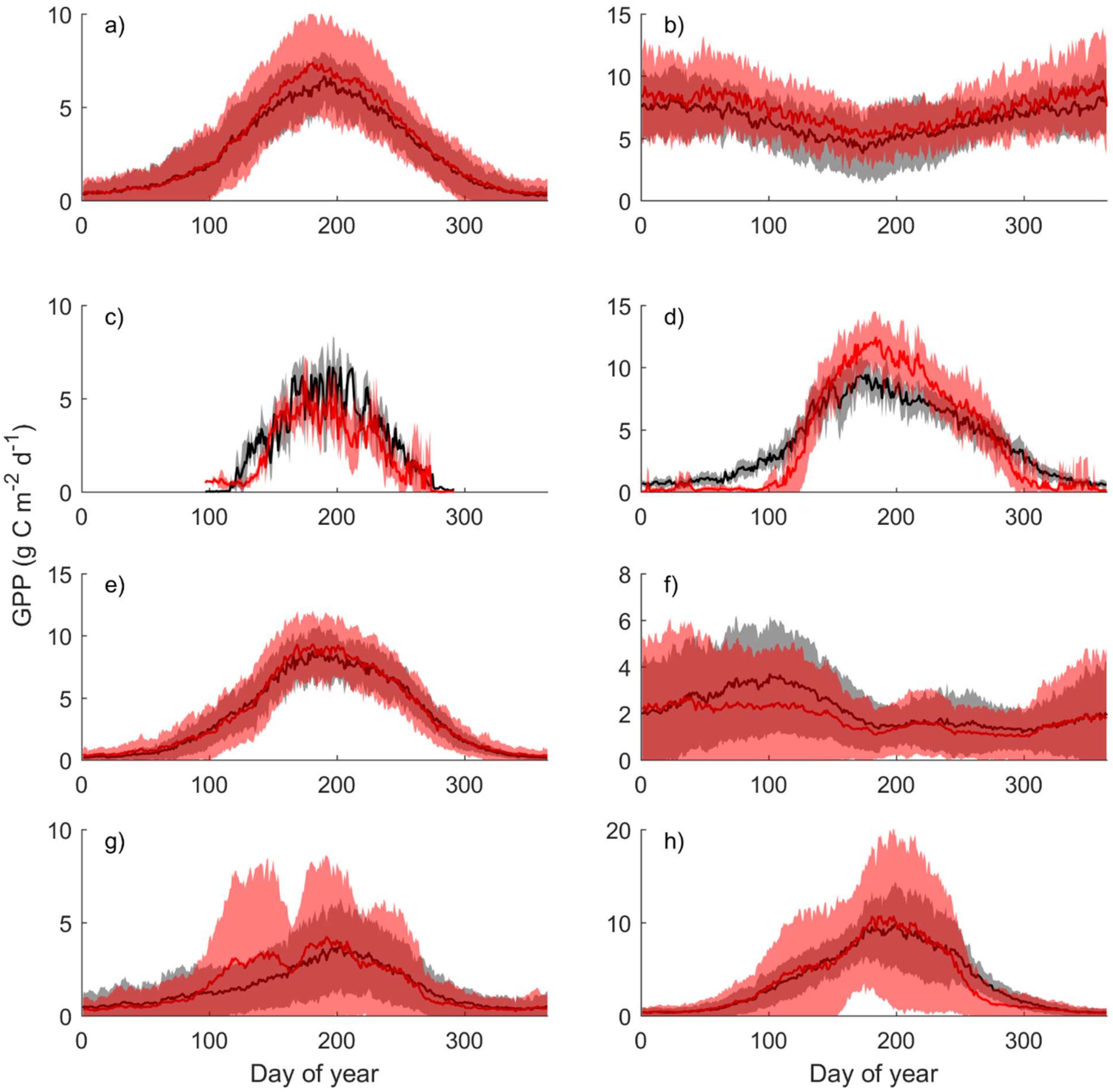

Figure S30 Evaluation of seasonal dynamics in SMAP GPP (black) against GPP from the FLUXNET2015 sites (red) for the different biomes; a) evergreen needle leaf forest; b) evergreen broadleaf forest; c) deciduous needleaf forest; d) deciduous broadleaf forest; e) mixed forest; f) savanna/shrublands; g) grasslands; and h) croplands. The uncertainty is ±one standard deviation.

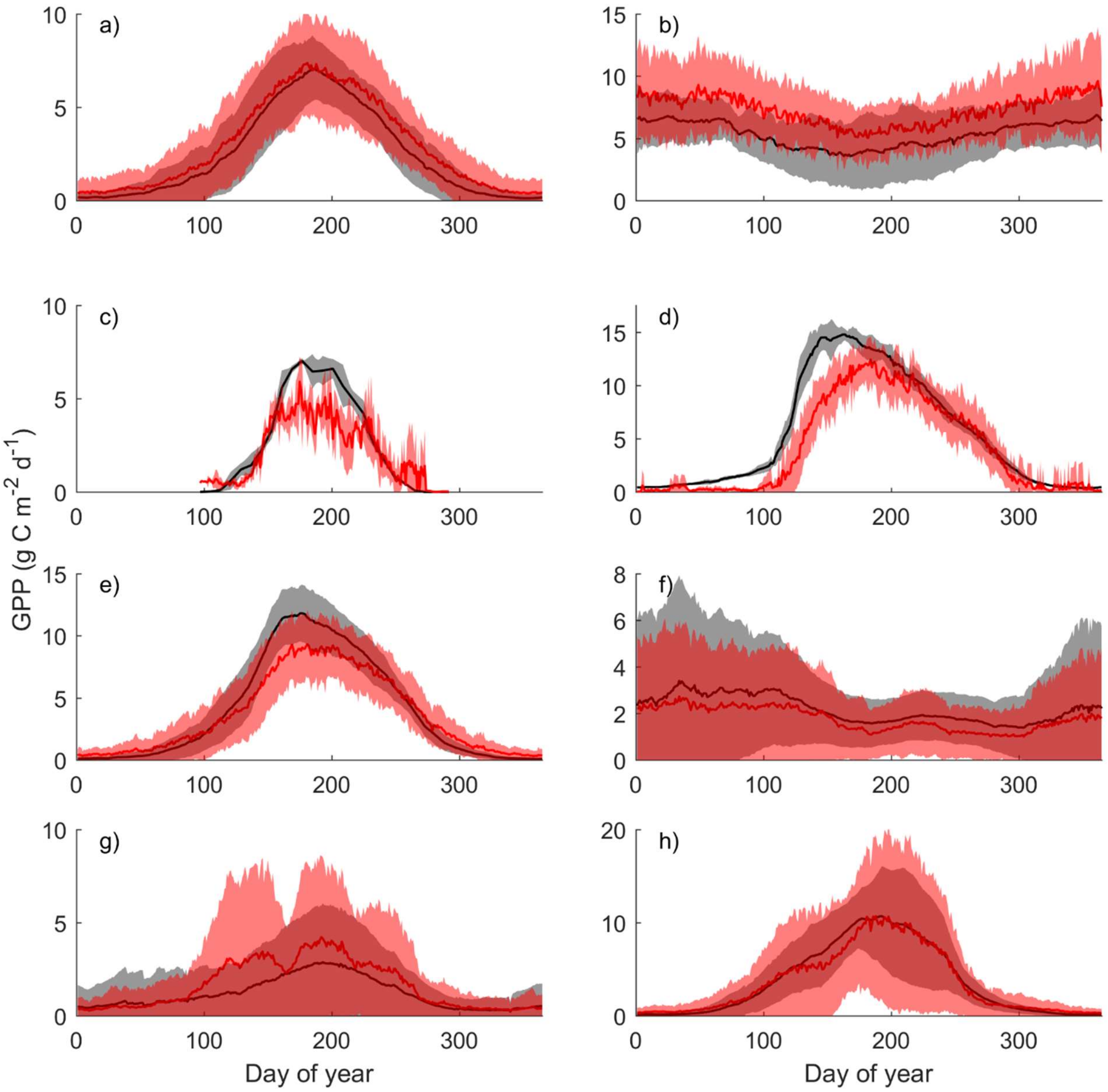

Figure S31 Evaluation of seasonal dynamics in VPM GPP (black) against GPP from the FLUXNET2015 sites (red) for the different biomes; a) evergreen needle leaf forest; b) evergreen broadleaf forest; c) deciduous needleaf forest; d) deciduous broadleaf forest; e) mixed forest; f) savanna/shrublands; g) grasslands; and h) croplands. The uncertainty is ±one standard deviation.

S7.2 Evaluation against SIF data extracted from the FLUXNET2015 sites

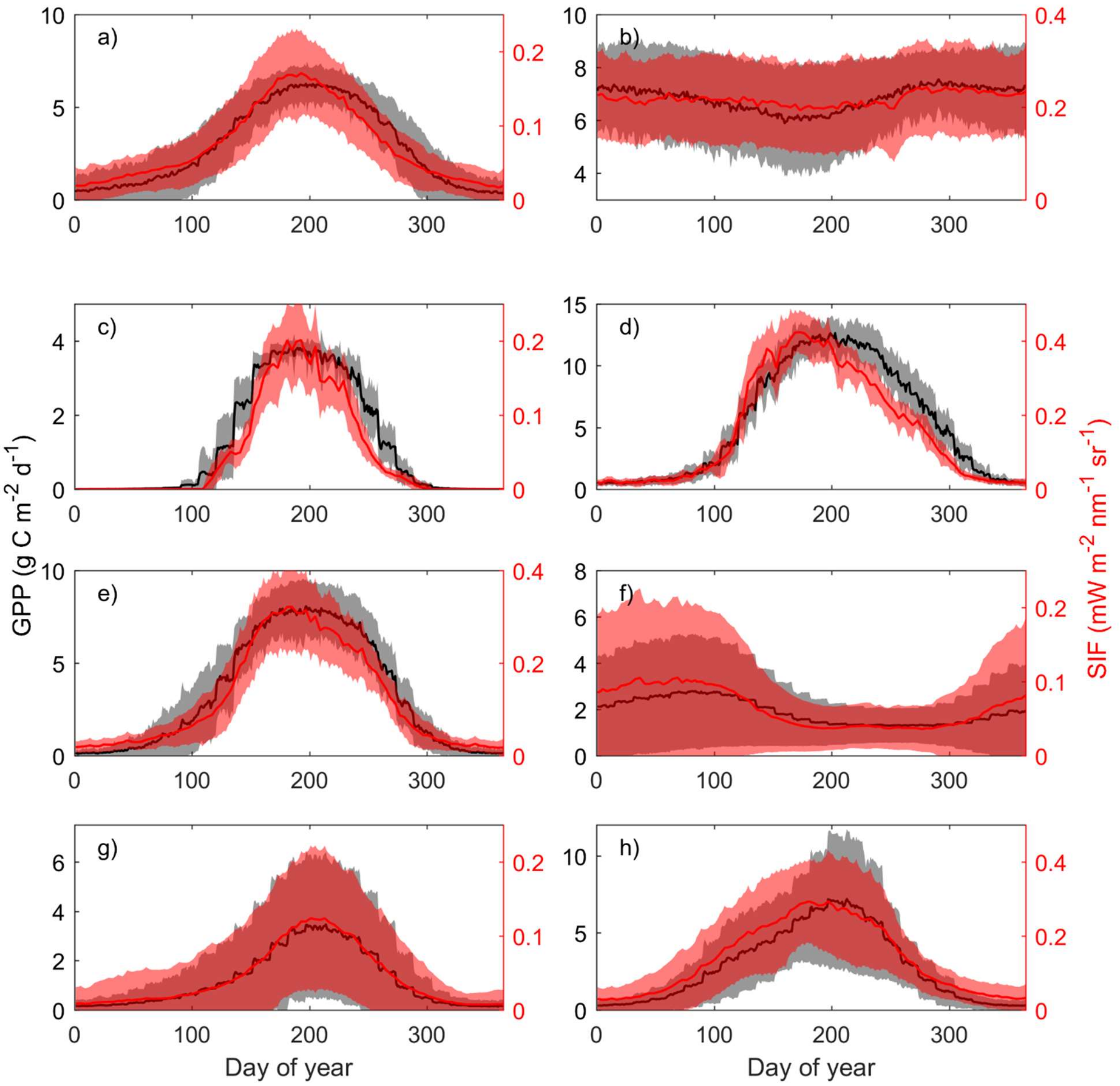

Figure 32 Evaluation of seasonal dynamics in modelled GPP (black) against Solar Induced Fluorescence (SIF; red) for the different biomes; a) evergreen needle leaf forest; b) evergreen broadleaf forest; c) deciduous needleaf forest; d) deciduous broadleaf forest; e) mixed forest; f) savanna/shrublands; g) grasslands; and h) croplands. The uncertainty is ±one standard deviation.

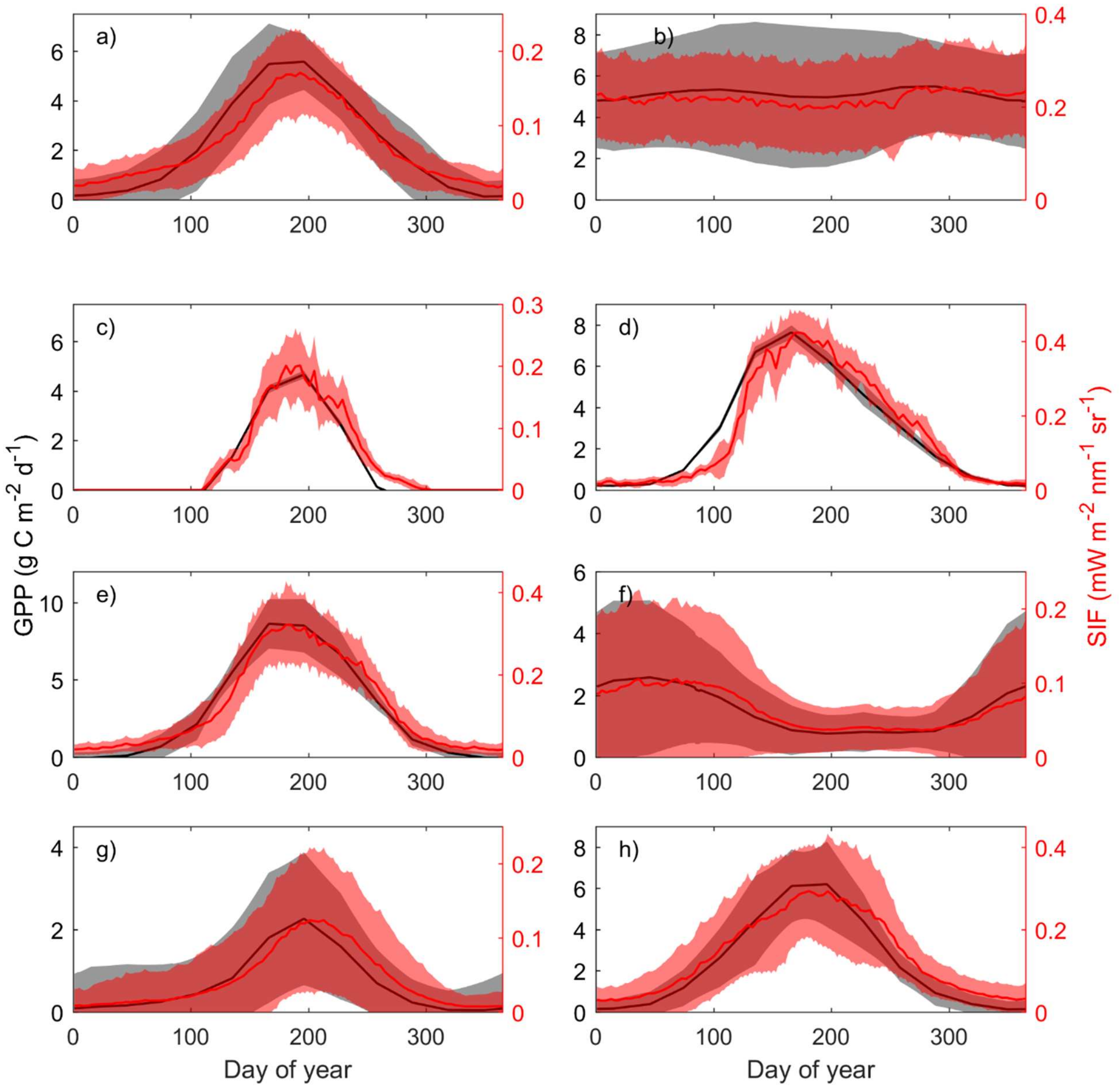

Figure S33 Evaluation of seasonal dynamics in FLUXCOM GPP (black) against Solar Induced Fluorescence (SIF; red) for the different biomes; a) evergreen needle leaf forest; b) evergreen broadleaf forest; c) deciduous needleaf forest; d) deciduous broadleaf forest; e) mixed forest; f) savanna/shrublands; g) grasslands; and h) croplands. The uncertainty is ±one standard deviation.

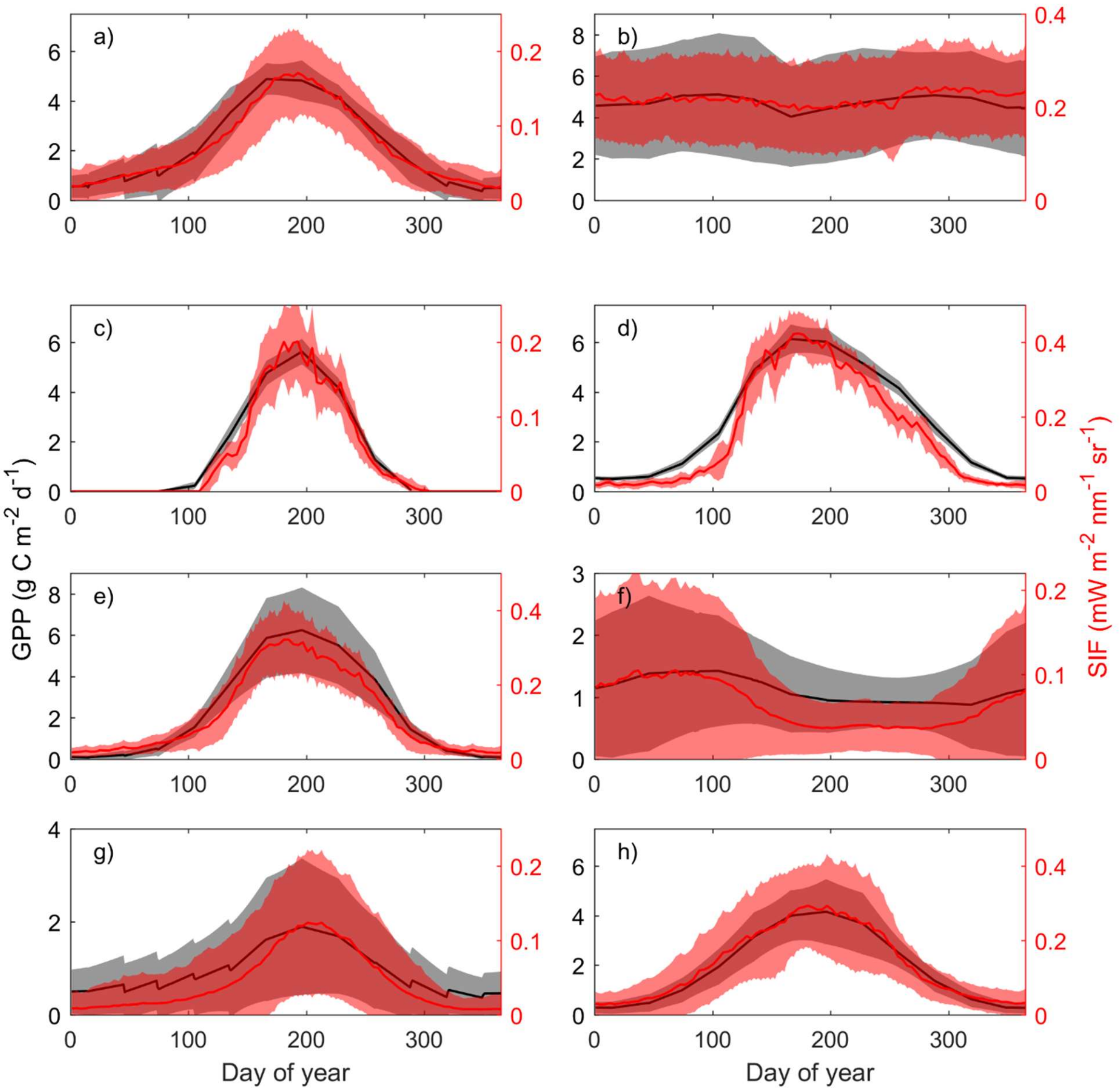

Figure S34 Evaluation of seasonal dynamics in Kolby Smith GPP (black) against Solar Induced Fluorescence (SIF; red) for the different biomes; a) evergreen needle leaf forest; b) evergreen broadleaf forest; c) deciduous needleaf forest; d) deciduous broadleaf forest; e) mixed forest; f) savanna/shrublands; g) grasslands; and h) croplands. The uncertainty is ±one standard deviation.

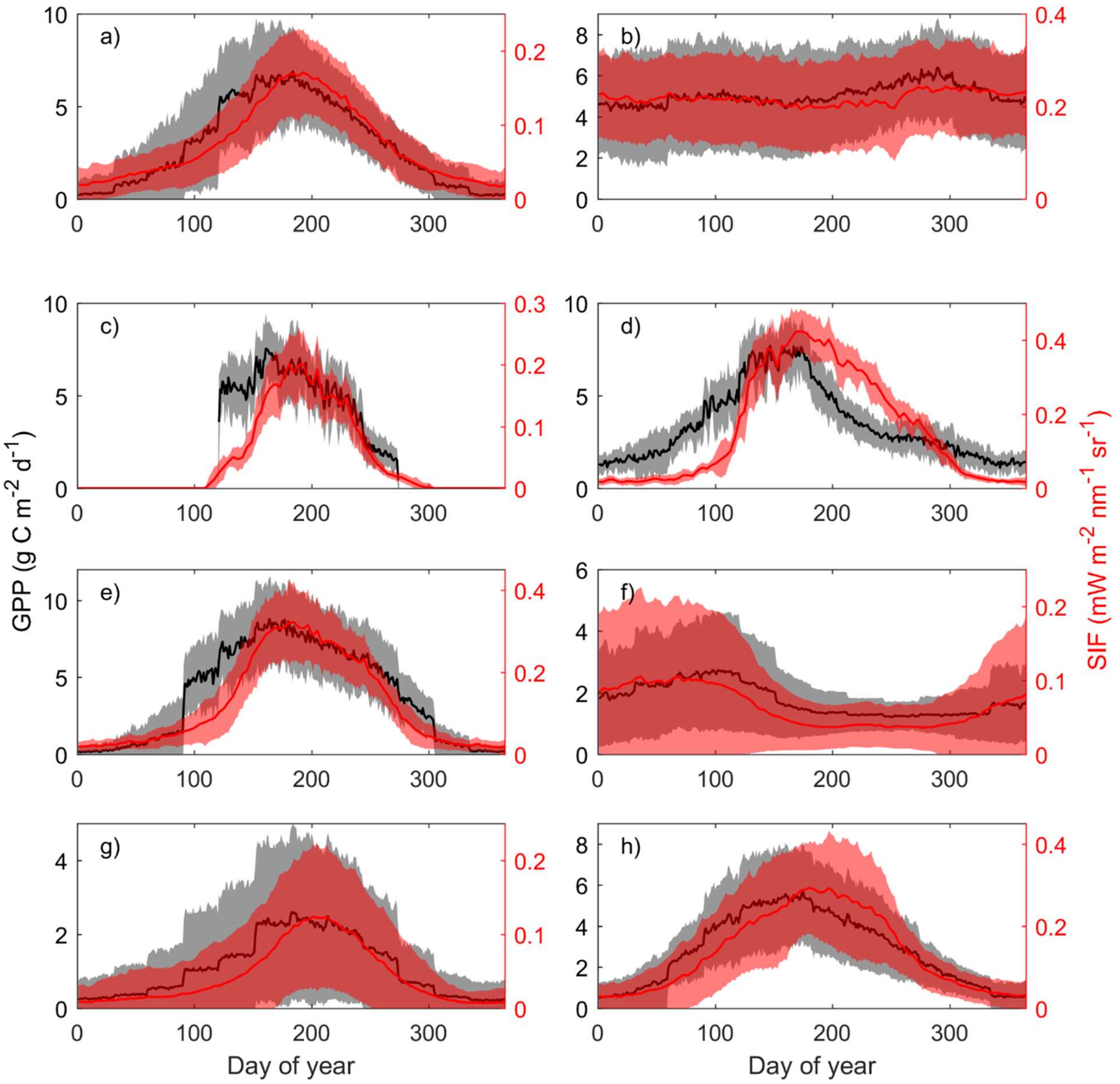

Figure S35 Evaluation of seasonal dynamics in the p-model GPP (black) against Solar Induced Fluorescence (SIF; red) for the different biomes; a) evergreen needle leaf forest; b) evergreen broadleaf forest; c) deciduous needleaf forest; d) deciduous broadleaf forest; e) mixed forest; f) savanna/shrublands; g) grasslands; and h) croplands. The uncertainty is ±one standard deviation.

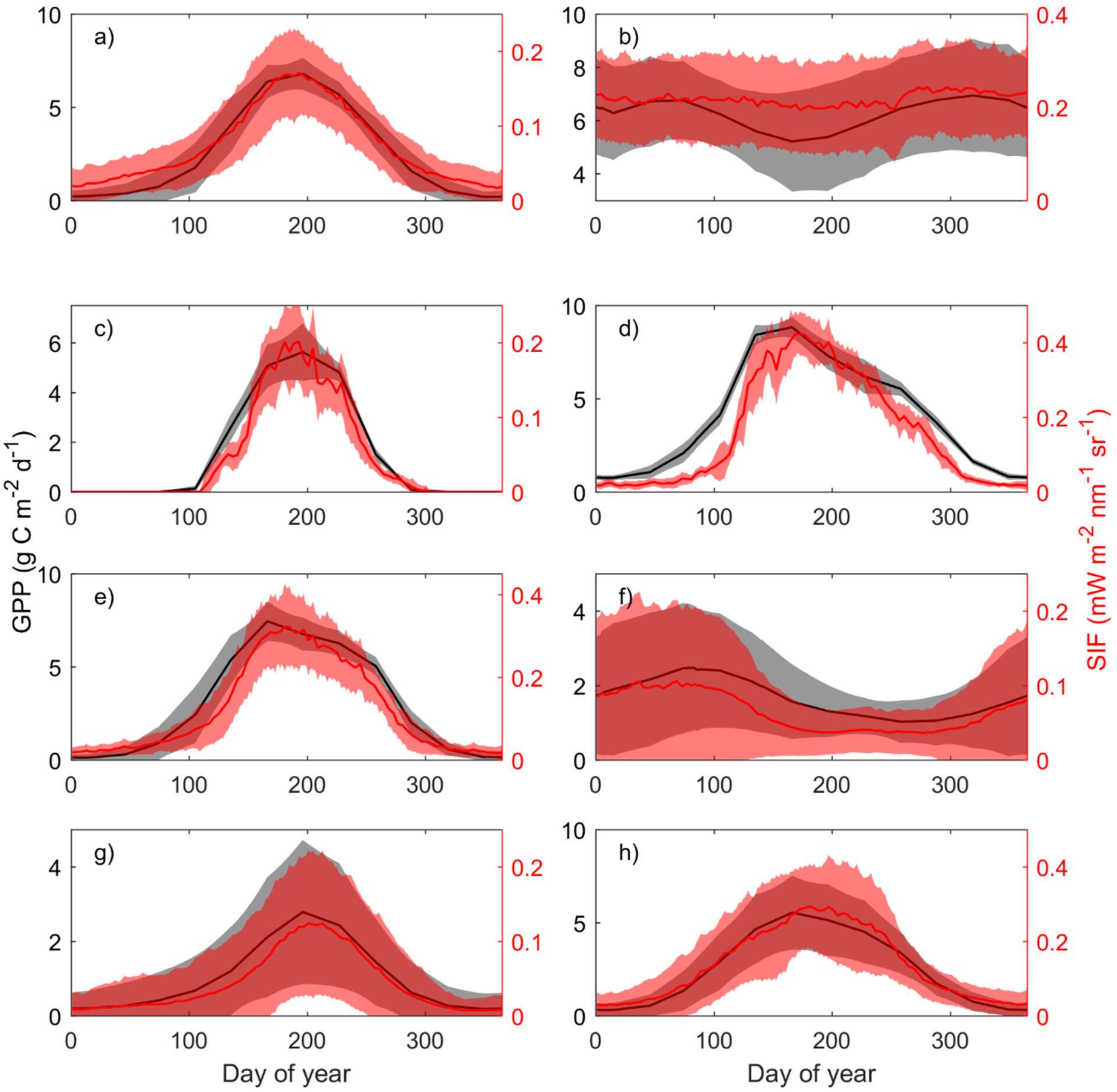

Figure S36 Evaluation of seasonal dynamics in MOD17 GPP (black) against Solar Induced Fluorescence (SIF; red) for the different biomes; a) evergreen needle leaf forest; b) evergreen broadleaf forest; c) deciduous needleaf forest; d) deciduous broadleaf forest; e) mixed forest; f) savanna/shrublands; g) grasslands; and h) croplands. The uncertainty is ±one standard deviation.

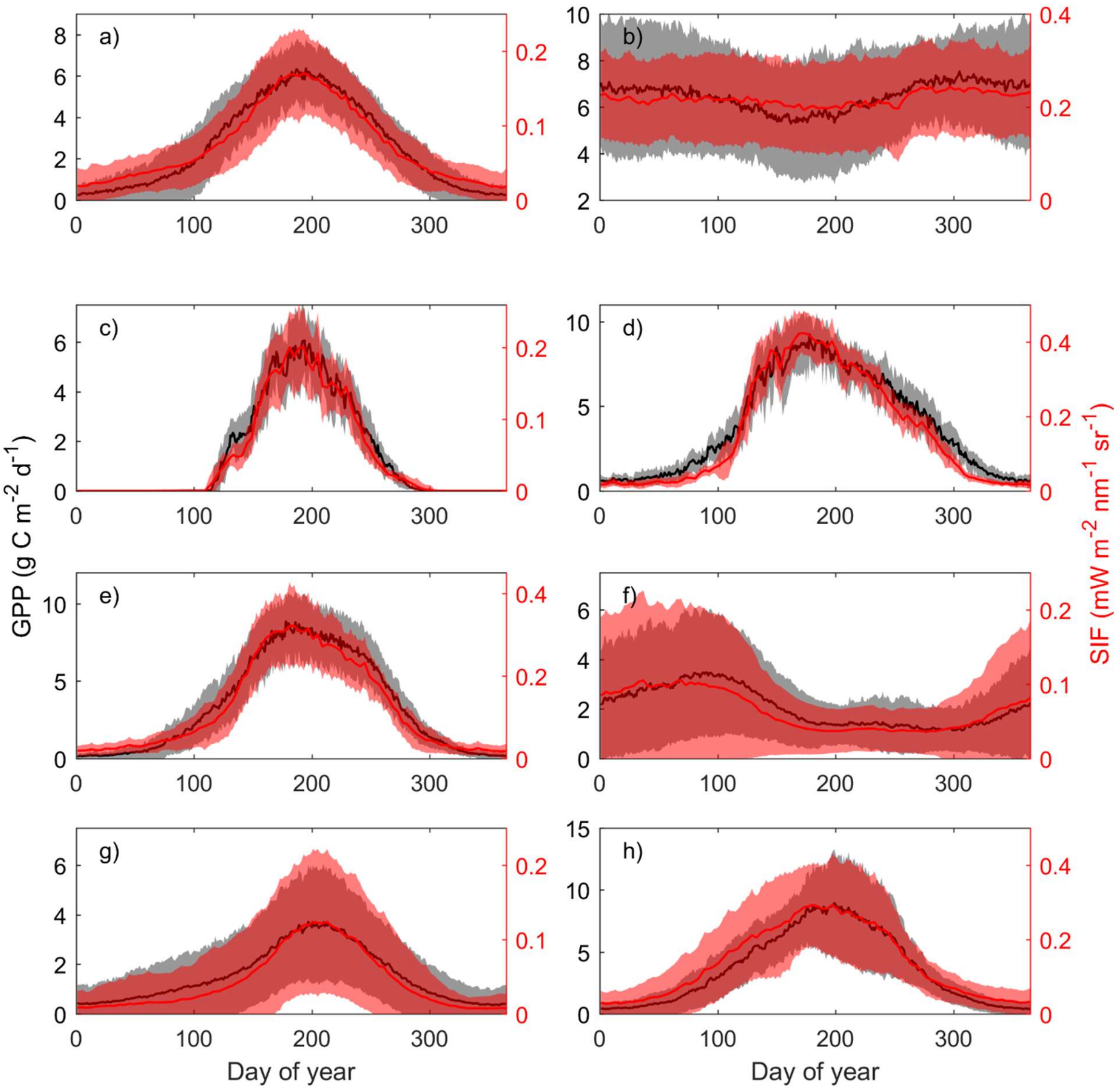

Figure S37 Evaluation of seasonal dynamics in SMAP GPP (black) against Solar Induced Fluorescence (SIF; red) for the different biomes; a) evergreen needle leaf forest; b) evergreen broadleaf forest; c) deciduous needleaf forest; d) deciduous broadleaf forest; e) mixed forest; f) savanna/shrublands; g) grasslands; and h) croplands. The uncertainty is ±one standard deviation.

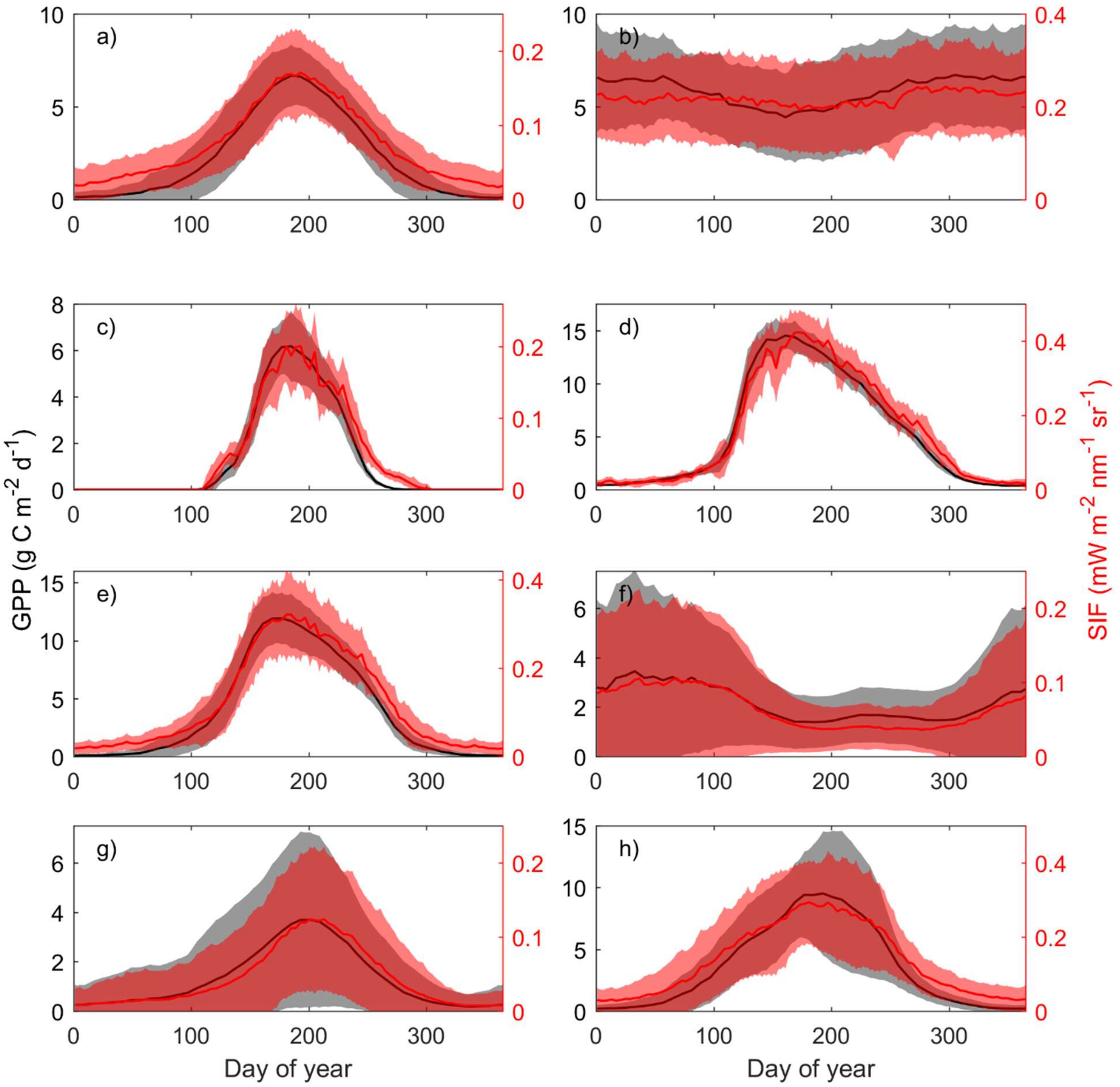

Figure S38 Evaluation of seasonal dynamics in VPM GPP (black) against Solar Induced Fluorescence (SIF; red) for the different biomes; a) evergreen needle leaf forest; b) evergreen broadleaf forest; c) deciduous needleaf forest; d) deciduous broadleaf forest; e) mixed forest; f) savanna/shrublands; g) grasslands; and h) croplands. The uncertainty is ±one standard deviation.

S7.1 Evaluation against GPPDI NPP

Table S7 Evaluation of the Earth observation modelled gross primary production (GPP) products using GIMMS NDVI against the the Global Primary Production Data Initiative (GPPDI) net primary production (NPP) data. The uncertainty is ±one standard deviation of spatial and inter-annual variability.

Models	GPP (g C $y^{-1)}$	NPP (g C $y^{-1)}$	k	m	R^2
LRF model	1199±537	609±344	0.63	814	0.16
FLUXCOM	1154±667	595±311	0.72	727	0.11
K Smith	942±570	600±311	0.6	584	0.11
p-model	1066±609	616±353	0.6	696	0.12

S8 Impact of land cover change

An important factor affecting the spatiotemporal dynamics in the LRF modelled GPP budgets are land cover changes (Tagesson *et al.*, 2020). In the current model set-up, land cover was set as static 2001 land cover from the MCD12C1 product. The main reason was that, as far as the authors are aware of, there is no dynamic land cover product covering the full study period. Other GPP products based on the GIMMS data also used static land cover in their GPP simulations (Jung *et al.*, 2011; Kolby Smith *et al.*, 2015; Stocker *et al.*, 2019).

In an attempt to study the impact of land cover change, we made an analysis of change in land cover 2001-2015, i.e. the period over which we have a dynamic land cover product (MCD12C1). The biome with the largest negative land cover change between 2001 and 2015 was evergreen broadleaf forest, whereas the most positive changes were seen for cropland, grassland, and savanna/shrublands (Fig S31b). To show the regions most influenced by land cover change, we subtracted the biome coverage percentage 2015 from that of 2001 (Fig S31c). The LRF-GPP simulations of the pixels with larger land cover changes (dark in Fig S39c) may be more uncertain than those with those with less land cover change (orange in Fig S39c).

We did a rough estimate of impact of land cover change on the global LRF-GPP budget by taking biome-wise average GPP based on the static land cover data set, and then applied these to the biomes of the dynamic land cover data (Fig S39d). The impact of land cover change on the average global GPP budget 2001-2015 was minor (annual GPP with static land cover: 123.95±1.33 Pg C (average±one standard deviation based on inter-annual variability); and with dynamic land cover: 123.55±1.29 Pg C). However, the impact of land cover change was larger on the estimated trends 2001-2015, (trend in GPP based on static land cover: 0.082±0.080 Pg C y^{-1}; and based on dynamic land cover: 0.042±0.079 Pg C y^{-1}). Still, both trends 2001-2015 were insignificantly different from 0, and there was no significant difference between the two estimates, indicating that the uncertainty by a lack of land cover change is within the inter-annual variability of the simulated GPP budgets.

On the other hand, it can clearly be seen that the GPP including a dynamic land cover is getting increasingly lower than static land cover GPP 2001-2015 (Fig S39d). Taking the difference between the two generates an estimate of impact of land cover change on global GPP 2001-2015. It is then clear that land cover change has had a significant impact on GPP 2001-2015 (-0.038±0.006 Pg C y^{-1}; R^2=0.74; p-value<0.001). For future studies on the impact of factors affecting the spatiotemporal GPP patterns, it is necessary to include a dynamic land cover data set in the LRF-GPP simulations.

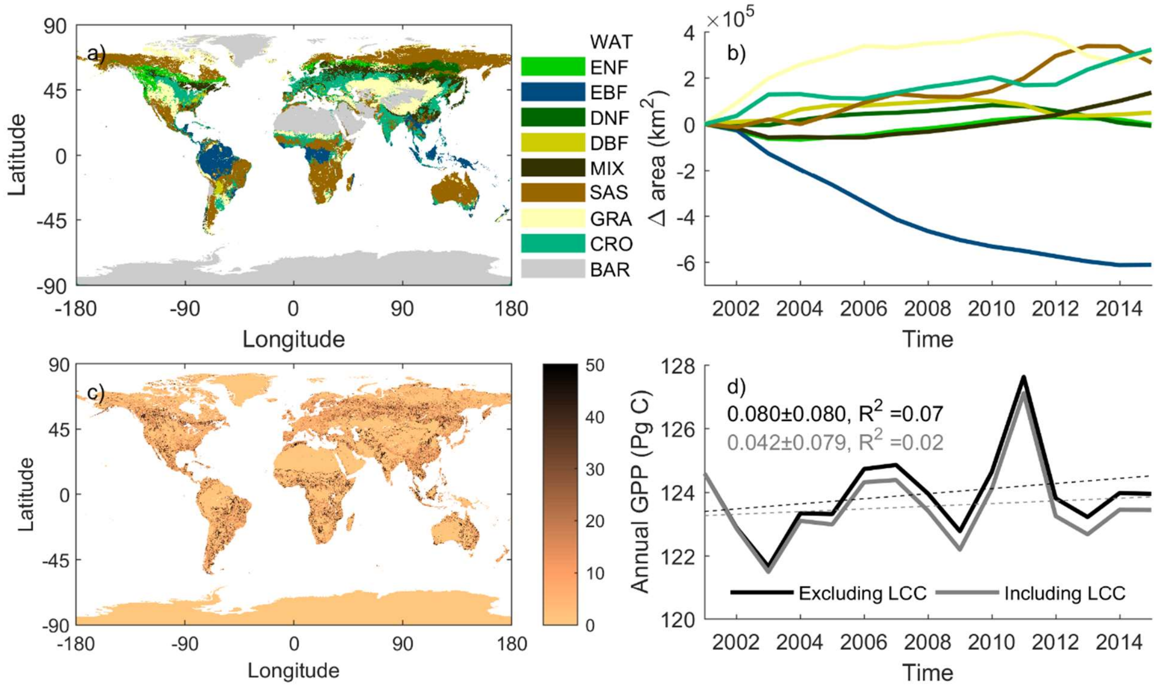

Figure S39 Impact of land cover change (LCC) 2000-2015 on light response function (LRF) modelled gross primary production (GPP). a) Distribution of biomes in 2001. b) Change in land area from 2001 to 2015 for the different biomes (evergreen needleleaf forest (ENF), evergreen broadleaf forest (EBF), deciduous needleaf forest (DNF), deciduous broadleaf forest (DBF), mixed forest (MIX), savanna/shrublands (SAS), grasslands (GRA), croplands (CRO), and bare land (BAR)). c) Percentage of the pixels with a land cover change between 2001 and 2015. d) Impact of land cover change on the global annual GPP budgets 2001-2015.

References

De'ath G, Fabricius KE (2000) Classification and regression trees: A powerful yet simple technique for ecological data analysis. *Ecology,* **81**, 3178-3192.

Jung M, Reichstein M, Margolis HA *et al.* (2011) Global patterns of land-atmosphere fluxes of carbon dioxide, latent heat, and sensible heat derived from eddy covariance, satellite, and meteorological observations. *Journal of Geophysical Research: Biogeosciences,* **116**, n/a-n/a.

Kolby Smith W, Reed SC, Cleveland CC *et al.* (2015) Large divergence of satellite and Earth system model estimates of global terrestrial CO_2 fertilization. *Nature Climate Change,* **6**, 306.

Stocker BD, Zscheischler J, Keenan TF, Prentice IC, Seneviratne SI, Peñuelas J (2019) Drought impacts on terrestrial primary production underestimated by satellite monitoring. *Nature Geoscience,* **12**, 264-270.

Tagesson T, Schurgers G, Horion S *et al.* (2020) Recent divergence in the contributions of tropical and boreal forests to the terrestrial carbon sink. *Nature Ecology & Evolution,* **4**, 202-209.
